# Assessment of Acute Stress and Chronic Stress for Mental Health Management by a Fully Integrated Wearable Biosystem

**DOI:** 10.1021/acsnano.5c12395

**Published:** 2025-11-06

**Authors:** Hongwei Chu, Wooyoung Park, Yue Hu, Zhenhe Huang, Rui Shi, Jiyu Li, Zhiyuan Li, Mengge Wu, Yuyu Gao, Guangyao Zhao, Xingcan Huang, Chun Ki Yiu, Binbin Zhang, Dengfeng Li, Kuanming Yao, Jian Li, Yuan Guo, Ya Huang, Qiuqian Ou, Guoqiang Xu, Pengcheng Wu, Jiao Yang, Yingchun Li, Xinge Yu

**Affiliations:** † Department of Biomedical Engineering, 529484 Harbin Institute of Technology (Shenzhen), Shenzhen 518055, China; ‡ Department of Biomedical Engineering, 53025 City University of Hong Kong, Kowloon, Hong Kong 999077, China; § School of Pharmacy, Shenzhen University Medical School, Shenzhen University, Shenzhen 518060, China; ∥ Department of Geriatrics, Xiehe Shenzhen Hospital, Huazhong University of Science and Technology, Shenzhen 518052, China; ⊥ Hong Kong Centre for Cerebro-Cardiovascular Health Engineering, Hong Kong Science Park, New Territories, Sha Tin, Hong Kong 999077, China; # Institute of Digital Medicine, City University of Hong Kong, Kowloon, Hong Kong 999077,China; ∇ Hong Kong Institute for Clean Energy (HKICE), City University of Hong Kong, Kowloon, Hong Kong 999077, China

**Keywords:** flexible electronics, wearable biosensor, sweat sensor, mental stress, health management

## Abstract

Mental stress has been a paramount concern worldwide, serving as a critical element for the optimal functioning and thriving of families, communities, and societies. However, the evaluation of the mental stress state in current clinical practice relies on symptom criteria associated with self-reported psychometric tools and behavioral manifestations, which raises the concerns of both accuracy issue and difficulty in identifying classifications. Continuous and quantitative assessments of mental stress-related physical/biomarkers based on wearable technologies is a very feasible way to solve the hurdle, while the implementation of such technology at clinical scales remains quite challenging and has yet to be documented. Herein, we report the collection of advances in sensing strategies, system integration, and model construction for mental health monitoring and management. A set of physical/biomarkers (cortisol, glucose, body temperature, and heart rate) are selected based on the systematic studies on the stress-correlated physiological pathways to form the marker panel. Corresponding stable and sustainable sensing systems are established upon the optimization of nanomaterials and nanostructure design. Comprehensive indicators are further generated based on algorithm bonuses for acute and chronic stress assessment. The fully integrated reusable wearable biosystem demonstrates its effectiveness in identifying, quantifying, and tracking the prognosis of stress in paradigmatic stress scenarios and clinical applications of social anxiety disorder/depression. The technology reported here presents a promising solution for customized mental stress management to facilitate the development of healthcare in psychological well-being.

## Graphical abstract

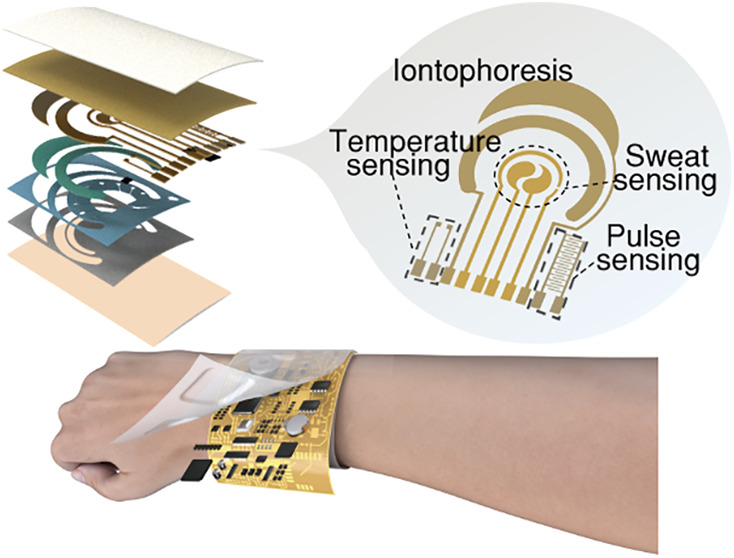

Mental health is integral to human well-being and of comparative significance with physiological health.^1–4^ Healthcare in mentality has become a global priority and received growing attention, which is especially highlighted by the increasing social burden.^5–7^ Stress, referring to the systemic, adaptive response that occurs when the body is subjected to a certain intensity of stress factors, plays a crucial role in the mental health dynamics of the human body.^8^ Stress can be aroused by diverse and omnipresent stimuli from physiological and psychological aspects, and creep into lives of expansive individuals like laborers, programmers, homemakers, and researchers.^9,10^ Excessive stress can cause severe damage to the body and culminate in an array of health complications, prominently featuring mental disorders such as clinical depression, post-traumatic stress disorder (PTSD), acute stress reaction (ASR), as well as physical ailments including stress ulcers and immune system dysfunctions.^11^ Effective comprehension and management of stress have become urgent and pivotal concerns for human mental health care (Note S1).

Stress is typically classified into acute stress (AS) and chronic stress (CS), with considerable distinction in terms of the triggering factors, functional characteristics, stimulus intensity, and somatic effects.^11–13^ To date, scientifically and accurately recognizing and quantifying mental stress is still a challenge. Clinical assessment of stress typically relies on questionnaire metrics and references to pathological behaviors, which can be subjective, experience-oriented, and thus quite limited for complex stress state analysis with scientific rigor.^14,15^ Monitoring the stress-triggered physiological cascade responses offers a more direct and reliable approach to assessing stress stimuli, free from the influence of subjective cognitive biases. Stress response restores homeostasis and promotes self-protection through intricate interactions among the nervous, endocrine, and immune systems, leading to certain changes in metabolic levels and organ functionalities.^16–19^ Predominantly, hormones play a dominant regulatory role throughout the stress response, accompanied by a series of alterations in physiological activities such as blood glucose metabolism, cardiac output, and thermal balance.^19,20^ Such changes could offer valuable insights into the complex dynamics of mental stress (Note S2).

Wearable biosensors can be a promising approach for health state assessment in a noninvasive and real-time manner, which has attracted great attention for developing wearable stress monitoring technologies (Note S3).^21–34^ Most of these studies focus on the methodological sensitivity analysis of one or two potential biomarkers, like electrodermal activity (EDA) and cortisol.^21–24^ However, it remains to be challenging for applying these technologies to achieve effective clinical stress management due to the following four aspects: (1) Sufficiently scientific biomarker panel, as this issue is mainly attributed to the difficulties in continuous detection of valuable biomarkers (like cortisol) and multiple sensor array integration; (2) Discriminated analysis of AS and CS, as the significant distinctions in characteristics and underlying mechanisms between these two types of stress call for a specific and tailored analysis. (3) Establishment of a comprehensive indicator of stress mapping with considerable application reliability, as which is crucial to clarify the correlation between the biomarker panel and stress states, as well as to minimize the impact of interindividual and intraindividual variances on actual assessment performance; (4) Practical evaluation within extensive clinical settings, since successfully validating the metrics in a large cohort of individuals with specific mental disorders is a critical and necessary step for the transition from the laboratory to clinics.

In this work, we report a fully integrated reusable electronic system (FIRES) for proactive and customized mental stress management. Four markers (cortisol, glucose, skin temperature (ST), and heart rate (HR)) that possess close correlation with the cascade and dynamic response of mental stress are deliberately selected to build the monitoring panel (Figures 1a, S1, and Note S4). The integration of two-dimensional material modification with a regenerative strategy design, combined with optimized microfluidic structure, enables the sensing system to monitor the biomarker panel in a stable, accurate, and sustainable manner. Flexible materials and high integration endow FIRES with excellent *in situ*, real-time stress profiling capability. Building of advanced algorithms and models allows the system to generate three types of comprehensive stress indicators (SSCIs), which can be used for personalized stress assessment across diverse contexts. Validation of various types of stress by the FIRES through identification, quantification, and prognosis tracking shows the effectiveness and feasibility of stress profiling (Figure 1b). FIRES provides a promising solution for comprehensive and customized management of mental stress at the clinical scale, facilitating the essential strengthening of mental health construction.

**Figure 1:**
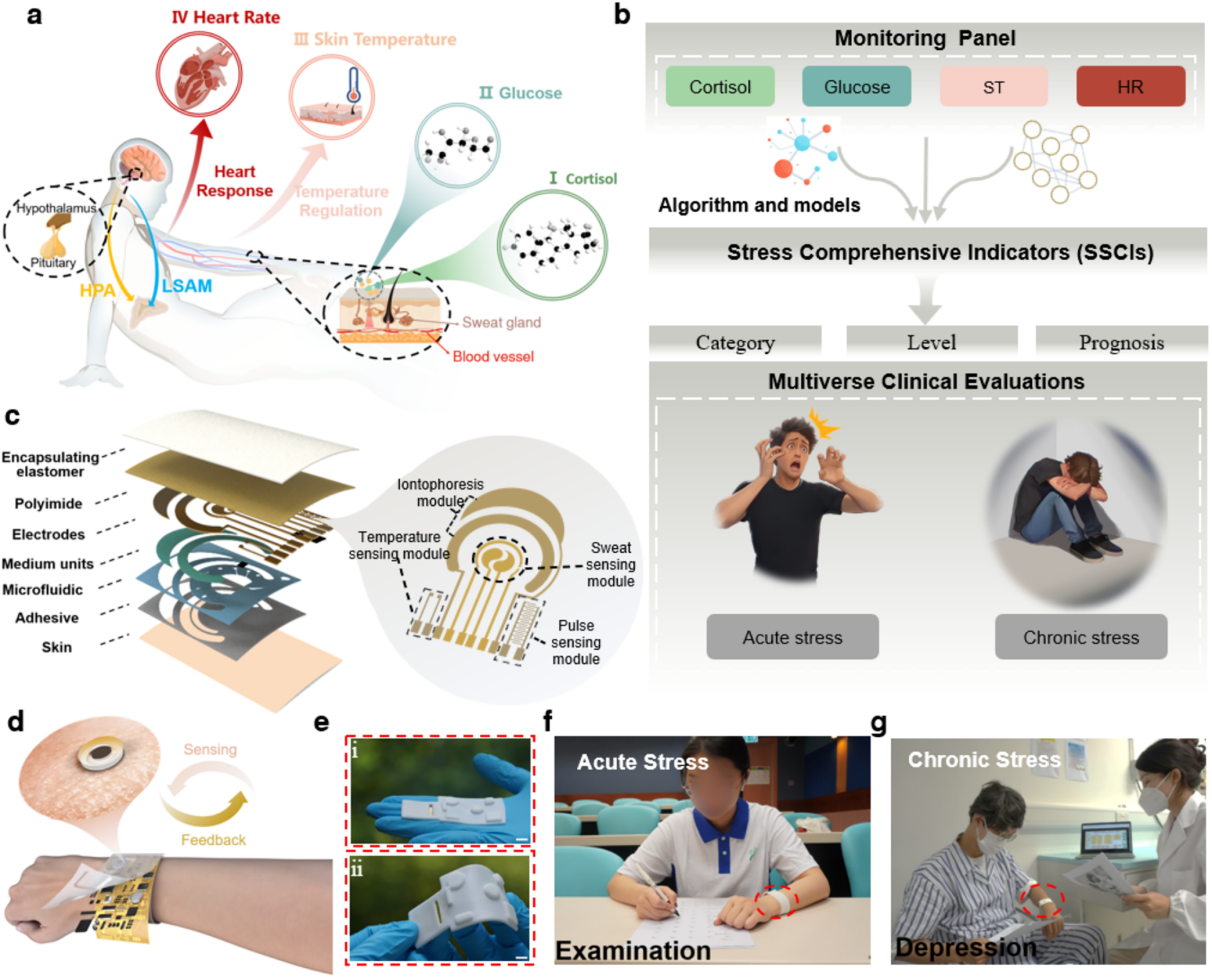
Fully integrated wearable biosystem for mental stress management. (a) The illustration of physiological responses of the human body to stress and corresponding biomarkers. The graphical elements depicting organ and tissue structures were created by figdraw.com. (b) The conceptual illustration of stress management using the wearable biosystem which contains three stages. In the first stage, four biomarkers (cortisol, glucose, skin temperature (ST) and heart rate (HR)) are selected and compose the reusable monitoring panel. In the next stage, comprehensive indicators of mental stress are generated based on the monitoring panel through different algorithm and model processing approaches. Finally, multiverse clinical evaluations are achieved for the identification, quantification and prognosis tracking in acute and chronic stress settings. (c) Exploded-view schematic illustration of the sensing system with detailed descriptions of each layer. The right panel shows the pattern of the sensor array which contains sweat sensing module, temperature sensing module, pulse sensing module and an ancillary iontophoresis module for sweat induction. (d) Schematic of the flexible printed circuit board with an electromechanical actuator embedded to realize feedback for stress warning. (e) Optical images of the FIRES at the (i) static and (ii) bending state. Scale bar: 1 cm. (f, g) Schematic of practical stress state determination using the FIRES in specific (f) acute (examination) and (g) chronic stress (depression) scenarios.

## Results and Discussion

### Development of FIRES

As shown in Figures 1c and S2, the flexible sensing patch has been designed for effective biomarker detection, which consists of a medical adhesive, a polydimethylsiloxane (PDMS) based microfluidics layer, four functional medium units (one piezoresistive sponge, one thermosensitive element, and two pieces of hydrogels), a sensor array built on a polyimide (PI) film, and an Ecoflex encapsulating layer. The chemical sensing module lays on the upper part of the integrated sensor array, consisting of two Cr/Au working electrodes (WE), an Ag/AgCl reference electrode (RE) and a Cr/Au counter electrode (CE) for detecting cortisol and glucose levels in sweat. Such substances have proven for good correlations with counterparts in the blood due to comparable diffusive transport mechanisms.^35–37^ A pair of iontophoresis electrodes surrounding the sweat sensing module enable on-demand sweat induction, which can mitigate the impact of typical perspiration factors (such as physical activity and elevated temperature) on mental stress analysis and be suitable for sedentary individuals. A Cr/Au interdigital electrode is incorporated in the lower section of the sensing patch. The piezoresistive responses of the sponge material serve as the sensing element for the HR detection.

A flexible printed circuit board (FPCB) serves as the control panel for each functional module, enabling data acquisition and wireless transmission (Figure S3). An integrated miniaturized electromechanical actuator is also designed to provide direct and controllable tactile feedback when the sensing patch recognizes a warning state indicative of mental stress (Figure 1d). The sensing patch connects with the FPCB with an adaptor, forming the whole FIRES platform. The FIRES is characterized with a thickness of <0.5 cm and a weight of ∼50 g. The thin, soft, and miniaturized characteristics allow the FIRES to be easily wrapped/mounted on the human skin, establishing a robust and stable interface for long-term operation (Figure 1e). To this end, FIRES has demonstrated remarkable efficacy in clinical applications for both AS and CS management, for instance, examination (Figure 1f) and depression development assessment (Figure 1g), respectively.

### Characterization of the Integrated Sensor Array

With the aim of detecting subtle dynamics of biomarkers in sweat, there are high demands for high sensitivity and stability of the sensors. In this view, we introduced a dense Ni-catecholate (NiCAT) layer which can be controllably *in situ* grown on the Cr/Au electrode through a simple bottom-up self-assembly approach (Figure 2a). NiCAT is a π–d conjugated two-dimensional (2D) conductive MOF which comprises metallic nodes interconnected with hexatopic triphenylene-based organic ligands.^38–40^ The Ni­(II) ions and ligands compose 2D hexagonal crystalline networks, which are further filled along the crystal’s C-axis to create a honeycomb-like porous structure. The significant orbital overlap endows NiCAT with exceptional charge transport capabilities and an abundance of active sites. Besides, the stereoscopic and porous interface structure provides a larger specific surface area and stronger binding with polymer, resulting in a more stable interface modification effect.^41,42^ The scanning electron microscopy (SEM) characterization revealed the uniform coverage of the NiCAT layer with a hexagonal prism-like morphology (Figure 2b). The characteristic lattice fringe pattern of NiCAT can be observed with a periodicity of ∼1.35 nm by the transmission electron microscopy (TEM) image (Figure 2c). Further characterizations also show the successful assembly of NiCAT on the electrode interfaces (Figure S4). The energy-dispersive spectroscopy (EDS) mapping manifested a uniform distribution of each element. The composition of functional groups and the presence of diffraction peaks can be confirmed by Fourier transform infrared spectroscopy (FTIR) and X-ray diffraction (XRD). The X-ray photoelectron spectroscopy (XPS) indicated a high level of coordination between the nickel ions and hydroxyl groups, aligning with the findings from the FTIR. Moreover, the electrode possesses a highly enhanced double-layer capacitance after NiCAT assembly (Figure S5). The electrode active surface area was calculated by cyclic voltammogram (*C–V*) scanning in ferri/ferrocyanide redox solution based on the Randles–Sevcik equation (Note S5). It was found that the Cr/Au@NiCAT electrode exhibited an enlarged *C–V* curve and a promoted active surface area (more than 2 times) compared with the bare electrode (Figure 2d).

**Figure 2:**
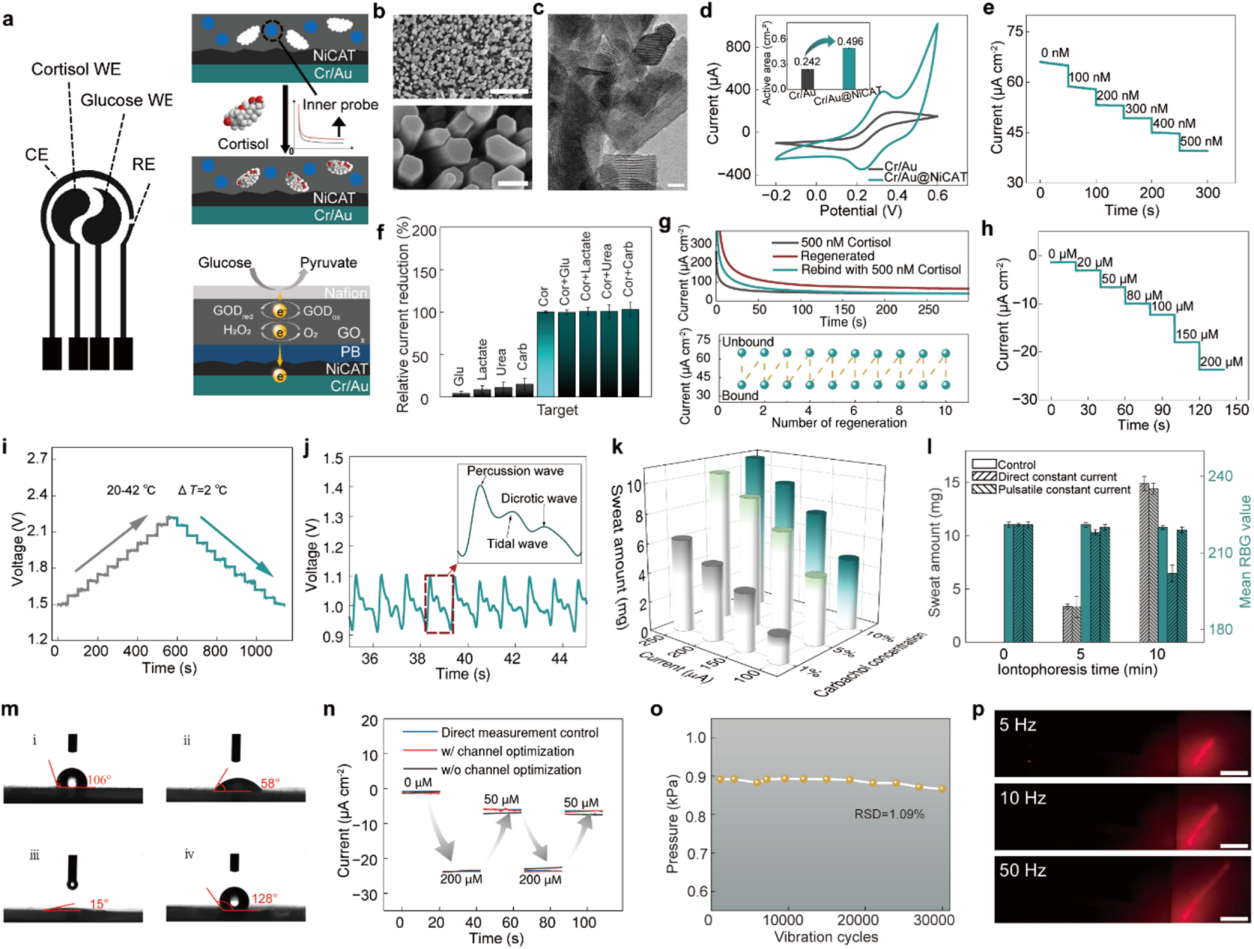
In vitro characterization of the FIRES. (a) The electrode pattern and working mechanism of sweat sensors. (b) SEM images of Cr/Au@NiCAT. Scale bar: 1 μm (Upper) and 100 nm (Lower). (c) TEM image of Cr/Au@NiCAT. Scale bar: 20 nm. (d) *C–V* plots of Cr/Au and Cr/Au@NiCAT electrode in 5 mM Fe­(CN)_6_^3–/4–^ solution. The inset shows the calculated active surface area of Cr/Au and Cr/Au@NiCAT electrode. (e) The Chronoamperometric responses of the cortisol sensors to cortisol solutions with the concentration range of 0–500 nM in PBS (1×). (f) Selectivity and anti-interference performance of the cortisol sensors. Cortisol (Cor): 500 nM; Glucose (Glu), Lactate, Urea, Carbachol (Carb): 50 μM. (g) Chronoamperometric responses of the cortisol sensors with different target bound states (Upper). Black: the sensor incubated with 500 nM cortisol; Red: the sensor after the regeneration processing; Blue: the sensor reincubated with 500 nM cortisol. The signal of cortisol sensors with bound and unbound states during different regeneration cycles (Lower). (h) Chronoamperometric responses of the glucose sensors to glucose solutions with the concentration range of 0–200 μM. (i) Current responses of the temperature sensors during the range from 20 to 42 °C. (j) The actual pulse signal detected by the pressure-sensitive electrode. The inset shows an enlarged signal waveform with distinct pulse characteristics like percussion, tidal, and dicrotic waves. (k) The influence of iontophoresis current and carbachol concentration on secreted sweat amount. (l) The sweat amount and skin conditions after 10 min iontophoresis with direct constant and pulsating constant current power source. Mean RGB values represent the average unweighted intensities of RGB channels in the arm images. (m) The contact angle of the channel interface with different processing methods. (i) pristine PDMS: 106°; (ii) plasma-treated PDMS: 58°; (iii) PDMS treated with plasma and SDS: 15°; (iv) PDMS treated with plasma and WRA: 128°. (n) Continuous detection of different glucose using the microfluidic module with and without channel interface region-specific optimization. (o) The pressure output of the electromechanical actuator within 30,000 cycles of vibrations. (p) Optical images of the light spots reflected by the electromechanical actuator with different vibration frequencies.

Among all stress-associated biomarkers, cortisol, often termed the “stress hormone”, is regarded as the most predominant indicator of mental stress dynamics. In this regard, we employed a molecularly imprinted polymer (MIP) to detect the cortisol level precisely (Figure 2a). Briefly, pyrrole was utilized as the functional monomer for electro-polymerization with cortisol as the template. After further electroelution, the templates were removed, and the MIPs with unique cavities were formed. The MIP cavities can specifically recognize the targeted cortisol with high selectivity. An inner Prussian blue redox probe was incorporated within the polymer during the electro-polymerization process, enabling *in situ* reporting of cortisol levels without additional labeling procedures or external probe circumstances. The *C–V* and electrochemical impedance spectroscopy (EIS) characterizations illustrated the preparation and detection process, demonstrating the successful probe embedding effect and MIP development (Figure S6a,b). Key parameters including the polymerization cycles and monomer/template ratio were optimized to get the best sensing response (Figure S6c,d). Moreover, it was found that only a 2 min incubation time can nearly reach the saturated sensing performance (Figure S6e). A nonimprinted polymer (NIP) was prepared by the same procedure with the absence of cortisol as the template in the polymerization step. The MIP-based sensors display a morphology that closely resembles that of NIP-based sensors, while also demonstrating a marginal enhancement in interface flatness. It was shown that the amperometric signals of the MIP-based cortisol sensor linearly increase with cortisol concentrations ranging from 0 to 500 nM, while the NIP-based control sensor has no significant response to different cortisol levels (Figures 2e and S7), demonstrating the specific recognition capability of the MIP-based sensor. The sensitivity of the MIP-based sensor are 48 nA/cm^2^/nM, with a detection limit of 0.12 nM, which is lower than the level reported in exocrine biofluids and highly competitive compared to related works.^22,43–45^ Besides, due to the unique binding effect of the MIP cavities and the target template, the cortisol sensor exhibits excellent selectivity and anti-interference capability toward interferents present in sweat (such as glucose, lactate, urea, ascorbic acid, Na^+^/k^+^, tyrosine, interleukin-6) and iontophoresis drugs (carbachol) (Figures 2f and S8). Moreover, it was found that the cortisol sensor can maintain similar signal output using different electrodes, while the further long-term test showed the ignorable relative signal variance, demonstrating considerable reproducibility and long-term stability for practical applications (Figure S9).

Real-time and in situ stress monitoring have put forward high requirements for the reusable capability of wearable sensors. It is generally believed that mediator-dependent biosensors (such as biosensors developed based on antibodies, aptamers and MIPs) are nonregenerative, meaning that once high concentrations of target substances have been identified and coupled, the biosensors fail to detect target substances with lower concentrations.^46,47^ Some methods based on interface engineering,^43,48,49^ allosteric regulation,^50,51^ external field manipulation,^52,53^ or magnetic force^54^ have been used for sensor regeneration in some cases. However, they are limited to specific targets and may affect the sensor performance. Here, we developed a simple and general strategy for cortisol regeneration by combining electric scanning and chemical elution (Note S6). The signal can be successfully recovered after the two-step regeneration operation, which indicates the release of the bound cortisol from the polymer (Figure 2g). It should be noted that the template removal step in the MIP preparation may lead to a gradual decrease in the electrode output during the repeated *C–V* scanning, which is caused by decreased conductivity of polypyrrole due to the change of crystallinity.^55,56^ The output response of the electrode reached stability after approximately 30 cycles of *C–V* scanning (Figure S10). Therefore, the number of electric elution cycles is set as 30, which ensures that the subsequent electric scanning operation in the regeneration strategy will not affect the electrical output of the cortisol electrode. Moreover, variations in scan rates during the polymerization process result in significant discrepancies in the combination effect of the monomer (pyrrole) and the template (cortisol), subsequently affecting the regeneration capability. After the signal output and regeneration performance were weighed, a scan rate of 50 mV s^–1^ was established as optimal (Figure S11). The monomer/template ratio was also optimized, while it has a modest influence on the regeneration performance (Figure S12). We evaluated the joint regeneration strategy against a singular operation strategy, revealing that the joint strategy yielded the highest signal recovery ratio (Figure S13a). Moreover, it was found that the best regeneration performance (a signal recovery rate of over 96.6% and a recovery time of less than 2 min) will be achieved when ethanol with a concentration exceeding 50% is used (Figure S13b). Importantly, repeated chemical elution operations may cause damage to the electrode interface, which in turn affects the saturation regeneration ratio. It was demonstrated that the introduction of NICAT can effectively promote electrode stability throughout successive elution cycles. This phenomenon can be attributed to the enhanced bonding strength of the interface due to the three-dimensional dense architecture of NiCAT, which leads to a robust sensor interface and satisfactory regeneration performance (Figure S13c). To sum up, through a simple liquid dripping and programmed electric scanning intervention, the sensor can exhibit commendable regeneration capabilities over more than 50 cycles, enabling the entire biosensing system to be reusable and sustainable (Figure S13d).

The enzymatic reaction of glucose oxidase was employed to specifically detect sweat glucose, with Prussian blue acting as a mediator to reduce the redox potential (Figure 2a). Generally, glucose oxidase is modified on the electrode by drop-coating and prolonged drying. This approach usually exhibits considerable modification efficiencies, but it possesses inadequate stability during the detection process involving continuous sweat rinsing, with performance deteriorating further during regeneration operations.^57^ Ergo, we utilized polydopamine to immobilize the glucose oxidase on the electrode interface via electrochemical deposition. It was found that the sensor is capable of maintaining a stable output even under repeated phosphate buffer saline (PBS) rinse at a high rinse rate of 5 mL min^–1^ (Figure S14). The glucose sensor exhibited excellent detection capability with a detection range from 0 to 200 μM and a linear response to different glucose concentrations, with a sensitivity of 0.11 μA/cm^2^/μM and a detection limit of 2.8 μM (Figure 2h). Moreover, it was demonstrated that the developed sensor possesses satisfactory reproducibility, long-term stability, and selectivity toward diverse sweat constituents and sweat gland stimulation drugs (Figure S15). The cosensing crosstalk of the sweat sensor array has also been investigated. As shown in Figure S16, both sensors exhibited specific responses and showed no significant crosstalk with respect to the other biomarker, which stems from the specific recognition of the sensing mediums (MIP and enzyme) toward the target biomarker, indicating that the sensing array possesses good detection reliability.

The inherent flexibility and ultrathin system construction render the FIRES deformable and capable of conforming to the wrapped body skin, which is beneficial for achieving a stable on-body signal collection. Evidence showed that both sweat sensors exhibit remarkably stable normalized intensities (the proportion of the detection result relative to the first detection value) during continuous bending (90°) and shaking (500 rpm) operations. Moreover, five sweat samples were collected and determined by the wearable sensor, while the commercial enzyme-linked immunosorbent assay (ELISA) kits were used as the standard control. The detection results of the wearable sensors show no significant difference from the commercial kits, demonstrating the on-body detection accuracy of the developed sweat sensor array (Figure S17). Additionally, artificial sweat spiked with different concentrations of glucose and cortisol was further analyzed, revealing that both sweat sensors demonstrated brilliant detection recovery with relative standard deviation (RSD) values less than 10% (Table S3).

A tiny NTC thermistor with fixed temperature coefficient resistances and considerable thermal sensitivity was embedded in the sensing patch to precisely detect skin temperature (Figure S18). The number of conductive carriers in semiconductors increases as the temperature rises, thus forming an ultrasensitive perception of ST dynamics via a voltage divider circuit. This temperature sensor maintained stable ladder signal responses during the heating and cooling process in the range 20–42 °C (Figure 2i). It showed a linear current output to the temperature within this temperature window, with a short response time of 1.1 s and recovery time of 2.4 s (Figure S18), which is capable of detecting the ST in a real-time manner. Additionally, the temperature sensor also serves for the calibration of the glucose sensor, as the enzymatic activity of glucose oxidase is highly affected by temperature, which can ensure a stable sensor output across varying thermal conditions (Figure S19).

An interdigital electrode in conjunction with a conductive sponge was employed and mounted on the wrist or neck to monitor the artery dynamics, which enabled detection of pulse signals and the acquisition of HR metrics. The resistance of the conductive sponge varies under applied pressure due to changes in the density of internal conductive particles (carbon nanotube/carbon black (CNT/CB)). The preparation parameters including CNT/CB amount and construction of the interdigital electrode were optimized to get the highest signal response, which ensures the capture of nuanced features in pulse signals (Figure S20). A band-pass filter was utilized to minimize the signal noise, which is beneficial for precise signal peak calculation. Moreover, the HR sensor exhibited extraordinarily stable signal responses under over 10,000 pressure cycles (Figure S21). For wearable applications, the sensor can detect the pulse signals in a real-time manner and convert them into HR values through a designed readout algorithm (Figures 2j, S22 and Note S7).

Moreover, we further investigated the sensing performance of the sensing array under repeated usage. Regeneration and sampling of cortisol and glucose sensors were performed every 15 min, with test concentrations of 500 nM cortisol and 200 μM glucose. ST and HR sensing units were continuously monitored at a sampling rate of 1 Hz, while the detection configuration was controlled to 40 °C and 10 N pressure by using a hot plate and a miniature pressure tester. As shown in Figure S23, the ST and HR sensing units exhibited good signal consistency, with signal drifts of 1.36 and 7.15 mV/h, respectively. Meanwhile, cortisol and glucose sensing units showed no significant signal variances in multiple measurements over 5 h, with RSDs of 2.56 and 5.34%, primarily attributed to the robustness of the electrode substrate. Although an increase in signal variances for the glucose sensing unit was observed after 3 h, which may stem from instability in PB and enzymes, it is still acceptable for routine testing and could be further optimized by electrode encapsulation.^30^ Collectively, the sensing units exhibit considerable signal stability under repeated usage, enabling their application in long-term and continuous monitoring.

Iontophoresis, which employs a low-intensity electric current to deliver cholinergic drugs from a hydrogel into the skin, was utilized to induce sweat. The mechanism involves cholinergic agent stimulating muscarinic 3 receptors on sweat glands to elicit sweating or triggering reflexive sweating via peripheral sudomotor axon.^58^ As shown in Figure S24, a pair of iontophoresis electrodes were designed to apply a constant current to the skin and thus for effectively and quantitively inducing sweat for a broad range of people, from active ones to sedentary ones. The iontophoresis effect of drugs on the skin was validated by using the cationic dye Rhodamine 6G (R6G) on porcine skin models. The cross-sectional histological images show that the applied constant current can effectively promote drug delivery to the subcutaneous area (Figure S24). It was found that the perspiration efficiency is enhanced with both the elevation of the applied current and the carbachol concentration (Figure 2k). Given that a transition from 5 to 10% concentration of carbachol did not yield a significant increase in perspiration efficiency, a 5% concentration of carbachol coupled with a 250 μA current was selected for further experiments, which can ensure effective sweating and produce subtle local stimulation to the human body. It is worth noting that prolonged exposure to constant current on the skin may lead to adverse effects including erythema, edema, and potential dermal rupture. Instead, pulsatile current stimulation was implemented at a frequency of 1000 Hz with a 50% duty cycle. The stimulating effects on the skin were assessed by observing the skin conditions after different modes of iontophoresis with conditions without iontophoresis processing serving as the control. Results indicated that the two modes of iontophoresis stimulation induce comparable amounts of sweat. However, the pulsatile current stimulation induced minimal alterations in skin tone, with the condition being comparable to that of the control group, suggesting a reduction in detrimental effects and localization stimulation owing to a comparatively moderated skin stimuli intervention (Figures 2l and S25).

A specially designed microfluidic module was incorporated for effective sweat control (Note S8). Three microcolumns were integrated into each inlet, which facilitated the oriented flow of generated sweat toward the sweat reservoir (Figure S26). Additionally, the inlet of channels usually requires hydrophilicity to promote the influx of sweat, but hydrophilic channels can easily retain residual sweat on their wall, thereby compromising the accuracy of subsequent sweat detection. To mitigate residual sweat within the channels, we perform region-specific hydrophilic and hydrophobic modifications on each sweat flow channel: The entrance section of each flow channel was treated for hydrophilicity using plasma and sodium dodecyl sulfate (SDS), while the end closest to the reservoir was treated with a water repellent agent (WRA) for hydrophobic modification. A buffer area of approximately 200 μm was maintained between these two sections (Figure S27). It was found that the contact angle decreased to 58° after plasma treatment, and further modified SDS reduced it to approximately 15°. After the sample was treated with WRA, the contact angle of the flow channel was restored to 128° (Figure 2m). Characterizations using blue ink within the channel indicated that the modified channel had a significant reduction in residual material (Figure S27). It should be noted that the hydrophilicity of channels treated solely with plasma will diminish over time, whereas channels treated with plasma combined with SDS can maintain hydrophilicity for a long time, rendering them suitable for extended sweat analysis (Figure S28). We further used glucose solutions of different concentrations for continuous detection. Direct in vitro measurement without the microfluidic module served as the standard control. It was found that microfluidics with optimized channels exhibited more stable signal responses and better accuracy during solution transitions (Figure 2n). Besides, the construction of microfluidics and the iontophoresis electrodes are optimized by numerical simulations to achieve optimal sweat induction and collection effects (Figures S29 and S30). The outlet geometry was intentionally designed with streamlined connections, which are beneficial for further reduction of sweat retention in the reservoir (Figure S31). This comprehensive design enables the effective and controlled collection and transportation of sweat via microfluidics for in situ analytical purposes (Figure S32).

### Characterization of the Actuator-Based Warning Module

The detrimental effects of stress on the human body usually manifest unconsciously, but sometimes even engaging in deep breath can alleviate stress burdens when they arise. Providing parallel feedback and warnings concerning the awareness of mental stress is extremely meaningful, which aids in averting the escalation of stress-related disorders and supports the scientific management of stress dynamics in daily life. In this context, we integrated a Lorentz force-based electromechanical actuator to apply tactile feedback. This miniaturized actuator features a nickel-coated neodymium magnet mounted on a thin polyimide (PI) disk with a cantilever structure, a PDMS ring, a copper coil and a polyethylene terephthalate (PET) base (Figure S33). The magnet is mobilized by alternating current, imparting pressure to the skin and extending to millimeter level depths of tissue, generating pronounced tactile feedback even in small spatial confines. It was demonstrated that the vibration of the actuator could be precisely controlled by the square wave current (Figure S33). Notably, the miniaturized actuator can keep a stable pressure feedback effect across over 30,000 vibration cycles with an RSD of 1.09% (Figure 2o). It should be noted that the actuator produces different perceptual effects when applied to different parts of the skin due to the variations in the skin modulus. For instance, the actuator positioned on the neck position generated a slightly greater pressure penetration depth compared to that on the arm position (Figure S34).

Besides, the applied pressure can be systematically modulated by the vibration frequency to generate customized tactile feedback (Figure S35). To characterize the induced subtle alterations, we use the light spot reflected by the actuator as an indicator to reflect the tactile feedback intensity. It can be found that the length of the light spot becomes more than twice as long when the vibration frequency transitions from 5 to 50 Hz, reflecting a substantial amplification of tactile feedback (Figures 2p, S35 and Supporting Movie S1).

### Physiological Characteristics of the Biomarker Panel and Its Correlation with Stress Response

The FIRES can be conformally attached to various locations on the human body, such as the wrist or neck, for the in situ monitoring of mental stress states. The detection data are transmitted in real-time to the cloud for storage and analysis via a wireless Bluetooth module. A specially designed mobile App is used to record and report mental stress results, thereby enabling personalized long-term stress management (Figures 3a, S36, S37, and Supporting Movie S2). We initially employed FIRES to monitor the biomarker panel across distinct physiological states: standing, squatting, sitting, and slow walking. As demonstrated in Figure S38, dynamic biomarker profiles could be effectively captured, processed, and visualized during these conditions. This performance stems from the system’s exceptional mechanical compliance and superior epidermal conformability. It should be noted that vigorous activities (e.g., running or jumping) may induce substantial signal artifacts or acquisition interruptions. Therefore, subjects are generally required to maintain stable physical states during signal collection, which is consistent with the inherent requirements of the stress assessment.

**Figure 3:**
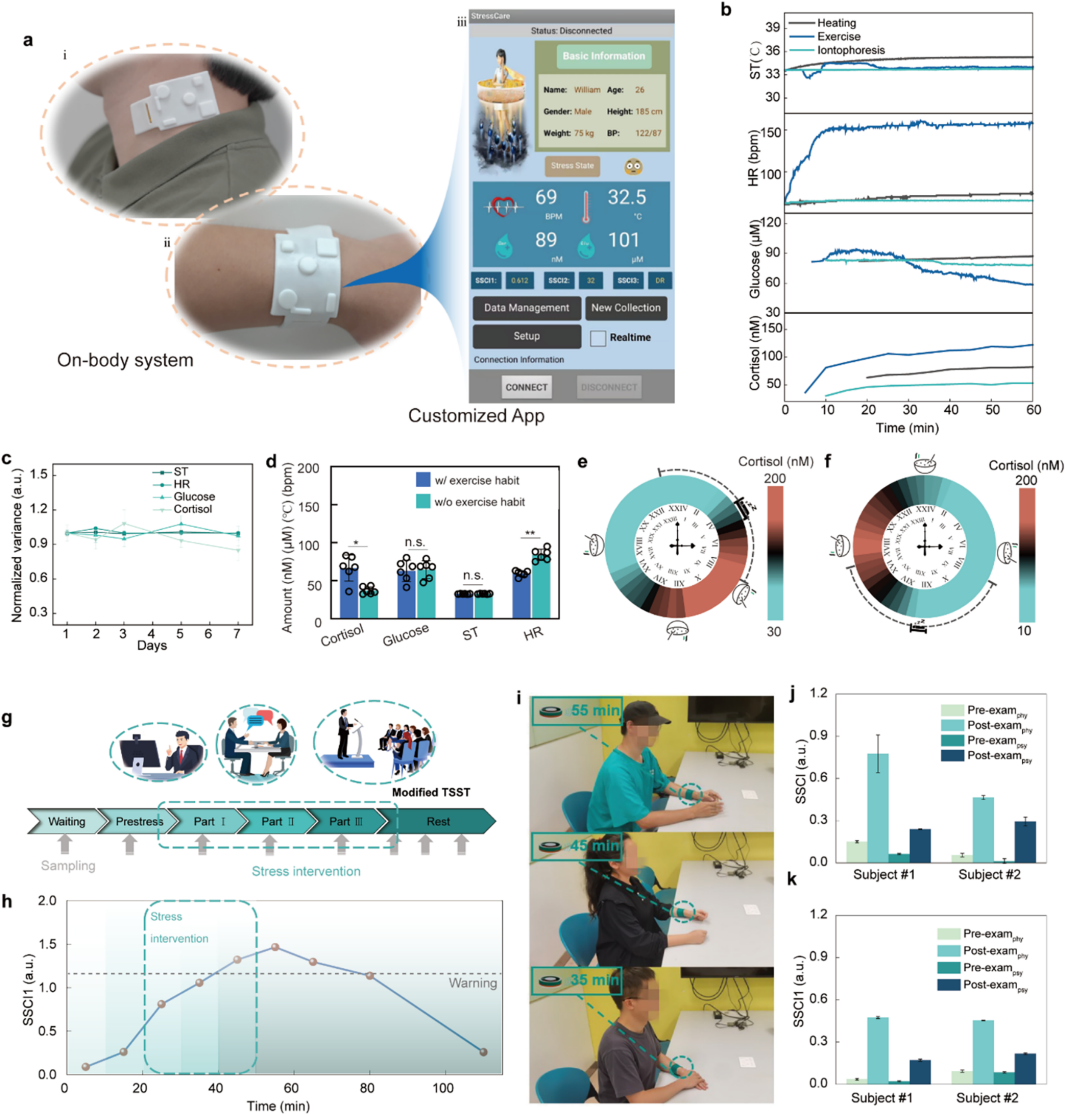
In situ characterization of physiological responses to stress stimuli using FIRES. (a) The optical images of the FIRES worn on the neck (i) and the wrist (ii), (iii) the customized App wirelessly connected by Bluetooth for health-related information management. (b) The dynamics of the biomarkers during the heating (40 °C), exercise and iontophoresis process. (c) The variances of four biomarkers within 1 week. (d) The biomarker levels among people with and without exercise habit. (e, f) The 24-h variances of sweat cortisol levels of the people with normal (e) and inverted (f) daily schedules. (g) The illustration of the modified TSST protocol. Gray arrows indicate the data collection time. The vector illustrations depicting the TSST experimental scenarios were designed by Freepik. (h) The SSCI1 indexes during the modified TSST process. (i) The optical images of three subjects undergoing the modified TSST. The subject in the top panel has already undergone a TSST before and the other two are undergoing the TSST for the first time. Insets show the testing time when the actuator is triggered. (j, k) The SSCI indexes of subjects before and after participating in different tests for the first time (j) and the second time (k).

Moreover, A FIRES-based wristband was fabricated and worn by a subject to determine the dynamic changes of biomarkers in the monitoring panel across three perspiration approaches: heating, exercise, and iontophoresis. The results indicated that all the biomarkers had significant changes during the heating and exercise process, whereas iontophoresis caused comparably negligible variations in the biomarker levels (Figure 3b). This indicates that using iontophoresis as a sweat-inducing approach could effectively minimize disruptions in mental stress state assessments. We further evaluated the concentrations of the four biomarkers in three subjects over the same time frame across a week. The results suggested that the biomarker levels in an individual show no significant variances for the same time period across consecutive days (Figures 3c and S39).

Some lifestyles and habits may affect metabolic reactions and organ functions within the body, thereby exerting a certain degree of influence on the levels of certain specific biomarkers. For instance, exercise can activate the body’s stress response and promote the secretion of cortisol to adapt to the intensity of exercise.^59^ Simultaneously it can also enhance myocardial contractility and strengthen blood circulation.^60^ Our observations indicated that individuals with long-term exercise habits have relatively high sweat cortisol levels and relatively low HR levels, while the sweat glucose level and ST have no significant variance between the two groups of people (Figure 3d), which is consistent with the conclusions of previous research reports.^61,62^ Similarly, we found that individuals with a habitual coffee intake have significantly elevated sweat cortisol levels (Figure S40), which might be attributed to the stimulating effect of caffeine in coffee on the adrenal cortex.^63,64^ Moreover, many studies have demonstrated that some metabolites like cortisol have certain circadian rhythm patterns.^22,36^ However, it is not yet clear whether the changes in these metabolite levels are principally driven by temporal factors or daily schedules. We recruited 4 volunteers, two of whom have inverted daily schedules due to occupational demands, to perform dynamic biomarker monitoring throughout the day. It should be noted that the cortisol sensor can be regenerated by injecting ethanol droplets combined with programmed electric scanning, but this active operation is difficult to implement during sleep. In this regard, we developed a small auxiliary apparatus to achieve automatic sensor regeneration during sleep during this test. This auxiliary apparatus consists of a long plastic tube and a timed pumping device. 50% ethanol allows for the formation of an ethanol-in-air structure inside the plastic tube, which is periodically pumped into microfluidic channels and combined with programmed electric scanning for sensor regeneration (Figure S41 and Supporting Movie S3). During the sleep period, each subject wore three wearable sensors on their arms to minimize the risk of a single-sensor malfunction. We observed the circadian rhythm of cortisol in each subject. It was found that the cortisol levels of subjects with a regular daily schedule peak around 8:00 pm, followed by a decline to minimal levels at night. In contrast, those with an inverted daily schedule have their cortisol levels peak in the evening and reach their lowest in the morning (Figures 3e,f and S42). The variations of ST and HR exhibited relatively stable trends, while the glucose levels are mainly affected by the diet. Notably, there are no significant differences observed in the 24-h patterns of ST, HR, and sweat glucose between these two subject groups, apart from the differing peak locations in the sweat glucose patterns (Figure S43). These results demonstrated that the circadian rhythm patterns are mainly influenced by schedule behavior rather than temporal factors.

It is essential to generate a stress comprehensive indicator (SSCI) to evaluate the level of mental stress. To this end, we first defined a comprehensive index, SSCI1 by algebraic superposition of biomarkers within the monitoring panel:

1 $$SSCI1=\sum_{i}\frac{{{biomar\ker}_{i}}_{measure}-{{biomar\ker}_{i}}_{control}}{{{biomar\ker}_{i}}_{control}}$$

The control value is taken as the value before the start of a test or the value during a stable period of the day. The relative variance of each biomarker compared to the control value is selected and substituted into the arithmetic operation, which can minimize the impact of interindividual differences. In view of this, we utilized a modified Trier Social Stress Test (TSST), a widely recognized paradigm for inducing psychological stress, to establish a mental stress scenario and monitor the SSCI1 corresponding response.^65^ The modified TSST protocol includes three stress intervention parts. Simply put, subjects are required to complete visual puzzles in part 1, mental arithmetic in part 2, and an off-script speech in part 3 (see more details in Figure 3g). We found that the SSCI1 values (reference was first recorded 30 min before the test) of the subject continuously increased during the TSST process, reaching their maximum values about 5 min after the stress intervention in the third part, gradually decreasing during rest, and returning to the level before the test about 1 h after rest (Figure 3h). The biomarkers in the monitoring panel showed a similar trend with the SSCI1, while the cortisol exhibits the largest variance during the TSST process. Moreover, the response of glucose levels during the stress process is notably more delayed compared with other biomarkers, which can assist in temporal analysis of stress states (Figure S44a). We subjectively set 80% of the maximum SSCI1 value during this TSST test as the warning value to trigger feedback from the actuator and investigate the differences in response among different subjects. The subject was asked to participate in the TSST test for the second time, and two other subjects also participated. We found that the SSCI1 and biomarker panel responses exhibited comparable trends among subjects during the TSST (Figures S44 and S45). In addition, the SSCI1 values during the second TSST participation showed a significant decrease compared with the first time. Moreover, for the subject who participated in the TSST experiment twice, the actuator-based warning module triggered at the 55th minute, which was later than that for the other two subjects experiencing the task for the first time (Figure 3i). This proves that the subject has a certain resilience to repeated stress-induced experiments. It should be noted that this warning value is merely set to compare intersubject variations in stress responses under a unified warning criterion. The accurate and reliable warning value should be set based on empirical validation and repeat experimental investigations.

We further tested the other intervention actions (specifically, competitive game (CG), action movie (AM), physical exam, psychological exam, and speech) and observed the SSCI1 responses. It was found that these intervention actions can induce a high SSCI1 response compared to the rest control (Figure S46). Moreover, the physical exam was found to induce a greater SSCI1 increase compared to that of psychological exams. The SSCI1 response in both tests diminished from the initial exam (Figure 3j) to the second administration (Figure 3k), which is consistent with the results exhibited in the TSST settings.

### Clinical Characterization and Customized Management of Acute Stress

AS is typically featured in short duration, intensive reaction, and unpredictable causation. Initially, AS evolved as a survival mechanism beneficial to the organism’s body, as it can boost energy supply and activate neural responses to quickly cope with potential threats, which is called as “fight-or-flight” response. Nevertheless, the body may overreact to stressors, leading to excessive activation of relevant response pathways in the body. Therefore, it poses profound significance for the effective identification and tracking of AS responses. The above-defined SSCI1 is suitable for roughly assessing the changes in stress states before and after exposure to certain stressors, but it is challenging to conduct accurate real-time analysis of unpredictable AS dynamics. A serious concern is that certain biomarkers (cortisol and glucose) have pronounced time-dependent variations, which lead to a non-negligible intraindividual variance that complicates the assessment of AS states. In this regard, we proposed a universal approach to generate the comprehensive index (SSCI2) for the customized assessment of specific AS settings:

2 $$\mathrm{SSCI}2=\sum_{i}\omega_{i}\frac{{{(\mathrm{biomarker}}_{i})}_{\mathrm{measure}\&t}-{{(\mathrm{biomarker}}_{i})}_{\mathrm{baseline}\&t}}{{{(\mathrm{biomarker}}_{i})}_{\mathrm{baseline}\&t}}$$

Specifically, we aim to establish the baseline value of a biomarker at a designated time point (biomarker_baseline&*t*_), and subsequently assess the relative change in the observed value (biomarker_measure&*t*_) in relation to the baseline value, i.e., the temporally coupled biomarker as an evaluation metric (Note S9). Next, for specific AS settings, we generated the impact weights (ω) of each evaluation metric on the stress state through multiple linear regression, contributing to the ultimate SSCI2 output (Figure 4a, Note S10). To minimize the interference of intraindividual variance, specific mathematical models were selected based on preliminary experiments to fit the cortisol and glucose data, which exhibit distinct circadian rhythms and diet-related behaviors, respectively (Figures S47 and S48). These data were processed and fitted considering their temporal evolutionary patterns throughout the day (8:00–22:00) to derive the dynamic biomarker baseline value, which can effectively facilitate the reduction of the data variance to a certain extent (Figure S48f). It should be noted that, given that ST and HR do not show a clear pattern of change throughout the day under normal circumstances (Figure S43), a constant baseline value (selected as the measured value at 8:00 and obtained in advance) is used for calculating their evaluation metrics of SSCI2.

**Figure 4:**
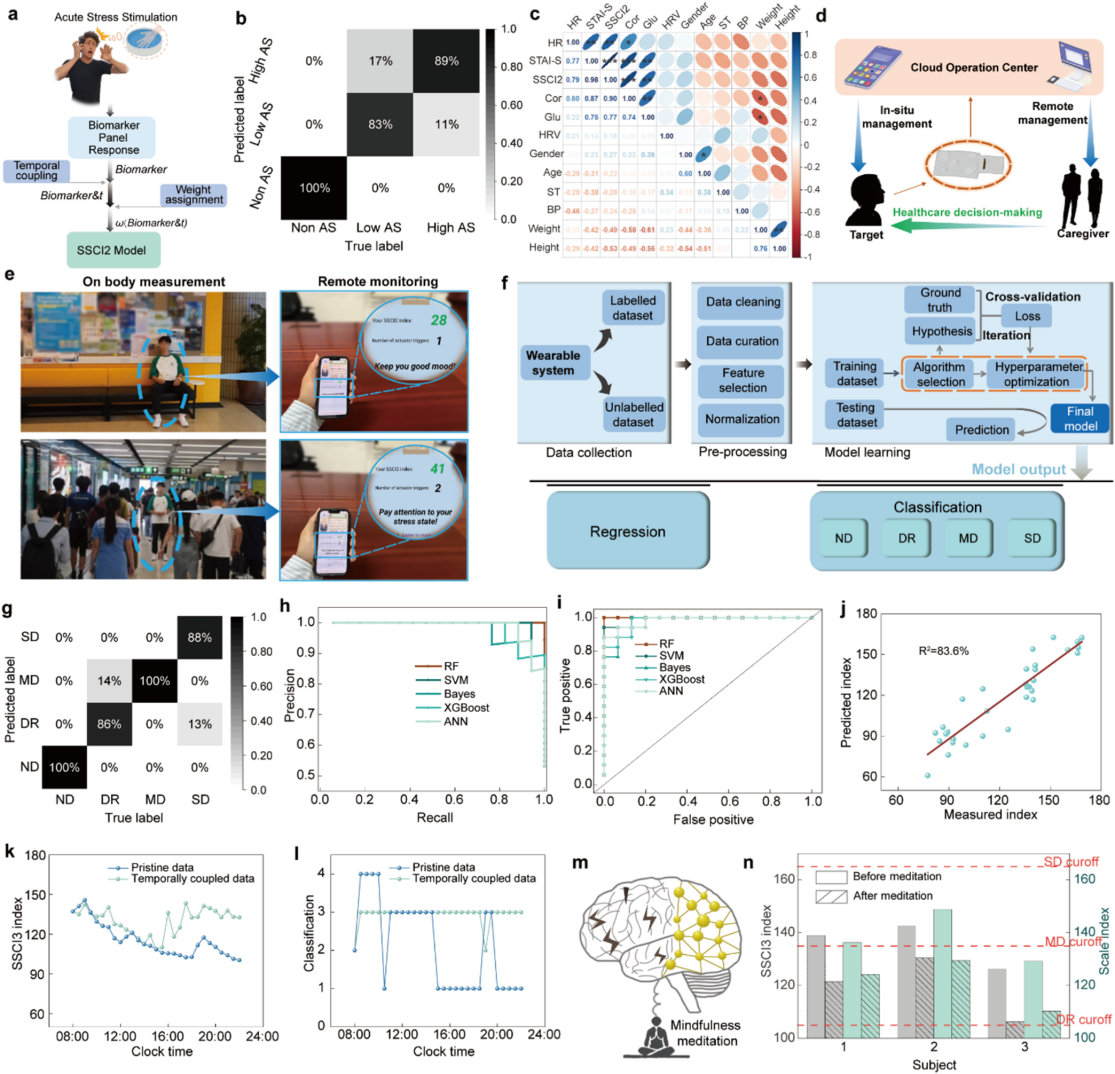
Clinical evaluation of AS and CS using FIRES. (a) Schematic illustration of the SSCI2 model establishment for AS profiling. CPT is illustrated as an example of AS stimulation. CPT illustration image is created by figdraw.com. (b) Confusion matrix of model predictions for AS state identification. (c) Correlation map of multiple detection biomarkers and evaluating results. ***: *p* < 0.001; **: *p* < 0.01; *: *p* < 0.05. (d) Conceptual diagram of the integrated wearable system for efficient AS management. (e) The illustration of a patient with SAD staying in a quiet and noisy environment. The corresponding remote monitoring App UI shows the SSCI2 indexes of AS level. (f) The machine-learning model framework for evaluating depressed patients. (g) Confusion matrix of model predictions for different depression state identification. (h) The PR curves of depression discrimination using different algorithms. (i) The ROC curves of depression discrimination using different algorithms. (j) The correlation between the indexes predicted by the model and the indexes measured by the scales. (k) Continuous SSCI3 index tracking of a depression patient over the daytime using the data with and without temporal processing. (l) Continuous depression type analysis of a depression patient over the daytime using the data with and without temporal processing. (m) The concept illustration of mindfulness meditation. (n) The SSCI3 indexes and the scale indexes of three depression patients before and after meditation intervention.

The cold-pressor test (CPT), recognized as a standard AS induction method, was employed to evaluate the performance of the AS assessment. A total of 12 subjects were instructed to immerse their hands in ice water for 1 min, while the other hand was equipped with the FIRES to monitor the dynamics of the biomarker panel before and after the test. The condition of immersing hands in room temperature water (25 °C) was used as the control group. Concurrently, the State-Trait Anxiety Inventory (STAI)-S scale was used as a standard evaluation result for subjects’ stress states.^66^ It was found that the STAI-S scores had a significant promotion in the CPT (*p* < 0.05), in contrast to no notable changes in the control test (Figure S49), indicating the stress induction effect of the CPT. The biomarkers in the monitoring panel also showed a distinct elevation after CPT intervention, except ST, which might be due to the rapid modulation effect of the human thermoregulatory system (Figure S50). In view of this, we did not include ST results in the calculation of the SSCI2 index for the CPT setting. The impact weights of the remaining three biomarkers were derived based on the test data of the 12 subjects and the STAI-S results using multiple linear regression, and the SSCI2 index was ultimately generated. We used evaluation scores of 34 and 44 as critical points to classify AS states into three categories: Non-AS, Low AS, and High AS. It was found that the SSCI2 shows excellent performance for AS state identification with a total 92.9% accuracy among 28 subjects (Figure 4b). The correlation analysis was performed using the test results and other physiological indicators such as mean blood pressure. It was found that the SSCI2 values are highly correlated with the STAI-S results, while no significant correlation was observed with other indicators aside from cortisol, glucose, and HR (Figure 4c).

The occurrence and severity of AS are usually beyond self-awareness. FIRES presents an encouraging opportunity for the development of personalized or remote management approaches through cloud-based information operations, facilitating timely and effective decision-making when required (Figure 4d). As a proof of concept, a patient with social anxiety disorder (SAD) was instructed to wear FIRES while engaging in both tranquil and noisy environments. The sensors collected monitored data and uploaded it in real-time to a cloud processing platform, which then synchronized the data results to the caregiver’s mobile phone at home. The patient exhibited a significantly elevated SSCI2 value upon transitioning from a tranquil environment to a noisy setting. This data was successfully relayed to remote caregivers, which can assist caregivers in tracking the patient’s condition without the need for close physical presence (Figure 4e). It should be noted that the impact weights used for calculating the SSCI2 values here are obtained from previous CPT experiments. Although the body’s responses to different acute stressors may vary, there are certain commonalities in the manifestations of the AS physiological pathway responses.^14^ Therefore, the impact weights analyzed for particular stress contexts can also be applicable to utilize in other AS contexts for qualitative or semiquantitative analysis, while targeted training will enhance the accuracy in SSCI2 model building and other function implementation like state warning.

### Machine Learning-Based Chronic Stress Management

CS generally manifests as a low-level but consistently detrimental influence on the human body, directly undermining mental health and potentially triggering a range of physiological ailments, such as cardiovascular disorders and immune system diseases.^18,67^ The intricacies of CS are particularly notable concerning its etiology, mechanisms, and pathological manifestations. It often encompasses an intricate interplay of various emotions such as anxiety, panic, and sadness, making the definition and precise analysis of CS particularly challenging. Conventional laboratory-based clinical analysis equipment shows limited effectiveness in assessing CS. Here, the advent of the customizable wearable device highlights the advantage of collecting large-scale data generated from a diverse pool of individuals. Their combination with continuously evolving assessment models for personalized identification and quantification of CS appears to offer a promising avenue for the effective management of CS (Figure S51).

The FIRES allows noninvasive monitoring of the predominant biomarker panel in a real-time and in situ manner, facilitating comprehensive analysis of CS dynamics. Here, we built a machine-learning (ML)-enabled method for the assessment of the typical CS-induced disorder—depression. A total of 23 depression patients and 26 healthy controls were recruited, while the inclusion criteria strictly adhered to clinical diagnostic standards. The classification and regression labels are assigned based on the combination of three authoritative depression testing scales: Symptom Checklist 90 (SCL-90), Self-rating Depression Scale (SDS), and Self-Rating Anxiety Scale (SAS) (Note S11). The biomarker panel was collected through the FIRES worn on the hand and used as input features for the model along with conventional demographical features such as gender, age, height, weight, and mean blood pressure. Considering the inherent variations of cortisol and glucose throughout the day, their data from each test point were retroactively adjusted to correspond to the benchmark time point (8:00 for cortisol and glucose), based on the previously established fitting functions before input into the model (Notes S9 and S10). Each subject contributed test data at three distinct times throughout the day. The comprehensive index (SSCI3) was generated from the classification or regression output of the model (Figure 4f):

3 $$\mathrm{SSCI}3={\mathrm{model}}_{\mathrm{classification}\;\mathrm{or}\;\mathrm{regression}}\left(\mathrm{Cor},\;\mathrm{glu},\;\mathrm{BT},\;\mathrm{HR},\;d\mathrm{emographical}\;\mathrm{features}\right)$$

Random forest (RF), Support Vector Machine (SVM), Bayes, XGBoost and Artificial Neural Network (ANN) were used as the classification algorithms, and the linear regression algorithm was selected as the regression algorithm of the model. All of the sample data were categorized into four groups based on scale scores: Non depression people (ND), people with depression risk (DR), mild depression patients,(MD) and severe depression patients (SD). The principal components analysis (PCA) illustrated the clustering of the samples from the same group (Figure S52). It was found that the RF algorithm outperforms other algorithms with an outstanding accuracy of 93.3% (Figures 4g and S53). We combined MD and SD into the patient group, and ND and DR into the healthy control group for binary qualitative diagnosis of depression. The precision-recall (PR) plot and receiver operating characteristic (ROC) plot indicated significant performance metrics across the five algorithms, with RF achieving an area under the curve (AUC) of 1 in both PR and ROC analyses (Figure 4h,i). The quantitative SSCI3 index can be gained by the model by using the linear regression algorithm. The true labels were identified by the weighted addition of the three scale scores (Note S11). It was found that there is a robust linear correlation between the SSCI3 regression indexes and true label scores (Figure 4j).

To investigate the effect of temporal processing of cortisol and glucose data on the SSCI3 merits, the continuous tracking data of a depression patient and a healthy control over the daytime (8:00–22:00) with and without temporal processing were imported to the built machine-learning model for SSCI3 analysis. The results demonstrated that the predicted SSCI3 indexes with temporally coupled data showed smaller variations (RSD: 7.6%) compared to those with pristine data (RSD: 11.2%) (Figure 4k). The depression types were classified as MD using the data with temporal processing, with the exception of results only at two time points (Figure 4l). In contrast, the model classification results are significantly distinct throughout the entire daytime. These findings are consistent with the results of the healthy control (Figure S54). The SSCI3 index outputted by the unprocessed cortisol and glucose data showed significant data dynamic characteristics, and the classification discrimination results fluctuated. After data temporal processing, the model enables a more stable and consistent output for the subjects during an extended time period.

Mindfulness meditation is an effective method for promoting and treating mental health, facilitating individuals’ ability to manage adverse emotional states, enhance self-awareness, and refine self-regulation skills, which collectively contribute to the alleviation of depressive symptoms (Figure 4m).^68,69^ Three depression patients were instructed to undergo a week of meditation intervention, during which relevant physiological data were collected, and scale assessments were conducted both before and after the intervention. The results indicated a significant decrease in the SSCI3 indexes and scale results for all three patients after 1 week of treatment, with a consistent decline observed across both methods of measurement (Figure 4n). This suggests that the SSCI3 index produced through the FIRES in conjunction with ML models may serve as an effective alternative to conventional assessment techniques for clinically managing the stress development of patients with mental health disorders.

Moreover, to investigate the applicability of the established stress assessment model in other chronic stress scenarios, we employed the FIRES and SSCI3 models to monitor the dynamic levels of stress during the fat-loss intervention phase. As shown in Figure S55, the volunteer was asked to engage in a two-week fat-loss training regimen, which included one h of strength training and half an hour of aerobic training each day. The caloric intake during the training period was strictly regulated. Day 10 was designated as a break day. (See detailed protocols in the Methods section). Biomarker data and demographical information were recorded daily at 08:00 and 20:00. The results found that the HR, sweat glucose and cortisol level showed distinct fluctuations, especially in the first weeks. Cortisol levels continuously increased before the break day, followed by a slight decrease during the second training period. A significant reduction in body weight was observed during the initial week; however, the rate of decline gradually diminished until a plateau was reached. After the break day, there was a slight increase in weight, but throughout the second phase of the training cycle, a gradual decrease was noted. Correspondingly, the SSCI3 index exhibited a holistically increased trend before the break day, demonstrating an increased stress level induced by training and diet control. The slight reduction in the SSCI3 index was observed after the break day, which is similar to the cortisol dynamics. The evaluated stress levels in the evening were higher than those in the morning, which might be attributed to the mental load induced by training and the measured weight data. In summary, the SSCI3 model built from the specific chronic stress settings demonstrated potential applicability for stress assessment in other stress scenarios, and similar physiological regulatory pathways under the same type of stress stimuli may primarily account for this phenomenon.

## Conclusions

We report a strategy with the collection of advances in physiological fingerprint profiling, integrated construction design, signal processing, and software engineering for comprehensive mental stress management. A flexible wearable platform, FIRES, was developed based on the combination of a physiochemical biosensor array and an electromechanical actuator warning module for stress monitoring and feedback. Four biomarkers selected according to the thorough investigation of the stress-induced physiological pathways can effectively reflect the metabolic and neurological dynamics and compose the stress monitoring panel. Among these, cortisol plays a predominant role in stress state profiling, while its extended application in wearable monitoring systems is hindered due to the difficulty in repeated recognition using unreplaced electrodes. In this regard, we proposed an electrode engineering strategy combining electric scanning and chemical elution to achieve the regeneration of cortisol sensors, rendering the entire monitoring system reusable to ensure sustainable applications. The intrinsic physiological characteristics of the biomarker panel were investigated along with their correlations to mental stress dynamics. Standard stress induction protocols and daily stress-related events were utilized and have demonstrated the indicative effect of the biomarker panel in mental stress situations.

For qualitative and quantitative assessment of mental stress levels, we generated three SSCI indexes based on the biomarker panel and different algorithm strategies, which are capable of application with different clinical demands. Based on these, we offer proposals for accurate, efficient, and customized management of AS and CS based on the integration of wearable interface, cloud data processing, and intelligent evolving models. For proof of concept, we investigated the performance of FIRES and the developed SSCI indexes in two clinical settings: SAD and depression. It was found that the stress dynamics of the patient with SAD can be effectively tracked and provide timely feedback, ensuring timely insights for caregivers and facilitating appropriate decision-making when needed. The mental stress state of depression patients can be successfully recognized and quantified, with minimal temporal interference from the intravariances. Further, we demonstrated the comparable efficacy of the wearable interface against clinical scale survey methods in tracking the progression of depression and evaluating the performance of therapy plans. Moreover, we envision that our FIRES and the corresponding proposal hold promising potential for clinical application in other physical and psychological healthcare contexts.

Collectively, we designed a fully integrated biosystem considering functional materials, sensing strategies, system integration, and algorithm processing, which emphasizes the effectiveness of intelligent wearable interfaces on mental stress management. This biosystem incorporated multiple practical factors and key performance considerations and established, for the first time to our knowledge, the effectiveness of wearable devices in real-world clinical stress analysis. Compared to existing approaches reported in the literature, it exhibits improved robustness and practicality (Table S6). However, accurate detection outcomes are consistently generated with the prerequisites of a thorough understanding of physiological background theories, cutting-edge algorithm design, extensive data models, and rigorous clinical validation on a large scale. We have established a preliminary demonstration of integrated wearable systems for mental stress management on the clinical scale, but there is still a significant journey ahead to achieve more generalized and reliable clinical implementations. The next generation of the system should lie on the following aspects: (1) More comprehensive and reasonable monitoring panel which might incorporate more valuable parameters like HRV and EDA, paired with accurate and efficient sensing strategies; (2) Systematic system integration enabling automatic, stable, and prolonged usage, such as introducing burst valves or magnetic valves^70,71^ into the microfluidic module to realize programmable delivery and fixed point storage of external elution solution; (3) Optimized algorithm development for sweat response latency reduction by time lag calibration,^58^ as well as interference elimination and customized application; (4) Advanced models based on large-scale databases and extensive clinical validations.

## Methods

### Materials and Reagents

Photoresists (AZ P4620 and SU-8 2050) and developers were purchased from AZ Electronic Materials and Microchem. Chitosan, dopamine, acetic acid, potassium ferrocyanide (K_3_[Fe­(CN)_6_]), ferric chloride (FeCl_3_), potassium chloride (KCl), potassium iodide (KI), sodium chloride (NaCl), Carbachol, iodide, hydrochloric acid (HCl), isopropyl alcohol (IPA), glucose, and pyrrole were purchased from Sigma-Aldrich. Single-walled carbon nanotube (SWCNT) was purchased from Aladdin Reagent. Glucose oxidase (GOx), Nafion, cortisol, ascorbic acid, tyrosine, IL-6, phosphate buffer solution (PBS 1× and 10×), glucose assay kits, and cortisol assay kits were purchased from Sangon Biotech. Thermistors (NTC 0603) were purchased from Mouser Electronics. The Ag/AgCl ink was purchased from the Ju-long Elec Tech Co., Ltd. Polydimethylsiloxane (PDMS, Sylgard 184 Kit) was obtained from Dow Corning Co. Ltd. Water was deionized before use (18.25 MΩ cm). All chemicals were of reagent grade or higher, and all materials were used as received.

### Fabrication of the FIRES Platform

#### Multiplex Sensor Module

The sensor array substrate was fabricated by photolithography on a PI film (100 μm). Briefly, The PI film was processed by ultraviolet (UV) light and then cleaned with ethanol, acetone, and deionized water accordingly. Then a layer of Cr/Au (10/100 nm) was deposited on the PI film by electron beam evaporation. To pattern the Cr/Au layer, a photoresist layer (AZ 4620) was first deposited by spin-coating at 3000 rpm for 30 s and baking at 110 °C for 5 min. Then the film was exposed to UV light with a special design mask for 45 s and developed for 1 min (AZ 400 K). Abundant Au and Cr were etched by KI/I solution and ceric ammonium nitrate/nitric acid solution, respectively. Finally, the film was washed with acetone to remove the residual photoresist. The sweat-sensing WEs were first assembled with a layer of NiCAT by hydrothermal synthesis and calcination. Then they were modified with specific functional layers for chemical detection. A miniaturized NTC thermistor was installed on the film bridges of two Cr/Au pins by soldering. A commercial high-density sponge was cut by laser cutting into blocks of specific sizes. Then the sponge was immersed in a CNT/carbon black/PVA mixture for 30 min and dried overnight to obtain the piezoresistive sponge. The sponge was then attached to the patterned interdigital electrode with ultrathin double-sided tape. An Ecoflex elastomer was developed by mixing parts A and B with a weight ratio of 1:1. The obtained mixture was poured into a mold and then covered in the whole patch for encapsulation.

#### Iontophoresis Module

The drug-loaded hydrogels were prepared based on 3% (w/w) agarose hydrogels which were made by heating to boiling in an oven and then cooling down. 5% (w/w) carbachol and NaCl were added to the agarose mixture and stirred continuously when the mixture temperature dropped to about 140 °C, serving as the anodic and cathodic hydrogels for iontophoresis, respectively. The mixture was then poured into a patterned mold for curing and taking shape. The cured hydrogel (around 200 μm thickness) is stored at −20 °C for subsequent use.

#### Microfluidic Module

The microfluidic module for sweat detection was fabricated by photolithography and replica molding. For preparing the mold of the microfluidic module, SU-8 photoresists (2050) were first spin-coated on a silicon wafer at 1200 rpm for 60 s to form a thickness of about 150 μm and then soft-baked at 65 °C for 5 min and 95 °C for 20 min. After exposure to UV light with a mask, the wafer was postbaked at 65 °C for 5 min and 95 °C for 10 min, then it was immersed in the developer solution and shaken evenly for 10 min. Fresh IPA solution and deionized water were used to alternately clean the wafer. Finally, the wafer was hard-baked on a hot plate at 250 °C for 30 min. Next, a layer of Trichloro (1H,1H,2H,2H-perfuorooctyl) silane was spin-coated on the mold to facilitate the release of PDMS. PDMS mixture (elastomer: curing agent, 10:1) was degassed for 30 min and then poured into the prepared mold. After baking at 70 °C for 30 min, the PDMS microfluidic layer was peeled from the wafer and cut to a shape of approximately 18 × 20 mm^2^. The inlets and outlets of the microfluidic layer were obtained by punching holes of 0.9 and 1.8 mm diameter using specific syringe needles. For channel interface modification, the microfluidic was first treated with oxygen plasma for 30 s. A PDMS cover (elastomer: curing agent, 20:1) was made using the same steps as the microfluidic layer. The cover was attached to the microfluidic layer, and the microcolumn of the cover can divide each channel of the microfluidic layer into two parts. Two holes were drilled near the microcolumns on the cover for injecting decorative materials into the two parts of the microfluidic channels. The part of the channel near the inlet was treated with SDS solution for 3 min at 90 °C for hydrophilic modification, and the part near the reservoir was treated with a WRA (SINO-100) for 5 min for hydrophobic modification. Finally, the microfluidic layer was peeled off from the cover layer and then bonded to the sweat sensing layer for a subsequent sweat detection operation.

#### Actuator-Based Warning Module

The construction of the electromechanical actuator (shown in Figure S33) refers to previous reports.^72,73^ The top PI supporting layer (0.2 mm) was laser cut into a circular disc (diameter: 11 mm) with a cantilever-like platform. A permanent magnet (diameter: 5 mm; thickness: 0.5 mm) was attached to the disc with glue. A ring (outer diameter: 11 mm, inner diameter: 8 mm, thickness: 1 mm) was 3D printed using UV-curable resin and then glued to the disc layer. The space inside the ring allowed the magnet to vibrate freely. The Cu coil (outer diameter: 11 mm, inter diameter: 2 mm, thickness: 0.25 mm) was prepared by winding 50 turns of Cu wire (diameter: 0.05 mm) and then connected to the other side of the ring. After all of the components were assembled, the electromechanical actuator was obtained with a diameter of 11 mm and thickness of 1.5 mm for warning feedback.

### Electric Circuit Design

For operating FIRES, a low-power microcontroller unit (LPMCU) (STM32L053C8U6, STMicroelectronics Inc.) is adopted to manage the whole system, including biosensor arrays (glucose, cortisol, ST, and HR), iontophoresis, a warning actuator, and wireless communication. A 4.2 V lithium-ion battery is utilized, while the electrical power is adjusted to 3.3 V by LDO (LP3990MFX-3.3/NOPB, Texas Instruments Inc.) to provide stable electrical power for the system. A series of operational amplifier (Op-Amp) circuits using four Op-Amps (LT1462CS8#PBF, Analog Devices, Inc.) including a transimpedance amplifier (TIA) circuit is applied for glucose signal processing. A programmable analog front-end (AFE) module (LMP91000SD/NOPB, Texas Instruments Inc.) is connected to the LPMCU for interintegrated circuit (I2C) communication of the cortisol module. Voltage divider circuits are applied for the temperature and strain module, while a surface-mounted device (SMD) type of NTC thermistor is utilized as thermosensitive media. Multiple filters and smooth techniques are applied to reduce the motion artifacts of the signals. The iontophoresis is mainly managed by a voltage-controlled current source circuit that is composed of an Op-Amp (LT1784IS5#TRPBF, Analog Devices, Inc.) and an *N*-type MOSFET (FDT86246L, ON Semiconductor Corp). To provide a sufficiently high reference voltage to the Op-Amp and current pathway, 3.3 V from the LDO is boosted to 15 V by a DC/DC converter (LT8364IDE#PBF, Analog Devices, Inc.). Then, the frequency and magnitude of the current flow are regulated by the digital-to-analog converting (DAC) and pulse width modulation (PWM) functions of the LPMCU. The processed biosensor data are wirelessly transmitted to an external device through a Bluetooth module (WH-BLE103, Jinan USR IOT Technology Limited) for displaying them on a customized graphical user interface (GUI). The data is updated every two min, while the iontophoresis can also be manually controlled by the external device wireless at the same time.

### Preparation of Sweat Sensors

#### MOF Modification

The NiCAT film was self-assembled on the electrode substrate by a simple bottom-up protocol which refers to previous reports.^38,39^ Briefly, 20 mg of Ni­(OAc)_2_·4H_2_O and 14 mg of HHTP were dissolved in 10 mL of mixture solution of deionized water and 1-propanol (v/v, 1:1) and then treated with ultrasound for 30 min. The mixture was filtered by a syringe filter to get the pure precursor solution. Next, the obtained solution was dropped on the electrode and then placed on a raised platform in an autoclave. The bottom of the autoclave was filled with a mixture solution of deionized water and 1-propanol (v/v, 1:1). The autoclave was sent to an oven and heated at 85 °C for 2 h. A dense deep blue MOF film grew on the surface of the electrode after heating. The electrode was cleaned with deionized water, ethanol and acetone. Then it was placed in a tube furnace and calcined at 100 °C for 5 h. Finally, the electrode was cooled overnight at room temperature and stored at 4 °C for subsequent use.

#### Glucose Sensor

A Prussian blue layer was assembled on the electrode using the *C–V* electrodeposition method by sweeping from 0 to 0.5 V (vs reference electrode) for 4 cycles at a scan rate of 0.02 V s^–1^ in a solution containing 2.5 mM FeCl_3_, 2.5 mM K_3_Fe­(CN)_6_, 100 mM KCl, and 100 mM HCl. Two mg mL^–1^ SWCNT was added into 1 wt % chitosan solution and then drop-cast on the electrode. After drying for 4 h, GOx was immobilized on the electrode by immersing the electrode into a solution of 20 mg mL^–1^ GOx and 2 mg mL^–1^ dopamine with *C–V* scanning from −0.5 to 0.5 V (vs reference electrode) for 35 cycles at a scan rate of 0.05 V s^–1^. Next, 0.5 wt % Nafion was drop-cast on the electrode and dried overnight at room temperature. The electrochemical deposition was operated using the three-electrode system with an Ag/AgCl electrode as the reference electrode and a Pt wire as the counter electrode. Electrodes were rinsed with deionized water and ethanol after each electrodeposition step to remove residual reaction solution and stored at 4 °C.

#### Cortisol Sensor

The preparation of MIP-based cortisol sensors involves two steps, polymerization and elution. First, a polypyrrole film along with the inner probe was deposited on the electrode by *C–V* scanning from −0.2 to 0.9 V (vs reference electrode) for 10 cycles at a scan rate of 0.05 V s^–1^ in a solution containing 4 mM cortisol, 20 mM pyrrole, 5 mM FeCl_3_, 5 mM K_3_Fe­(CN)_6_ and 100 mM HCl. Then the electrode was electroeluted in the PBS solution by *C–V* from −0.2 to 0.8 V (vs reference electrode) at a scan rate of 0.05 V s^–1^ until the *C–V* curve no longer changed (approximately 30 cycles). The obtained electrodes were cleaned with deionized water and stored at 4 °C for subsequent use.

### Regeneration of Cortisol Sensors

The regeneration of cortisol sensors was based on sufficient electric elution during the MIP preparation steps. Two procedures involving electrical scanning and chemical elution were combined to realize the recondition of the MIP (Note S6). Two cycles of electrical scanning from −0.2 to 0.8 V (vs reference electrode) at a scan rate of 0.05 V s^–1^ were utilized to weaken the binding effect between MIP and the targeted cortisol molecules. Ethanol was utilized to elute cortisol from the MIP cavities due to the differences in cortisol solubility. 50% ethanol was used, considering the elution effect and the feasibility of forming an ethanol-in-air structure. In practical applications, electrical scanning was executed by a programmable command driven by the FPCB and can be performed in PBS or raw sweat. Subsequently, a droplet of an ethanol solution (50%, v/v) was delivered to fill the detection chamber from the microfluidic outlet or inlet. The delivery operation was achieved using a tiny micropipet or syringe (1 mL). For sustainable nighttime monitoring of biomarkers, we developed a small auxiliary apparatus controlled by a single-chip microcontroller, which can deliver an ethanol droplet to the detection chamber through a fine tube to complete the chemical elution process.

### Materials Characterization

The morphologies of materials were characterized by ultrahigh resolution emission scanning electron microscopy (HITACHI SU8010). Information on elemental composition and crystal structure were characterized by infrared absorption spectrometer (Thermo, Nicolet iS50), *in situ* X-ray photoelectron spectrometer (ULVAC-PHI, PHI-5000 Versaprobe III), *in situ* X-ray powder diffractometer (Bruker, D8 Advance). The dye in the hydrogel was observed by a confocal laser scanning microscope (LEICA, TCS SP8MP). All of the in vitro electrochemical characterization was performed using the electrochemical workstation (CH Instruments, CHI660E). The electrical characterization was conducted using the multichannel Data acquisition system (Keithley, model DAQ6510). Photographs were taken by using a high-resolution camera (Sony, α 6400).

### Characterization of the Sensing System

PBS (1×) and ferro/ferricyanide probe solution (5 mM K_3_[Fe­(CN)_6_], 5 mM K_4_[Fe­(CN)_6_] and 0.1 M KCl) were used to characterize the basic kinetic reactions on the electrode surface. EIS measurements were performed in the frequency range 100 KHZ-0.01 Hz in open circuit potential under an AC 10 mV perturbation. Glucose solutions were prepared using PBS (1×) with a range from 0 to 200 μM, while cortisol solutions were prepared using absolute ethanol with a range from 0 to 500 nM. Both sensors were characterized using the chronoamperometric method at a potential of 0 and 0.3 V for glucose and cortisol, respectively. The artificial sweat was prepared according to the European standard EN1811 with 250 mg of NaCl, 50 mg of lactic acid, and 50 mg of urea mixed in 45 mL of deionized water. Iontophoresis operation was performed using a current source (Model 6211, Keithley) at a frequency of 1000 Hz and a duty cycle of 50%. The thermistor was connected in series with a constant resistance of 1000 Ω, and the temperature dynamic was characterized by recording the voltage across the constant resistance. Pulse signals are characterized by recording the voltage across the interdigital electrodes at a sample frequency of 1000 Hz and an applied potential of 5 V.

### Ethical Statement

All human experiments were conducted in accordance with the protocol approved by the Ethics Review Committee of the Harbin Institute of Technology (HIT-2023041). All participants had a detailed understanding of the experimental protocol and signed an informed consent form before the experiment.

### Clinical Experiments

#### Subject Recruitment

All healthy subjects were recruited from the City University of Hong Kong and neighboring communities. The patients with SAD or depression were recruited from the Huazhong University of Science and Technology Union Hospital (HUSTUH) and neighboring communities. The gender ratio was ensured to have no significant difference in each experiment. The subjects of the chronic stress testing included 23 patients with depression (10 males and 13 females, age range from 27 to 71) and 26 healthy subjects (15 males and 11 females, age range from 22 to 67). The diagnosis of SAD met the criteria in the fifth edition of the Diagnostic and Statistical Manual of Mental Disorders (DSM5). The diagnosis of depression was confirmed by a combination of physical characteristics and scales which included SCL-90, SDS, SAS, Hamilton Depression Rating Scale (HAMD) and Kessler Psychological Distress Scale (K10). All subjects need regular dietary and daily routines. Patients with Cushing syndrome, hypertension, diabetes, heart disease, metabolic abnormalities, and other immune diseases and women who are pregnant or during their menstrual period were excluded from the recruitment protocol. People with a heavy coffee diet or exercise habits were also excluded, except in the experiments aimed at investigating the specific influence of lifestyle habits.

#### Lifestyle Habits Influence Testing

All participants need to have stable lifestyle habits (exercise, coffee diet, or inverted daily schedule) in the recent past for a long period (at least 3 months). To investigate the influence of exercise or habitual coffee intake, the subjects were asked to wear FIRES and sit quietly for at least 30 min to calm down, and relevant data was collected at the same time period (around 16:00). To investigate the difference in the biomarker panel between people with regular and inverted daily schedules, subjects were required to have a similar diet. Three FIRES were worn on the arm and wrist of subjects, and the data was monitored by an App in real-time to prevent signal damage or loss. The testing would be restarted if there is a data loss of up to 2 h.

#### Modified TSST

The stress intervention process in the modified TSST included three parts: (1) visual puzzles (10 min). Subjects were asked to identify all the differences between two sets of images. (2) Mental arithmetic exam (10 min). Subjects were asked to quickly mentally calculate the examiner’s arithmetic questions and loudly announce the result. (3) Off-script Speech (10 min). Subjects are asked to give an impromptu speech on a randomly asked topic in front of a group of examiners. At the beginning, the subjects were taken to the waiting room to sit quietly for 10 min, and then, the examiner loudly introduced the experimental content to the subjects. After 10 min, subjects were taken to another testing room to conduct the three parts of stress intervention. Finally, the subjects were taken to the waiting room for rest. The examiner will urge the subjects during the exam to increase the patient’s stress. The entire intervention process was recorded by the camera, and the procedure of video recording observed by the subjects was an important component to intensify stress stimulation. Subjects are required to wear FIRES to record the biomarker data every 10 min from the fifth minute after the beginning.

#### Stressor Investigation

The selected stressors included the competitive video game, action movie, physical examination, psychological examination and speech. In detail, three subjects are asked to wear the FIRES to play a racing game on a computer (20 min), watch an action movie clip (20 min), take a physical examination including routine physical examinations and blood routine test, take a psychological examination including 120 Q&A questions and 100 multiple-choice questions, and complete a speech in front of three judges (10 min). Before each testing, the subjects are informed of the contents of the subsequent action and required to wait for testing to begin. The data were recorded in the waiting period and after the testing (10 min after stressor intervention in the competitive game and action movie set). All the testings were conducted in the same period (around 16:00) on different days. Resting for 10 min served as the control group.

#### CPT

A total of 12 subjects are asked to immerse their hand into a bucket containing a mixture of ice and water (4 °C) for 1 min. The FIRES was worn on the other hand to collect data before and 3 min after the testing along with the STAI-S score. On the day before the test, sweat glucose concentration was collected from the subjects at 8:00, and sweat cortisol concentration was collected at 8:00 and 16:00 a.m. for data baseline fitting. Subjects who failed to persist were excluded from this experiment. Immersing the hand in water at room temperature (25 °C) was conducted as the control testing of CPT. CPT testing was conducted at random times throughout the day, but try to avoid mealtimes as much as possible.

#### SAD Testing

The patient with SAD was required to wear FIRES and wander freely. The data was collected when the patient was sitting alone. Then the patient was asked to walk to a crowded subway station and have a relatively loud telephone conversation with us. The data was recorded and sent to the caregiver’s mobile phone in the distance using a WebService interface. The benchmark values of glucose and cortisol were measured 1 day in advance and input in the developed App.

#### Depression Testing

The glucose and cortisol benchmark values and the conventional demographical information (Age, gender, height, weight, and mean BP) of all the subjects were recorded on the day of clinical diagnosis. The subjects were asked to wear the FIRES and measure their biomarker levels three times at any time on the same or different days. Meal period was avoided as possible. Three patients with mild depression were asked to undergo a 1 week mindfulness meditation treatment, which included a series of actions such as meditation and deep breathing. The process of mindfulness meditation was guided by professional doctors and lasts at least 2 h a day. The biomarker values were recorded before and after the mindfulness meditation intervention.

#### Temporal Data Fitting

Glucose and cortisol data were collected from 11 volunteers to pretrain the numerical model for temporal data fitting. No strict constraints on daily activities are required, but the glycemic index of each meal was monitored to ensure that there were no substantial variations. Seasonal-Trend-Loess (STL) was utilized to derive the representative characteristics of the biomarker dynamics. Different mathematical models were selected, and the curve_fit method from the Scipy library was utilized to find the most suitable model. Bounds were set according to the data ranges, and the maximum number of function evaluations was set as 5000. The leave-one-out cross-validation approach was utilized to evaluate the fitting performance and generalization ability of the models by final average loss.

#### Machine Learning

The data collected from 23 depression patients and 26 healthy controls were labeled manually (each subject was collected 3 times). The data set was divided into the training data set and test data set with a ratio of 117:30 (around 4:1). The models were built using Python v3.8. All of the features were normalized before input into the model. For depression state classification, five ML models including RF, SVM, Bayes, XGBoost, and ANN were selected to train the classifier. RF outperformed other models and was utilized for further classification application. For depression level regression, linear regression was utilized. There is no requirement for the use of an advanced GPU due to the small framework of the model.

#### Fat-Loss Intervention Monitoring

A volunteer who had no previous fitness habits was asked to conduct a two-week intervention regimen. One hour of systematic strength training was performed using a pair of 7 kg dumbbells every day, followed by a 30 min of 30° climbing (aerobic exercise). The diet was controlled to maintain an 800 kcal caloric deficit using a calculation App (Mint Health). Day 10 was set as the break day, during which there are no training arrangements or dietary restrictions. The biomarkers include HR, ST, sweat glucose, and cortisol levels were recorded at 8:00 and 20:00 every day, as well as demographical information including body weight and mean blood pressure. The glucose and cortisol data recorded at 20:00 are converted to predicted values at 8:00 by using the developed temporal process method before being imported into the machine-learning model.

## Supplementary Material









## Data Availability

All data supporting the results of this study are available within the paper and its Supporting Information. Source data are provided with this paper.

## Notes

Supporting Movie 1: Characterization of actuator vibration performance by the reflected light (MP4)
Supporting Movie 2: On body stress monitoring using the FIRES (MP4)
Supporting Movie 3: Development of the ethanol-in-air structure (MP4)
Illustration of the research background and the building of the biomarker panel; strategy for cortisol regeneration; heart rate readout workflow; illustration of the microfluidic optimization; temporal coupling strategy of biomarker results; definition of stress state and indicators; fabrication of the device; characterization of the sensing module, the actuator-based warning module and the circuit control module; and on-body characterization of stress monitoring capability and performance using FIRES (PDF)
