# Supplementary material for: Assessment of Acute Stress and Chronic Stress for Mental Health Management by a Fully Integrated Wearable Biosystem
Source: ACS Nano. 2025 Nov 6;19(45):39192–209. doi: 10.1021/acsnano.5c12395 (PMC12817249; doi:10.1021/acsnano.5c12395)
Supplement: Supplementary file 4 [file nn5c12395_si_004.pdf]

Assessment of acute stress and chronic stress for mental health management by a fully integrated wearable biosystem

Hongwei Chu[1,2†], Wooyoung Park[2,†], Yue Hu[3,†], Zhenhe Huang[4], Rui Shi[2], Jiyu Li[2,5], Zhiyuan Li[2], Mengge Wu[2], Yuyu Gao[2], Guangyao Zhao[2], Xingcan Huang[2], Chun Ki Yiu[2,5], Binbin Zhang[2,5], Dengfeng Li[2], Kuanming Yao[2], Jian Li[2,5], Yuan Guo[2], Ya Huang[2], Qiuqian Ou[1], Guoqiang Xu[2], Pengcheng Wu[2], Jiao Yang[1], Yingchun Li[1]*, Xinge Yu[2,5,6,7]*

[1]Department of Biomedical Engineering, Harbin Institute of Technology (Shenzhen), Shenzhen, 518055, China

[2]Department of Biomedical Engineering, City University of Hong Kong, Hong Kong, China

[3]School of Pharmacy, Shenzhen University Medical School, Shenzhen University, Shenzhen 518060, China

[4]Department of Geriatrics, Xiehe Shenzhen Hospital, Huazhong University of Science and Technology, Shenzhen, 518052, China

[5]Hong Kong Centre for Cerebro-Cardiovascular Health Engineering, Hong Kong Science Park, New Territories, Hong Kong, China

[6]Institute of Digital Medicine, City University of Hong Kong, Hong Kong, China

[7]Hong Kong Institute for Clean Energy (HKICE), City University of Hong Kong, Hong Kong, China

† These authors contributed equally to this work.

*Emails: liyingchun@hit.edu.cn (YL); xingeyu@cityu.edu.hk (XY)

Table of content

Note S1 | Clinical significance of stress monitoring
Note S2 | The physiological responses to stress and associated biomarkers
Note S3 | Methods and equipment for mental stress detection
Note S4 | Illustration of the building of the biomarker panel
Note S5 | The calculation of electrochemical active area
Note S6 | The strategy for the regeneration of the cortisol biosensor
Note S7 | Heart rate readout
Note S8 | Optimization of the microfluidic design
Note S9 | Temporal coupling of biomarker results for minimizing the influence of intra-individual variances
Note S10 | The building of the stress comprehensive indicators
Note S11 | The definition of depression state using scales
Figure S1 | Physiological responses to stress stimulations
Figure S2 | Fabrication process illustration of the integrated sensing system
Figure S3 | Optical images of the FPCB
Figure S4 | Characterization of NiCAT
Figure S5 | Characterization of double-layer capacitance of NiCAT-modified electrodes
Figure S6 | Fabrication of cortisol sensors
Figure S7 | Sensing performance of MIP and NIP-based electrodes
Figure S8 | Selectivity and anti-interference capability characterization of the cortisol
Figure S9 | Reproducibility and stability of the cortisol electrode
Figure S10 | Characterization of the template removal process
Figure S11 | The influences of MIP fabrication on the regeneration effect
Figure S12 | The influence of monomer/template ratio on the regeneration effect
Figure S13 | Characterization of the regeneration performance
Figure S14 | Interface stability of glucose electrodes prepared by different methods
Figure S15 | Methodological characterization of glucose electrodes
Figure S16 | Co-sensing crosstalk characterization of the sweat sensors
Figure S17 | The on-body detection performances of sweat sensor array
Figure S18 | Characterization of the temperature sensor
Figure S19 | Compensation of glucose sensors
Figure S20 | Pressure-sensitive characteristics of the sponge-based sensor
Figure S21 | Characterization of the piezoresistive sensor for HR detection
Figure S22 | Workflow of HR value acquisition
Figure S23 | Sensing performance under repeated usage
Figure S24 | Characterization of drug release using the iontophoresis module
Figure S25 | Skin dynamics during iontophoresis process with different types of applied current
Figure S26 | Characterization of the micro-columns in the inlet
Figure S27 | Optimization of the channel interface
Figure S28 | Long-term interface hydrophily of the microfluidic channel
Figure S29 | Effects of microfluidic inlet numbers on the sweat flow
Figure S30 | Effects of iontophoresis electrode and microfluidic patterns on the sweat flow
Figure S31 | Simulation characterization of sweat outflow at microfluidic outlet
Figure S32 | Characterization of on-body sweat induction process
Figure S33 | Characterization of the electromechanical actuator
Figure S34 | Numerical simulation of the on-body actuating effect
Figure S35 | Influence of vibration frequency on the actuator output
Figure S36 | Block diagram of the FIRES for data collection and transmission

Figure S37 | Detailed circuit schematics of the wearable system
Figure S38 | Biomarker monitoring during different states
Figure S39 | The variances of four biomarkers collected by the other two subjects within one week
Figure S40 | The biomarker levels within people with and without coffee diet
Figure S41 | Chemical elution for cortisol regeneration
Figure S42 | Influence of daily schedule on cortisol levels
Figure S43 | Influence of daily schedule on the levels of ST, HR and sweat glucose
Figure S44 | Dynamics of the biomarker panel during the TSST
Figure S45 | SSCI1 indexes during the TSST
Figure S46 | The influences of different actions on the SSCI1 value
Figure S47 | Function fitting of daytime test data for glucose and cortisol
Figure S48 | Data processing of cortisol and glucose for minimizing the influence of intra-individual variances
Figure S49 | CPT for AS stimulation
Figure S50 | Influences of CPT and control test on the biomarker levels
Figure S51 | The concept of home care of chronic mental health disorders
Figure S52 | PCA results of the collected data
Figure S53 | Performance of models for patient identification
Figure S54 | Continuous SSCI index and classification analysis over the daytime with and without data temporal processing
Figure S55 | Stress level evaluation during the fat-loss intervention phase
Table S1 | Main biomarkers and their response characteristics with stress
Table S2 | Review of portable stress detection device
Table S3 | Spike-and-recovery assays of cortisol and glucose
Table S4 | Selected function for glucose data fitting
Table S5 | Selected function for cortisol data fitting
Table S6 | Comprehensive comparison with related stress management works

Note S1: Clinical significance of stress monitoring

Stress is generally regarded as an adaptative response to external stimulations in physical, psychological or social forms, which was first characterized by Hans Selye in 1936 and has become a pressing global concern now[1]. Several other conceptual models surrounding stress have also been established, such as the Cognitive Appraisal and Coping model[2], Tend-and-befriend model[3] and Conservation of Resources model[4]. However, nearly all conceptual models emphasize that when homeostasis within the organism is threatened, the organism exhibits specific and/or nonspecific responses to the stressor in order to maintain a new state of equilibrium. If the new equilibrium is disrupted, the system will continue evolving until a collapse occurs, prompting the search for equilibrium within other systems. Stress can be classified into acute stress (AS) and chronic stress (CS) based on the nature of the stimuli involved. AS typically features a short duration and high intensity, which can induce different time-domain reactions including acute psychogenic reactions and delayed effects. Comparatively, CS results from cumulative exposures of varying intensities over an extended period. They engage distinct physiological coping mechanisms but also have strong associations and synergistic effects that contribute to the ultimate functional state of the human well-being[5, 6]. Stress affects a diverse range of populations, encompassing students, frontline workers, homemakers, leaders, soldiers, and specialized professionals. Surveys indicate that nearly 74% of individuals experience high stress levels at least one day per week, and four in ten adults worldwide report having encountered significant stress[7]. In the decade preceding the COVID-19 pandemic, issues related to stress had been on the rise, gradually evolving into a new global health crisis.

From the perspective of body perception outcomes, stress can also be categorized into eustress and distress[8]. The former usually refers to moderate stimulation with short duration. It may promote the metabolism of substances in the body and activate the reserve capacity of organs, which supports improved adaptability, defensive mechanisms, and resilience against harm during crises, and also conducive to improving cognitive judgment and ability to respond to various events. Nevertheless, overloading stress can trigger a reversal of beneficial effects——distress or pathological stress, which include some AS and almost CS settings. These usually refer to the stress caused by intense or prolonged stressors that might deplete the body's capacity reserves and defense mechanisms, resulting in metabolic disorders and organ dysfunction. The stressors can be diverse and ubiquitous, such as examinations, interviews, intimidation, bullying, domestic violence, emotional disputes, traffic accidents, illnesses,

natural disasters, and so on. Severe distress can precipitate a series of deterioration reactions for body homeostasis, and ultimately culminating in serious physiological and mental diseases, such as stress ulcer, anorexia, depression, anxiety, autism, sensory dysfunction, and post-traumatic stress disorder. Moreover, it is also associated with an elevated risk of numerous immunological diseases, such as metabolic syndrome, cancer and cardiovascular disease[9, 10].

Stress has a profound impact on human health, and several models have been proposed to elucidate this impact, such as the stress-exposure model[11], stress-generation model[12], and diathesis-stress model[13]. A wealth of evidence has been found to support the link between stress and psychophysiological disorders. For example, the dysfunction of the stress-related axis is highly correlated with the therapeutic effect and recurrence risk of depression[6]. Experimental findings on the stress neuroendocrine metabolism in mice reveal that mutations in receptors of the metabolic axis products can modulate anxiety, aggression, and cognitive abilities[14, 15]. The direct impact of certain drugs on the expression and agonistic/antagonistic effects of these receptors can significantly influence the antidepressant effects[16]. Moreover, clinical diagnostic tests reveal that the cortisol response in healthy individuals from families with a high genetic burden for depression falls between that of acutely depressed patients and normal control groups[17]. This suggests that alterations in the stress pathways and changes in neural excitability may be a hereditary trait, potentially increasing the risk of developing depression or other stress-related disorders in the future. Moreover, the concern surrounding mental stress not only affects individual health and well-being but also imposes considerable strains on societal development. Research indicated that approximately 15% of the loss in life expectancy can be linked to mental health disorders[18]. The number of individuals suffering from mental health disorders exceeded 900 million by 2019, reflecting a 48.1% increase over the previous three decades[19], with numerous determinants of poor mental health outcomes being intensified by pandemics such as COVID-19. Anxiety disorders have emerged the sixth leading cause of disability across both high- and low-income nations with prevalence rates ranging from 0.9-28.3%[20]. The economic impact of mental health issues was approximately $1.6 trillion in 2019, and projections suggest this figure may rise to $2.5 trillion by 2030[18]. The shadow of stress on individual health and society development is becoming increasingly prominent, enhancing comprehensive understanding, perception, and effective control of stress become an urgent imperative for the development of human beings and society.

Note S2: The physiological responses to stress and associated biomarkers

Stress can be regarded as actions of the body restoring homeostasis through multiple coordinated reactions after being exposed to stressors[6], which can lead to complex and significant variations at molecular, cellular, neural and behavioral levels. The responses are predominantly regulated by two physiological pathways: the Hypothalamic-Pituitary-Adrenal cortex (HPA) axis and the Locus coeruleus-Sympathetic-Adrenal-Medulla (LSAM) axis[6, 21] (Figure S1). Specifically, the cerebral cortex receives stimuli from stressors and conveys information to the limbic system via neural impulses. The limbic system serves as the emotional control pivot through several major structures such as hippocampus, amygdala and hypothalamus[22]. After the appraisal of the incoming sensory stimulus, it promotes the synthesis and secretion of arginine vasopressin (AVP) and corticotropin-releasing hormone (CRH) in the paraventricular nucleus of the hypothalamus. Subsequently, these hormones are released into the hypophyseal portal system through the axonal terminals in the median eminence, further transported to the pituitary gland, thereby promoting the release of adrenocorticotropic hormone (ACTH). ACTH enters the blood circulation and reaches the adrenal cortex, thereby stimulating the synthesis of glucocorticoids (cortisol in humans and corticosterone in rodents). Lipid-soluble glucocorticoids enter the cell via passive diffusion and regulate stress responses by binding to two types of hormone receptors (mineralocorticoid receptors, MRs and glucocorticoid receptors, GRs) and initiate transcriptional reprogramming[15]. These two receptors are extensively co-expressed in neurons of the limbic system, although their affinities differ greatly and they have antagonistic effects in stress responses. In the early stages of coping with stress, MRs initiate rapid changes in the relevant neural circuits, facilitating adaptive responses to stress events. When there is an excess secretion of glucocorticoids, the slower response mediated by GRs will subsequently be activated to prevent the initial defense reactions from becoming destructive due to overactivation[14]. Studies have also found that glucocorticoids can interact with the high-affinity membrane coupled receptor 97 (GPR97) on leukocytes to regulate the development and inflammatory responses of immune cells[10]. The LSAM axis (often referred to as the SAM axis) also serves a pivotal role for stress responses[10]. The peripheral receptors in the body transmit stimulus signals from stressors to the locus coeruleus, which is rich in both ascending and descending noradrenergic neurons and acts as the structural foundation for emotional, cognitive, and behavioral changes during stress. Locus coeruleus regulates sympathetic nervous system tension and promotes the release of catecholamines, especially noradrenaline from widely distributed synapses and adrenaline from the adrenal medulla. The overloaded catecholamines can cause further changes in

endocrine response, such as a decrease in insulin levels, an increase in glucagon and growth hormone levels. Besides, the parasympathetic nervous system (PNS) also plays a significant role during stress reactions by promoting the “rest and digest” response and aids in the body’s recovery[23]. There is a strong mutual influence and synergistic interaction between the HPA axis and the LSAM axis to jointly regulate stress responses and activate other neuroendocrine and immune pathways. For example, it has a significant impact on cardiac output capability, which is manifested as increased cardiac contractility, blood flow rate, and heart rate (HR) as well. Excitation of the sympathetic nervous system may also cause constriction of peripheral small blood vessels and increased peripheral resistance, which can potentially further increase the body's blood pressure. When this effect manifests in the stomach, it will significantly reduce the blood perfusion of the mucosa, damage the barrier of gastric mucus, increase the secretion of gastric acid and pepsin, and ultimately cause stress ulcers. Additionally, the perception of stress stimulation and overexpression of related genes and metabolic pathways might also have a significant influence on the utility of the thermoregulation center, which induces some variations in the body temperature during stress process[16].

AS and CS are triggered by stimuli with distinct attributes and elicit varied neuroendocrine and genetic reactions. AS typically initiates swift stress responses marked by pronounced metabolic and neural reactions but generally reverts to baseline levels within minutes to hours. CS develops when the body is exposed to stressors over an extended period, leading to overexpression of metabolic pathways and genes. It typically exists in a state of “allostasis” characterized by limited turnover of endocrine products. The hypothalamic activation on the pituitary shifts from primarily CRH to AVP. The elevated levels of cortisol are, in part, due to reduced cortisol metabolism[6]. However, more clarified mechanisms underlying the body’s genomic behavior responses to AS and CS remain largely unexplored.

Stress activates a series of neuroendocrine responses and induces significant variations in biomarker levels. These biomarkers involve physical, chemical and genetical information and could be valuable indicators of stress level along with behavioral characteristics, and even exhibit more accurate and effective performance in the assessment, discrimination and therapy process. Cortisol, known as “stress hormone”, is the end product of the HPA axis and can be considered the most representative biomarker reflecting the state of human stress response. Cortisol circulates throughout the body via the bloodstream, regulating the initiation, recovery, and cessation of stress responses by binding to MRs and GRs. Simultaneously, it plays a crucial

regulatory role in energy modulation, immune response, and inflammatory reactions during stress reactions. Significantly, cortisol can diffuse into various biological fluids, such as blood, saliva, sweat, urine, hair and interstitial fluid, which makes it more accessible and become the focus of numerous studies. The upstream products of the HPA axis, such as CRH and ACTH, can also effectively indicate the stress state, although they may not be as prominent in terms of sensitivity in stress response. Studies have found that ACTH will return to near basal levels while cortisol remains increased level during the long-term activation process[24]. Changes in blood glucose levels also serve as a significant event in the stress response. The specific neural circuit mechanisms are sophisticated and have not been fully elucidated, but research has revealed that excessive secretion of cortisol during stress can promote gluconeogenesis[25]. Additionally, the release of catecholamines, products of the LSAM axis, can inhibit insulin synthesis and promote glucagon, thereby reducing glucose utilization and accelerating glycogen breakdown. The catecholamines like adrenaline and noradrenaline, served as end-products of the LSAM axis, are also crucial components of the stress response. Recently, it has been discovered that alpha-amylase can also react to stress through the LSAM axis and has the potential to amplify the release of low concentrations of noradrenaline[26]. Therefore, it holds promise as a potential substitute for noradrenaline as a stress marker. Secretory Immunoglobulin A (S-IgA) is a primary immunoglobulin in the human body that can also be activated by the effects of stress[27]. S-IgA can be locally released under the control of the sympathetic nervous system during AS. However, in CS, sustained glucocorticoid exposure may potentially inhibit the synthesis of S-IgA[27]. Therefore, S-IgA provides varied responses in different stress scenarios. Additionally, many other chemical biomarkers have been found to react under various stress scenarios, such as dopamine, ketones, magnesium ions, interleukin-6 and so on[28-31].

Stress responses can also induce changes in various physical indicators in the human body. Vasoconstriction and increased cardiac output are typical characteristics, leading to elevated blood pressure and a faster HR. Additionally, HR variability (HRV) is considered an effective marker of stress response. Studies suggest that HRV decreases under stress, however, some others reached the opposite conclusion[32]. Stress can also induce changes in body temperature by enhancing the vibration of skeletal muscles. Recent investigations propose that the activation of a major psychosocial circuit from the dorsal peduncular cortex and dorsal tenia tecta to the dorsomedial hypothalamus (DP/DTT→DMH) may be a key factor in the elevation

of body temperature under stress conditions[16]. Table S1 summarizes the main biomarkers and their associations with stress activation.

Note S3: Methods and equipment for mental stress detection

Stress monitoring and determination are of great significance for health care and individual development. However, a clinically validated and precise methodology for assessing human stress levels is lacking. Patients with abnormal stress states are usually diagnosed in the hospital based on their behavioral manifestations (Appetite, sleep status, body, reaction ability, etc.) and self-reported psychometric tools, such as Symptom Checklist 90 (SCL-90), Self-Rating Anxiety Scale (SAS), Self-rating Depression Scale (SDS), Psychological Stress Scale (PSTR). These scales usually include several queries that aim to quantify the degree to which one feels "uncontrollable," "overloaded," "unbearable," etc. about oneself or one's circumstances. While these tools have proven effective in particular clinical evaluations, their inherently subjective nature poses significant challenges in achieving consistent and accurate assessments across diverse populations. Assessing changes in physiological indicators related to stress appears to be attractive for evaluating stress status. Focusing on genomics to detect intra-cellular gene expression levels and molecular epigenetic modifications might be a reliable method for assessing stress levels. However, this approach is constrained by technological capabilities and practical applicability, making it challenging to achieve widespread implementation in real-world settings outside the laboratory. Using some point-of-care devices or wearable equipment to monitor the indicators of human body could be a more affordable and effective method for stress detection. Table S2 outlines various prevalent commercial devices and developed portable biosensors for stress detection. Commercial devices usually focus on some physical vital signs like HR, HRV and electrodermal activity (EDA). These devices can play a certain assistance role in daily health monitoring, but fall far short of achieving precise management of stress levels. Numerous similar studies indicate that these devices perform inadequately under conditions of stress induced in real-world scenarios, and consistently necessitate external supplemental measures to accurately diagnose stress-related responses[33]. With the aid of development of material science and flexible electronics, some researchers are dedicated to developing portable sensors for ex situ or in situ monitoring stress-related biomarkers. Cortisol is the predominant target and always be detected by electrochemical sensing methods based on specific recognition media like antibody[34, 35] and aptamer[36]. These portable sensors can achieve very low detection limits and contribute valuable insights into stress assessment. However, they still cannot discern and analyze complex stress scenarios. Attaining this objective necessitates meticulous consideration across several levels, including panel identification, detection methodologies, device architecture, and analytical algorithms.

Note S4: Illustration of the building of the biomarker panel

Wearable bioelectronics offers a promising approach for evaluating the state of mental stress in an in situ and real-time manner. A critically important prerequisite is to establish a sufficiently precise and effective biomarker panel. As illustrated in Note S2 and Table S1, plenty of biomarkers, including gene fragments, hormones, proteins, cytokines, ions, as well as certain physical indicators and behavioral signs, are closely associated with stress response. However, some of them are extremely limited for incorporation into the monitoring panel due to special requirements of stress assessment in clinical practices. The biomarker will not be considered until it meets the following criteria: 1) it has a definite and intensive correlation with AS or CS; 2) it is readily accessible and can be determined through a relatively portable way; 3) it possesses a high specificity towards stress response and is not easily disrupted by other physiological events.

For instance, some genomic and cellular features like the leukocyte adhesiveness ratio can provide the most direct and compelling clues for analyzing stress states[37]. However, they generally highly relied on advanced equipment and elaborate experiment conditions. Similarly, some hormones like CRH or ACTH act as the upstream product of the stress axis and regulate the secretion of the corticosteroids. It is extremely challenging to determine them in a point-of-care setting. Moreover, they are usually secreted and exist in the bloodstream, which is inaccessible in a non-invasive manner. The catecholamine products of the LSAM axis, such as epinephrine and norepinephrine, are typically secreted within an extremely brief timeframe following stress stimuli, followed by rigorous regulation through synaptic reuptake and plasma clearance. Therefore, they are unsuitable as direct biomarkers of the stress response; instead, often indirectly characterized by the downstream parameters such as cardiac output capacity and cardiovascular dynamics. Some ions like K^+, Ca^+ have been used in some work to profile the stress state. However, the association between these ions and stress responses is still not particularly clear, necessitating further investigation into their roles within stress metabolism pathways. Some commercial devices incorporate physical biomarkers such as EDA, HRV and its frequency-domain indices into valuable collections. However, these devices can only be used for superficial qualitative assessments. These metrics lack specificity for stress responses, and HRV exhibits inconsistencies and occasionally contradictory trends regarding stress responses across different studies[32, 38, 39]. Additionally, substantial intrinsic fluctuations in HRV measurements were observed in our preliminary experiments, as well as poor reproducibility across repeated trails for identical stress stimuli. Moreover, EDA is

significantly influenced by sweat secretion, which poses a considerable interference in achieving accurate stress assessment. Therefore, these parameters are discreetly excluded from the monitoring panel.

In view of this, we build a biomarker panel based on cortisol, glucose, skin temperature (ST) and HR for stress monitoring. These biomarkers play a paramount role in main regulatory pathways of stress stimulation. Cortisol is the most predominant hormone which runs through the entire process of response, regulation, and recovery to stress stimuli. A series of changes can be triggered by stress to regulate glucose metabolism behavior, which can be directly reflected by the glucose concentration. Moreover, glucose is concurrently regulated by cortisol; therefore, the cascading effects of both can assist in the time-sequential biometric signature profiling of stress response. For realizing real time and in situ stress monitoring, sweat analysis is employed due to its non-invasive nature and demonstrated correlation with blood determination. It should be noted that there are time lags between the responses in sweat and blood during stress exposure. However, for analytes such as cortisol or glucose, this lag typically ranges from several minutes to over ten minutes[40-42], primarily due to the dense vasculature surrounding sweat gland secretory coils which substantially shortens the diffusion delay[43, 44]. Additionally, accelerated sweating rates induced by stress may further diminish this lag to a certain degree[42, 45]. ST and HR are the most direct indicators of muscular activity and cardiac output capabilities, playing a crucial regulatory role in stress responses. It should be noted that determining the temperature in rectal or tympanic membrane is the most suitable approach for accurate assessment of the core body temperature variance, with alternative determinations possible at sites such as the oral cavity or axilla[46, 47]. But considering practical operability, we use skin temperature as the analysis parameter for stress effects, which has been proven to hold the capability to reflect the degree of stress to a certain extent[48, 49], and its environmental impact can be reduced due to our encapsulation effort on the device. All of these four biomarkers have been confirmed to have a clear and specific correlation to the stress response, and they are readily available and integrated within the overall analytical framework. Moreover, there should be a criterion 4) that mandates that biomarkers fulfill the requirements for continuous monitoring. It is challenging for cortisol to meet this criterion because it is generally measured through affinity-based approaches. In this regard, we developed a simple two-step strategy for the regeneration of cortisol electrodes (Note S6), which enables continuous detection of cortisol levels and renders the entire monitoring system operates in a

sustainable manner. Collectively, these four biomarkers were selected to construct the biomarker panel for effective analysis of AS and CS.

Note S5: The calculation of electrochemical active area

The electrode surface area was first determined based on the Randles-Sevcik equation (Eq 1), which is a mathematical model of current reaction in electrochemical systems.

$$I_p = 0.4463nFAC\sqrt{\frac{nFvD}{RT}}(1)$$

Where I_p is the peak current in the redox reaction in the CV scanning (in A), n is the number of electrons transferred in the redox event, F is the Faraday's constant (96485 C mol^{-1}), A is the active surface area (in cm^2), C is the concentration of the redox species, v is the scan rate (in V s^{-1}), D is the diffusion constant (in cm^2 s^{-1}), R is the Gas constant (8.3144 J mol^{-1} k^{-1})and T is the temperature (in K). We use the ferrocene/ferrocenium couple with a diffusion constant of 6.7 $\times 10^{-6}$ cm^2 s^{-1} to serve as the redox species, and when the reaction is carried out at room temperature (25 °C), the Eq1 can be simplified as:

$$I_p = 268600n^{\frac{3}{2}}AD^{\frac{1}{2}}Cv^{\frac{1}{2}}(2)$$

The concentration of redox species is 5 mM and the scan rate is 0.05 V s^{-1}. We determined the CV plots of the bare Cr/Au electrode and the electrode modified with NiCAT (Cr/Au/NiCAT). It was found that the peak current of the Cr/Au electrode is around 188.27 µA, while the peak count current of the Cr/Au@NiCAT electrode exhibits a significant enhancement (around 385.25 µA). Therefore, the electrochemical surface areas are 0.242 and 0.496 for the Cr/Au electrode and Cr/Au@NiCAT electrode, respectively.

Note S6: The strategy for the regeneration of the cortisol biosensor

The regeneration of biosensors holds a significant value in facilitating cost-effectiveness and sustainability, and more importantly, it is an essential prerequisite for ensuring real-time and long-term operation of wearable systems. The detection effect of sensors for analytes, especially macromolecular substances, is based on the specific recognition of analytes by functional receptors (typically, enzyme, aptamer, nucleic acid sequence, antibody, MIP, etc). The irreversible binding between receptors and analytes means that sensors can only respond to increasing concentrations of analytes. Numerous studies have dedicated to renewing the biosensor by detaching the analytes from the receptors using diverse approaches like heat, light, chemical, electric field or magnetic field[50]. Overcoming the binding energy between receptors and analytes is the core idea for achieving regeneration of the electrochemical affinity-based biosensors. This typically can be reached by electrode engineering or external intervention. Light or heat based methods are widely used in the modulation of the binding effect between aptamers and target molecules[51]. This approach typically induces conformational changes in the aptamer, facilitating the release of bound molecules and restoring recognition of the target molecules. In a similar vein, some workers utilized the shift of pH to reach comparative purposes in certain aptamer-based biosystems[52]. However, these approaches are extremely limited and possess high demand in the aptamer design. Electrical based methods are typically suitable for repeated detection of electroactive or charged molecules. For instance, Gao's group reported the successful regeneration of MIP-based sensors in two conditions through the intervention of external currents[53]. In the direct detection approach, the electroactive amino acids can be oxidized and removed by a high-voltage amperometry current-time (I-T) operation. In the indirect detection approach, a constant voltage is used to repel the charged amino acids from the MIP cavities, leading to a renewal of the biosensor. Similarly, the magnetic field based methods lie in similar principles for sensor regeneration, which weaken the binding effect of target molecules by regulating magnetic media such as magnetic beads[54]. However, these methods are only effective in detecting molecules with special characteristics. Besides, chemical elution based methods are widely used for sensor regeneration and possess higher generalization ability[55, 56]. But most of these methods involve the utilization of harsh solutions (ultra low- or high-pH solutions), which may corrupt the sensing system and affect the function of receptors. Thus these methods typically demonstrate an elevated regeneration ratio, yet they fall short in terms of effective cycling. Collectively, current research offers a myriad of concepts for sensor regeneration; however, each presents significant constraints. To meet

specific sensing demands, tailored scheme design is essential for achieving optimal system regeneration.

In regards to our built biomarker panel, the only biomarker that hinders the sensing system from achieving repeated continuous detection is cortisol, as it relies on specific bioreceptors for determination. In our work, we utilize the MIP to recognize cortisol molecules. The preparation of MIPs based on an electrochemical deposition approach which involves two steps. In the first electropolymerization step, pyrrole is utilized as the functional monomer and cortisol serves as the template. cortisol becomes incorporated within the polypyrrole matrix after the CV polymerization processing. In the second elution step, repeated CV scanning in PBS is applied to elute the template cortisol molecules. This repeated electric scanning process changes the lattice structure of polypyrrole, thereby diminishing its binding affinity for cortisol. As a result, cortisol molecules are removed from the polymer, leaving cavities that can specifically recognize cortisol molecules. This elution operation provides us with inspiration for developing the cortisol regeneration scheme. Moreover, cortisol possesses a significant difference in solubility compared to other sweat biomarkers. It is less water-soluble with a standard water solubility of 0.32 mg mL^{-1}, while it has a higher solubility in most organic solvents (dimethyl sulfoxide (DMSO) solubility: 36.25 mg mL^{-1}; ethanol solubility: 9.06 mg mL^{-1})[57]. In view of these, we proposed a two-step scheme for the regeneration of MIP-based cortisol sensors: A two-cycle CV scanning operation from -0.2 to 0.8 V was applied and followed with chemical elution with ethanol. The electric scanning operation aims to weaken the binding interaction between the cortisol molecules and the polypyrrole matrix. Then ethanol can further extract the cortisol molecule and remain the specific MIP cavities. It was found this two-step scheme can achieve a nearly complete signal recovery and the electrode can attain a relatively consistent signal when re-recognizing the cortisol (Figure 2g), demonstrating the successful regeneration of the cortisol sensor.

It should be noted that the elution operation of the MIP preparation process will simultaneously cause a reduction in signal intensity due to the diminished conductivity of polypyrrole, severely affecting the sensitivity of the sensor. It was found that the output signal of the electrode continuously decreases as the number of scan circles increases, stabilizing around 30 cycles where the signal exhibited minimal significant fluctuations (Figure S10). Therefore, we set the number of CV scanning cycles to 30 during each electroelution process, which allows the subsequent electric scanning action for regeneration to still weaken the binding effect of

cortisol molecules without interfering with the signal intensity. It was found that the combination of electric scanning and chemical elution results in superior signal recovery efficacy compared to approaches utilizing only a singular operation (Figure S13a).

Moreover, the varying polymerization effects during the preparation of the MIPs can significantly influence the extraction of cortisol. We found that as the scanning rate decreases, the polymer layer on the electrode surface becomes denser, which impacts both the detection performance and regeneration efficiency of cortisol to varying degrees. It was found that the signal recovery ratio reaches the highest when the scan rate is set as 70 mV s^{-1}, but this is accompanied by a reduction in sensitivity attributed to the excessively dense polymer layer impeding the transfer of internal charge probes. After weighing the sensitivity of the sensors against the regeneration effect, we chose a scanning rate of 50 mV s^{-1}, as it offers the highest sensitivity and a considerable signal recovery rate (Figure S11). Similarly, the ratio of monomer and template has a relatively smaller influence on the signal recovery ratio compared to the scan rate (Figure S12). A 5:1 ratio of pyrrole and cortisol was selected based on the performance on sensitivity and regeneration. Different concentration of ethanol was considered in the chemical elution process. It was found that the signal recovery and recovery time are almost consistent when the concentration of used ethanol is over 50%, while outperformed the condition where 25% ethanol was employed (Figure S13b).

Compared to harsh solutions like strong acid or alkali solutions, ethanol is relatively mild and does not significantly damage the electrode surface. However, the continuous elution processes still proposed a demand of a robust electrode interface. We found that the signal recovery rate of the electrode is affected during the continuous regeneration process, typically declining before the fifth cycle of regeneration. However, when the NiCAT layer is assembled on the electrode, the signal maintains stability throughout multiple regeneration operations (Figure S13c). This stability is credited to the compact three-dimensional architecture of the NiCAT layer, which facilitates a more stable interfacial bond with the upper polymer layer. It was found this scheme can achieve an effective regeneration performance over 50 cycles (Figure S13d), ensuring the repeatable detection of cortisol and real-time monitoring of the biomarker panel for stress profiling.

Note S7: Heart rate readout

The detection signal is first processed by a band-pass filter to reduce the background noise and baseline drift. The signal is down-sampled to 1000 points per second. We define a temporary data box to receive the latest test value and traverse the entire signal curve. The maximum value (S_{max}) and minimal value (S_{min}) are recorded and updated during the traverse process. The difference between the S_{max} and S_{min} is calculated and compared to the threshold value, which is determined empirically based on prior pilot testing. Should the computed difference exceed the threshold, the count of peaks is incremented by 0.5. As the temporary data box continues to traverse, the number of detected peaks is constantly updated. The peak interval value (S_{PI}) was simultaneously determined. The prerequisite for effectively identifying peaks the relative difference between S_{PI} and empirical mean peak interval is less than 25%. and the peak is considered and outputted. The latest data point will be discarded and the next traversal process will proceed if this prerequisite is not met. The final peak numbers are recorded and output with minimal delay.

Note S8: Optimization of the microfluidic design

The microfluidic module is designed specifically for precisely and efficiently manipulating secreted sweat. First, three micro-columns (diameter: 182.5 μm) were integrated near the sweat channel in each inlet. The trajectory of secreted sweat can be adjusted to be more inclined towards flowing into the channel due to the hydrodynamic effects[58, 59], leading to an enhanced sweat collection efficiency (Figure S26). A single micro-column is also mounted on the center of the reservoir, which can provide effective support for the upper layer of the microfluidic to prevent the collapse of the reservoir room. The connection and channel shape between the reservoir and the outlet are set as smooth curves as possible instead of straight lines. This design can ensure less obstruction and sweat residue when sweat flows out of the reservoir.

To allow sweat to flow into the microfluidic channel as expected, it is generally necessary to perform hydrophilic treatment on the inlet of microchannels. Plasma treatment is widely used to hydrophilize microfluidic chips. However, hydrophilic microchannels can lead to the residual of sweat on the walls, which significantly impacts the detection of newly secreted sweat. To address this issue, we perform region-specific hydrophilic and hydrophobic modifications within each sweat flow channel (Figure S27). A detachable PDMS layer was utilized to cover the bottom microfluidic layer and the micro-columns in the detachable PDMS divide each microfluidic channel into two sections. After treating the entire channel with plasma, the channels near the inlet were further processed with sodium dodecyl sulfate (SDS) for hydrophilicity and the channels near the reservoir were treated with hydrophobic agents for hydrophobic modification. In this way, each microfluidic channel contains two distinct interfaces, with a small buffer zone of approximately 200 μm between the two interfaces. It was found that the contact angle of the channel decreased from 106° to 58° after plasma treatment, and further modified SDS reduced it to around 15°. The contact angle of the channel was restored to 128° after treatment with hydrophobic agents (Figure 2m). This design allows secreted sweat to enter microfluidic channels while reducing residue on the channel walls. Next, we used blue inks to fill the channel and refreshed the channel with deionized (DI) water. It was found that the channel solely treated by plasma remained distinct blue ink trace, while the channel with the region-specific modification had a significantly reduced ink residue and was restored to the state before filling after two cycles of DI water refreshments (Figure S27b). It should be noted that the hydrophilicity induced by plasma treatment is challenging to sustain over extended periods and usually significantly decreases within a few hours, which is not conducive to the long-term use of the microfluidic module. Besides, the effect of enhancing

hydrophilicity rises with the increase in the duration of plasma treatment, but prolonged treatment may lead to cracks in the microfluidic substrate like PDMS, thereby affecting the transport of sweat in microfluidic channels. Therefore, we combined plasma for a short time (30 s) and SDS treatment (90 ℃, 3 min) for hydrophilic modification. It was found that the contact angle of PDMS after plasma treatment increased continuously over time and reached 100° after 6 hours of treatment. The PDMS treated with plasma and SDS showed a high decrease in contact angle and the hydrophilicity showed no significant change after 6 hours (Figure S28), which is beneficial for the long-term application of the microfluidic module. Based on the optimization by the region-specific modification, we proceeded to detect glucose at various concentrations in a continuous manner. Measurement directly in the standard solution without the microfluidic module served as the control. It was found that the signal was smaller than that detected in the control test when switching from a low concentration test solution (50 μM) to a high concentration one (200 μM). Conversely, in the reverse scenario, the signal was stronger than what was detected in the control test. Comparably, the signals detected using the optimized channel were almost consistent with those detected in the control test, demonstrating an effective optimization in sweat residue (Figure 2n).

The overall structure of the microfluidic module has also been investigated and optimized by numerical simulations. The sweat manipulation process in the microfluidic module with different numbers of inlets was simulated. Glucose with a concentration of 200 μM and flow rate of 1.5 μL min^{-1} flows into the reservoir from the inlet and is discharged from the outlet. It is found that increasing the number of inlets will accelerate the rate of sweat circulation and produce a more uniform distribution of sweat in the reservoir for detection (Figure S29). Therefore, we choose the microfluidic module with 9 inlets for further sweat detection. Moreover, the inlet distribution of the microfluidic module should be designed with reference to the shape of iontophoresis electrodes, as sweat is predominantly stimulated in proximity to the anode electrode, where the carbachol-loaded hydrogel is positioned beneath it. In our design, the iontophoresis electrodes are fabricated into two pieces with a crescent-like shape, and the anode electrode wraps around the entire sweat detection area. We compared our design with common designs in other microfluidic-assisted works by numerical simulations. It found that our design allows sweat to almost fill every part of the detection area after around 3 minutes with a flow rate of 1.5 μL min^{-1}, while other designs may result in inconsistencies in analyte concentrations within the detection chamber (Figure S30). This underscores the reasonability of our design for precise and efficient sweat manipulation and determination.

Note S9: Temporal coupling of biomarker results for minimizing the influence of intra-individual variances

The occurrence and deterioration of mental stress state usually occur in unpredictable manners, so the critical need for real-time monitoring is highly valued. However, most biomarkers, especially chemical substances, are time-dependent in their normative metabolic levels in the human body. For example, cortisol demonstrates a distinct circadian rhythm pattern, while glucose levels are severely affected by daily diet. These intra-individual variances in biomarker levels bring great interference and limitations to the accurate and efficient assessment of health states, so even in clinical in-vitro testing in hospitals, stringent limitations regarding sample collection are often imposed, such as the requirement for fasting in the morning.

For our built biomarker panel, previous experiments have demonstrated that cortisol and glucose have a distinct variance throughout the daytime. ST and HR are relatively stable as long as there is no special event intervention (Figures 3e, f and Figure S43). In this regard, we aim to investigate the specific dynamic pattern of cortisol and glucose level throughout the daytime and try to minimize the influence due to intra-individual variances. A total of 11 subjects were recruited to track their sweat cortisol and glucose levels from 8:00 to 22:00. It is important to acknowledge that the period from 22:00 to 08:00 (next day) typically constitutes a nocturnal rest phase, during which the levels of biomarkers and mental stress state in the body are generally maintained at a stable degree. Monitoring stress during this time period is not of great significance, therefore it is excluded from our experiment. All the subjects were asked to maintain a normal and steady lifestyle, striving to minimize engagements with potential stressors. The cortisol and glucose data are collected per 30 minutes during the test period. The results showed that the cortisol level is at its highest in the morning (usually 8:00 or 8:30) and then decreases with time (Figure S48a). All subjects exhibited similar trends but the amplitude of cortisol fluctuations varied greatly among different subjects during the testing period. The maximum difference in cortisol levels among subjects during the testing period can reach 130 nM, while the minimum is only 34 nM. The glucose levels fluctuated significantly, which was primarily due to the effects of dietary interventions on glycometabolism. The specific patterns of glucose levels have distinct differences among the 11 subjects.

Through observation of the monitoring data from 11 subjects and understanding of biomarker metabolism mechanisms, we employed the Seasonal-Trend-Loess (STL) approach to model the dynamic curves of biomarkers:

$$D = D_T + D_S + D_R (3)$$

where D_T and D_S represent trend and seasonal components respectively, with D_R as the residual term. For glucose dynamics, significant inter-individual variability was observed among volunteers, primarily manifested in baseline levels and amplitude variations of glycemic responses caused by insulin resistance (Figure S48d). In this regard, we standardized the fasting measurement at 8:00 AM as the benchmark, converting test results into relative changes from this baseline:

$$\Delta Glu_t = \frac{Glu_t - Glu_{benchmark}}{Glu_{benchmark}} (4)$$

Post-normalization, it was found that all volunteers exhibited similar dynamic response patterns (Figure S48b). The dynamic curves displayed three distinct peak-shaped segments corresponding to daily dietary influences, which constitute seasonal components D_S. After removing these seasonal elements, the remaining data showed no significant fluctuations, indicating that the core modeling challenge lies in characterizing the three meal-induced peak signals. To achieve this, two modeling strategies were implemented: the first involved holistic fitting of all three peaks using one mathematical model. Sine, Pseudo-Voigt (PsdVoigt), polynomial and inverse polynomial (InvsPoly) functions were selected based on their conceptual relevance and waveform characteristics; the second strategies employed separate function models for each peak while Gaussian and double-exponential peak (DEP) functions were selected. Leave-one-out cross-validation (LOOCV) was utilized to evaluate the fitting merit of these functions: ten sets of data (training datasets) were used for fitting, and the remaining one set of data (validation dataset) was used to calculate the loss from the fitted curve. Each dataset will be selected once as the validation dataset, so there will be a total of 11 configurations for data allocation. The aggregate loss across all configurations was computed to derive a final loss metric reflecting the fitting performance of each function.

Results demonstrated superior fitting performance with the second strategy, where the DEP model outperformed the Gaussian model through lower loss values (Table S4). This superiority stems from the asymmetric nature of DEP function better capturing physiological reality - rapid postprandial glucose elevation followed by gradual decline during metabolism, contrasting with the symmetric Gaussian profile. Therefore, the DEP function was ultimately selected as the fitting model for glucose dynamic data:

$$f(t) = \sum_{i=1}^{3} A_i (e^{k_{1i}(t-t_i)} - e^{k_{2i}(t-t_i)}) H(t - t_i) (5)$$

Where A represents the signal peak amplitude, k_1 and k_2 represent the rising and falling rates of the peak, respectively. $t_{1\text{-}3}$ means the initial peak timepoint, which were taken as 8:00, 12:00, 18:00 in the model. $H(t\text{-}t_i)$ means the Heaviside function, which limits the scope of each peak function. It should be noted that each peak possesses specific parameters A_i, K_{1i}, and K_{2i}, theoretically requiring 9 total parameters. To mitigate overfitting risks in limited datasets, we implemented parameter sharing for K_1 and K_2 across all three peaks based on observed kinetic similarity in glucose rise/fall patterns between individuals and temporal phases. This reduced the final model to 5 parameters (A_1, A_2, A_3, K_1, K_2), effectively balancing model complexity and generalizability.

For cortisol dynamics, all the subjects exhibited a distinct circadian rhythm pattern where the highest cortisol level appeared in the morning and a monotonic decreasing trend existed during the daytime. We first processed the cortisol data like glucose to minimize the data variance:

$$\Delta Cor_t = \frac{Cor_t - Cor_{benchmark}}{Cor_{benchmark}} \quad (6)$$

8:00 a.m. was selected as the benchmark timepoint, when the measurement values are relatively stable in a day in our protocols due to minimal external activity interferences. However, the cortisol data remain quite varied among the subjects, because the circadian rhythm poses different influences on cortisol secretion levels across individuals (Figure S48d). To this end, we introduced the second benchmark point (16:00) for cortisol data processing. The cortisol variance between the two benchmark points is defined as characteristic gap (CG) which reflects the influence level of circadian rhythm on the individual.

$$CG = Cor_{benchmark1} - Cor_{benchmark2} \quad (7)$$

Benchmark time 1 and 2 are selected as 8:00 and 16:00 considering the relatively stable psychological dynamics at these time periods. Then we convert the y-axis to the ratio of the difference between cortisol concentration at that time point and the first benchmark point relative to CG (R_Cor):

$$R_{Cort} = \frac{Cor_{benchmark1} - Cor_t}{CG} \quad (8)$$

It can be found that the cortisol data showed an enhanced consistency after processing (Figure S48e), which means a comparative reduction in the inter-individual variance.

Based on these processed data, we attempt to establish a mathematical model that accurately captures their behavior. It can be found that the cortisol dynamic is predominately influenced

by the D_T components while the D_S can be regarded as zero. In this regard, we selected six numerical functions that have similar signal characteristics to fit the cortisol data: Power, logarithmic, asymptotic regression, rational, exponential associate (ExpAssoc), and exponential decay (ExpDecay) functions. LOOCV was utilized to evaluate the function model performances. It was found that asymptotic regression function possesses minimal final loss, which represented the best fitting merit (Table S5). Therefore, the asymptotic regression model was used for cortisol data fitting:

$$f(t) = A(1 - e^{-B(t-8)}) + C(t-8) \quad (9)$$

Where A refers to the asymptotic value of the exponential part, B refers to the growth rate of the exponential part, and C refers to the growth rate of the linear part.

It can be found that the two mathematical models exhibited considerable fitting performance for the glucose and cortisol monitoring data (Figure S47). We further compared the test data of 11 subjects with the fitting model and calculated the average relative fitting error (ARFE):

$$ARFE = \frac{\sum_i \frac{Data_{measure} - Data_{fitting}}{Data_{fitting}}}{29} \quad (10)$$

It was found that the ARFE of each subject is less than 6%, demonstrating an excellent fitting performance (Figure S48f). The fitting function curves will be used to serve as the dynamic baseline to evaluate the biomarker level instead of a constant baseline, which can minimize the interference of intra-individual variances.

Note S10: The building of the stress comprehensive indicators

We have developed the integrated wearable biosystem based on the built biomarker panel for mental stress determination. Nevertheless, it is needed to generate a numerical comprehensive indicator to map the stress level. Importantly, due to the significant differences in physiological responses between AS and CS, targeted analysis is usually necessary for precisely profiling of the stress state. Herein, we developed three kinds of stress comprehensive indicators (SSCIs) through different algorithmic logics to serve stress assessment in different settings.

First, for a simple and rapid analysis of ordinary stress stimuli, we directly perform an unweighted overlap of all biomarkers and generate the semi-quantitative evaluation indicator. Considering the significant differences in baseline levels of biomarkers among individuals, the relative changes in biomarker concentrations were selected for algebraic calculations to generate a comprehensive indicator (SSCI1):

$$SSCI1 = \sum_{i} \frac{Biomarker_{i_{measure}} - Biomarker_{i_{control}}}{Biomarker_{i_{control}}} \quad (11)$$

Biomarker$_i$ represents cortisol, glucose, ST or HR. The control value is indicative of the biomarker level in the absence of any influencing stressors, typically denoting the measurement taken prior to the exposure to the stress stimulus. Alternatively, when we simply evaluate the level of stress in a certain state rather than assessing the level of stimulation from a particular stressor, the measurement value of the psychological state at a relatively stable moment can be used as the control value for SSCI1 calculation. The SSCI1 is suitable for quick and semi-quantitative evaluation of stress state without the building of specifically trained models in some generalized scenarios, such as comparing the intensity of individual responses to different stress stimuli.

Furthermore, to achieve an accurate quantitative analysis of a specific acute stress stimulus, we made two improvements based on SSCI1 to develop a specific evaluation indicator (SSCI2): First, the significant intra-variances in biomarkers (cortisol and glucose) have a nonnegligible impact on the evaluation of real-time detection results. We have established mathematical models of the dynamic changes of biomarkers through pre-experiments (Note S9), so we can obtain the theoretical baseline level of biomarkers (cortisol and glucose) at each time point based on the numerical functions and pre-detected benchmark values. We will replace the unchanged baseline level with this dynamic baseline level ($Cor_{Baseline\&t}$, $Glu_{Baseline\&t}$) which is time-dependent. There was no significant difference between ST and HR within one day, so

their baseline level can be taken as the test value at any time point without stress interference (measured at 8:00 in this experiment). Second, the degree of impact of an AS stimulus on different biomarkers varies, so we calculated the response weights of different biomarkers to the AS stimuli through pre-experiments. Finally, the SSCI2 can be expressed as:

$$SSCI2 = \sum_i \omega_i \frac{Biomarker_{i_{measure}} - Biomarker_{i_{baseline}}}{Biomarker_{i_{baseline}}}$$

$$= \omega_{Cor} \frac{Cor_{measure\&t} - Cor_{baseline\&t}}{Cor_{baseline\&t}} + \omega_{Glu} \frac{Glu_{measure\&t} - Glu_{baseline\&t}}{Glu_{baseline\&t}} + \omega_{BT} \frac{BT_{measure\&t} - BT_{baseline}}{BT_{baseline}} + \omega_{HR} \frac{HR_{measure\&t} - HR_{baseline}}{HR_{baseline}} \quad (12)$$

The $Cor_{Baseline\&t}$ and $Glu_{Baseline\&t}$ are obtained by the Eq. (8) and Eq. (4). The benchmark values of cortisol are detected at 8:00 and 16:00 and the benchmark value of glucose is detected at 8:00 in advance. For ST and HR, their baseline values are set as the pre-detected value at 8:00. The response weights (ω_i) are obtained by the multinomial logistic regression model based on a specific AS dataset. For proof-of-concept, the cold-pressor test (CPT) was utilized as an instance of AS. Twelve subjects were recruited and stimulated by CPT for the acquisition of the response weights. The levels of biomarkers before and after CPT were collected, and the State-Trait Anxiety Index (STAI)-S results of the subjects were recorded before and after the test as ground truth labels of their psychological states. The relative changes of all biomarkers from baseline are considered as independent variables, and the ground truth labels are considered as dependent variables. The corresponding weight indices are obtained by multiple logistic regression models: ω_{Cor}=14.788, ω_{Glu}=40.834, ω_{HR}=48.219. ST results showed no significant correlation with the psychological ground truth labels in this test (Figure S50), so ST was excluded from generating the SSCI2 for the investigation of CPT stimuli. We classified stress states into three categories based on STAI-S results with 34 and 44 as the cut-off values: Non-AS: STAI-S⩽34, Low AS: 34<STAI-S⩽44, and High AS: STAI-S>44. We found that the generated SSCI Ⅱ values have a high correlation with the STAI-S results and can get an outstanding classification performance in the CPT scenario (Figure 4b). It should be noted that the generated SSCI2 is usually only applicable for assessing CPT stress, as different stressors have varying degrees of impact on individuals. However, due to the similarity in response weights of biomarkers to various acute stressors, this indicator shall still be used for semi-quantitative evaluation of the effects of other stressors like examinations or crowds.

Unlike AS, subjects with CS are stimulated by stressors for a longer period of time, and the metabolic pathways in the body, combined with various environmental factors such as growth

education and social support, evolve into a complex and stable superposition. Therefore, it is difficult to qualitatively and quantitatively evaluate CS using straightforward numerical models. To this end, ML models are utilized to generate the comprehensive indicator. The biomarker panel and conventional demographical characteristics (gender, age, height, weight, mean blood pressure (MBP)) are selected as the features to train the ML models. However, due to the significant intra-individual variance of cortisol and glucose, the utilization of the same individual's detection results at different times may generate models with significant differences and produce completely opposite evaluation results. It would be meaningless if the cortisol and glucose concentrations used as features are not derived from the same time period. Therefore, for practical application, we detected glucose and cortisol at any given time, and then use the Eq. (4), (8) and their built mathematical models (Eq. (5) and (9)) to retroactively calculate $Cor_{Benchmark1}$ and $Glu_{Benchmark}$ as input features for the models (In our protocol, $Cor_{Benchmark1}$ of cortisol and $Glu_{Benchmark}$ of glucose correspond to the individual's biomarker level at 8:00, $Cor_{Benchmark2}$ of cortisol should be pre-detected in advance for each subject for calculating the $Cor_{Benchmark1}$). In this way, the single input features of the model collected from different individuals can be considered as coming from the same time period (8:00). The SSCI3 can be regarded as:

$$SSCI3 = ML\{Cor_{Benchmark2}, Glu_{Benchmark1}, BT, HR, gender, age, height, weight, MBP\} \tag{13}$$

We recruited 23 depression patients and 26 healthy controls, each of whom collected data three times at any given time as input dataset for the model ($Cor_{Benchmark2}$ is only needed to be collected once). The combination results of different psychometric scales were used to label the dataset (**Note S**11). The linear regression model was used to generate a quantitative index of the depression level. Different ML classification algorithms including Random Forest (RF), Support Vector Machine (SVM), Bayes, XGBoost and Artificial Neural Network (ANN) are selected for training the classification models. RF performed best for precise recognition of depression states.

Note S11: The definition of depression state using scales

At present, the diagnosis and differentiation of psychological disorders in clinical practice still rely on self-reported psychometric tools. Numerous psychological test scales, whose development involved careful consideration of relevant literature, clinical testing and iterative refinement, have been utilized to help assess of human mental health state. These scales typically consist of several questions related to psychological state descriptors, with each question's answer suggesting a distinct numerical score. They will provide numerical results to compare with standard criteria determined by appropriate peer or normative groups, which profile the presence and severity of specific psychological disorders.

For depression assessment, several scales have been proven to have a certain value for reporting relevant information from various aspects. Widely used scales include Symptom Checklist 90 (SCL-90), Self-rating Depression Scale (SDS), Self-Rating Anxiety Scale (SAS), Hamilton Depression Rating Scale (HAMD), Kessler Psychological Distress Scale (K10), Profile and Mode states. Due to the limited and unitary information provided by each scale regarding psychological states, using a single scale to qualitatively or even quantitatively assess the stress state of the human body may not be accurate enough and may lead to misdiagnosis. Instead, the joint evaluation of multiple scales can achieve a more comprehensive analysis of the depression state. However, some scales like K10 provide insufficient information and are almost covered by other scales with higher complexity. In addition, some scales like HAMD didn't show a satisfactory performance in the analysis of depression in our preliminary experiments. These scales were excluded from subsequent experimental applications. These being so, we selected SCL-90, SDS, and SAS to offer a joint assessment result for depression profiling.

SCL-90 is a 90-item questionnaire that covers the topics of somatization, obsessive-compulsive, interpersonal sensibility, depression, anxiety, anger-hostility, phobic-anxiety, paranoid ideation, psychoticism and additional items. Each item has 5 optional tags with different scores (0: not at all; 1: a little bit; 2: moderately; 3: quiet a lot; 4: extremely). We combine the scores of depression, anxiety, and psychoticism groups as the final SCL-90 output ($F_{SCL\text{-}90}$):

$$F_{SCL-90} = \frac{\sum S_{ij}}{33} (14)$$

S_{ij} represents the score for the j_{th} item of the i_{th} topic (i = depression, anxiety and psychoticism). 33 means the number of items belonging to the above three topics. SDS is a 20-item

questionnaire that is designed to measure the affective, psychological and somatic aspects of depression. Each item is assigned a rating on a scale from 1 to 4 (1: a little of the time; 2: some of the time; 3: good part of the time; 4: most of the time. Some item's scores are counted in reverse). The final score of SDS is 1.25 times the sum of the scores for all items:

$$F_{SDS} = \sum_{i=1}^{20} S_i (15)$$

S_i represents the score for the i_{th} item. SAS is somewhat similar to SDS and it consists of 20 items used to quantify the anxiety state of participants from various dimensions including cognitive, affective, somatic, and autonomic symptoms. The rating criteria of SAS are same with SDS and the score can be calculated as follows:

$$F_{SAS} = \sum_{i=1}^{20} S_i (16)$$

In order to balance the impact of the scores of the three scales, we ultimately defined the overall depression scale results as follows:

$$F = 10F_{SCL-90} + F_{SDS} + F_{SAS} (17)$$

The overall depression score is from 50 to 250, and we define the state of depression into four categories based on the overall score of the scale: Non depression patients (ND): $F<105$; People with depression risk (DR): $105 \leqslant F < 135$; Mild depression patients (MD): $135 \leqslant F < 165$; Severe depression patients: $F>165$.

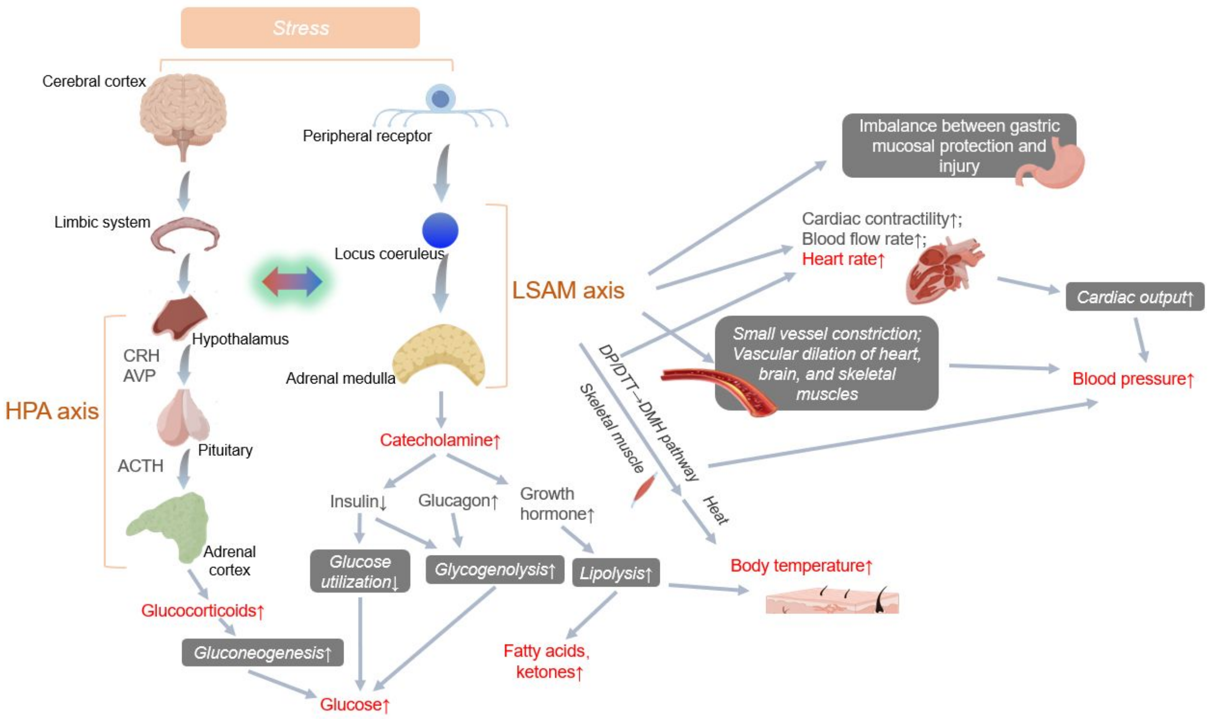

Figure S1. Physiological responses to stress stimulations. The dominant physiological responses involve two axes: HPA and LSAM axis. The secretions of glucocorticoids and catecholamine are promoted by the activation of these axes, which further regulate glucose metabolism. Other physiological activities will also respond to stress stimuli along or immediately after these two pathways, typically including alterations in vessel state, temperature regulation, cardiac output and immune balance. HPA: Hypothalamic-Pituitary-Adrenal cortex; LSAM: Locus coeruleus-Sympathetic-Adrenal-Medulla; CRH: corticotropin-releasing hormone; AVP: arginine vasopressin; ACTH: adrenocorticotropic hormone; DP: dorsal peduncular; DTT: dorsal tenia tecta; DMH: dorsomedial hypothalamus. (Created by figdraw.com)

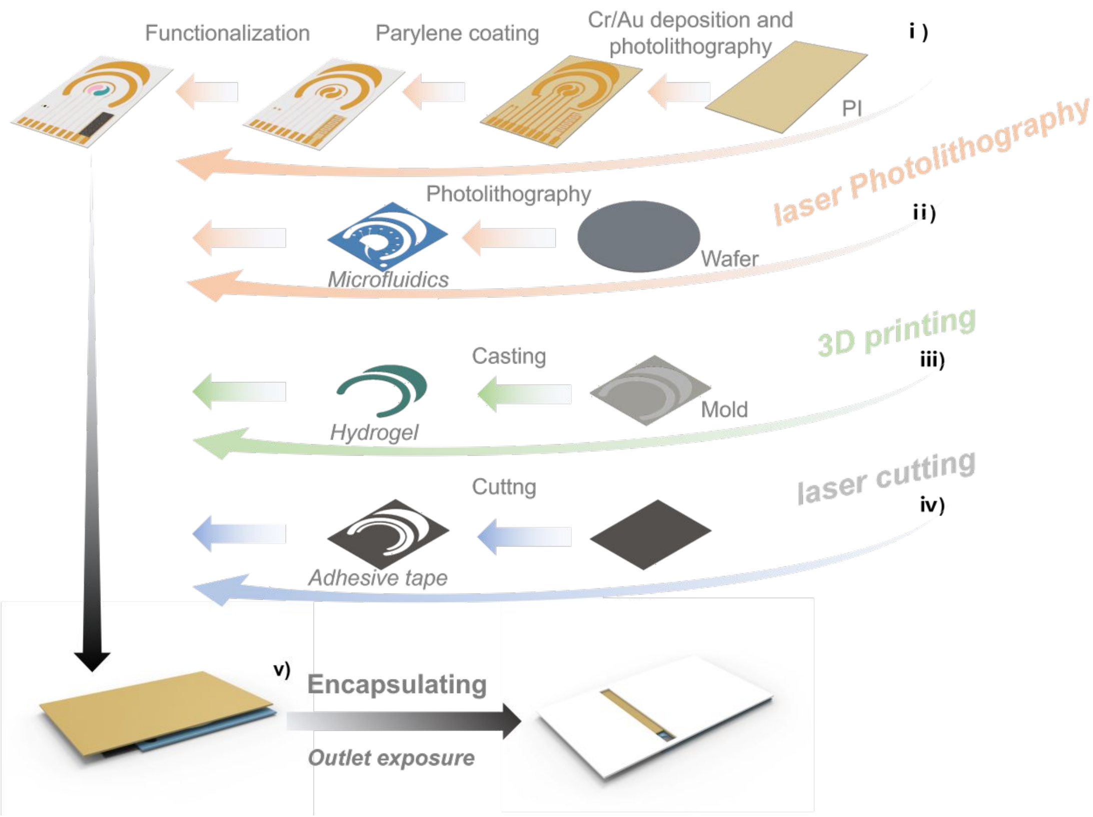

Figure S2. Fabrication process illustration of the integrated sensing system. ⅰ) sensing array fabrication including Cr/Au deposition and photolithography, parylene encapsulating layer coating and functionalization. ⅱ) microfluidic module fabrication using SU8-2050 and PDMS by photolithography. ⅲ) Agarose hydrogel fabrication. ⅳ) adhesive layer with specific pattern fabrication. v) sweat evaporation outlet cutting and device encapsulating with Ecoflex after integrating all the functional components.

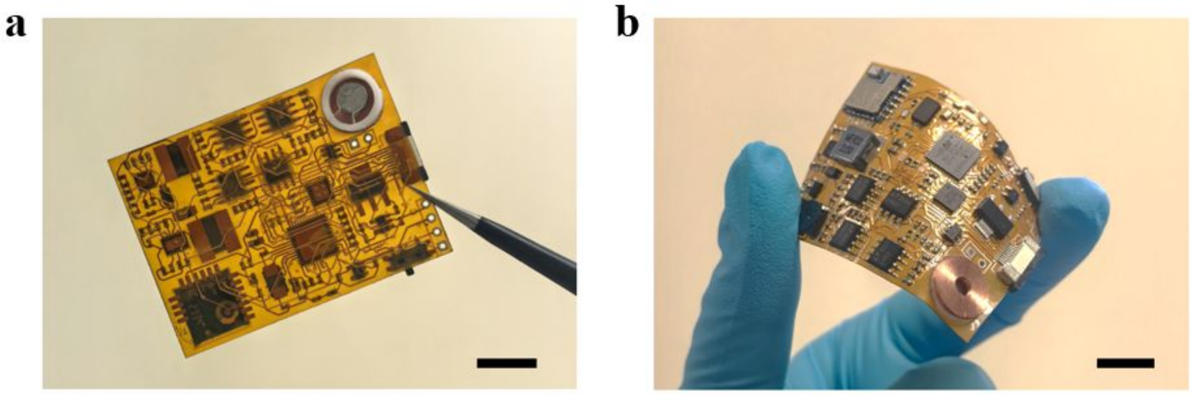

Figure S3. Optical images of the FPCB. a Top view of the back. **b** FPCB at a bending state. Scale bar: 1 cm.

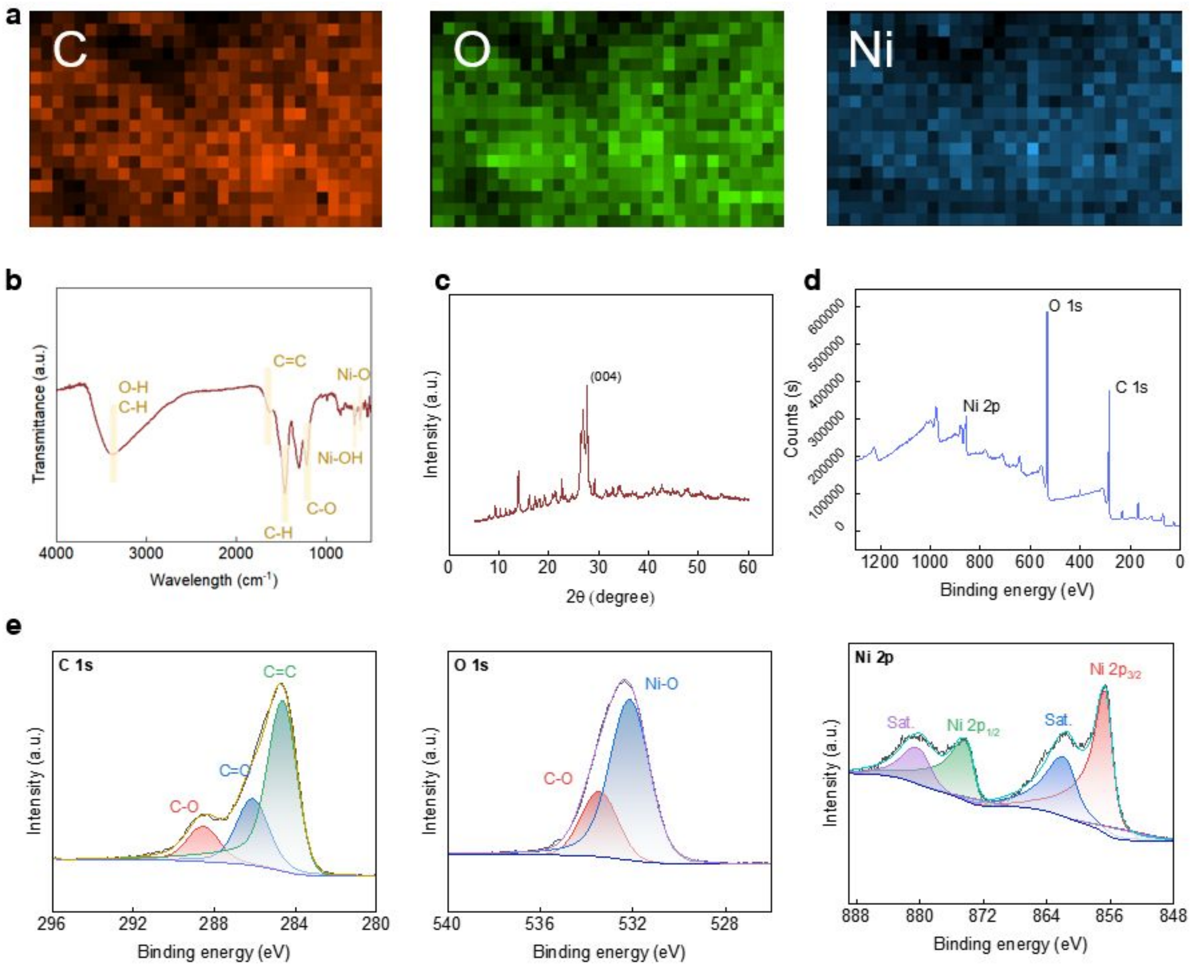

Figure S4. Characterization of NiCAT. **a** EDS mapping; **b** FTIR; **c** XRD; **d** XPS survey and **e** XPS spectra for C 1s, O 1s and Ni 2p of NiCAT. The element of C, O, Ni can be observed with a uniform distribution on the electrode surface. The FTIR spectrum reveals characteristic absorption peaks at 662°, 721°, 1214°, 1447°, 1640°, 3409°, attributed to Ni-O, Ni-OH, C-O, C-H, C=C, and C-H/O-H stretching vibrations. The XRD pattern exhibits a distinct diffraction peak at 2θ=26.8°, corresponding to the (004) plane. The C 1s, O 1s and Ni 2p peaks can be observed by the XPS. The C 1s spectrum shows the fitting peaks at 284.6, 286.2, and 288.5 eV, corresponding to the C-O, C=O, and C=C bonds. The O 1s spectrum shows the fitting peaks at 532.1, and 533.4 eV, which can be attributed to Ni-O, and C-O of the coordinated catecholates. The Ni $2p_{1/2}$ and $2p_{3/2}$ peaks are observed in the Ni 2p spectrum at 874.4, and 856.6 eV with their satellite peaks at 861.9, and 880.5 eV.

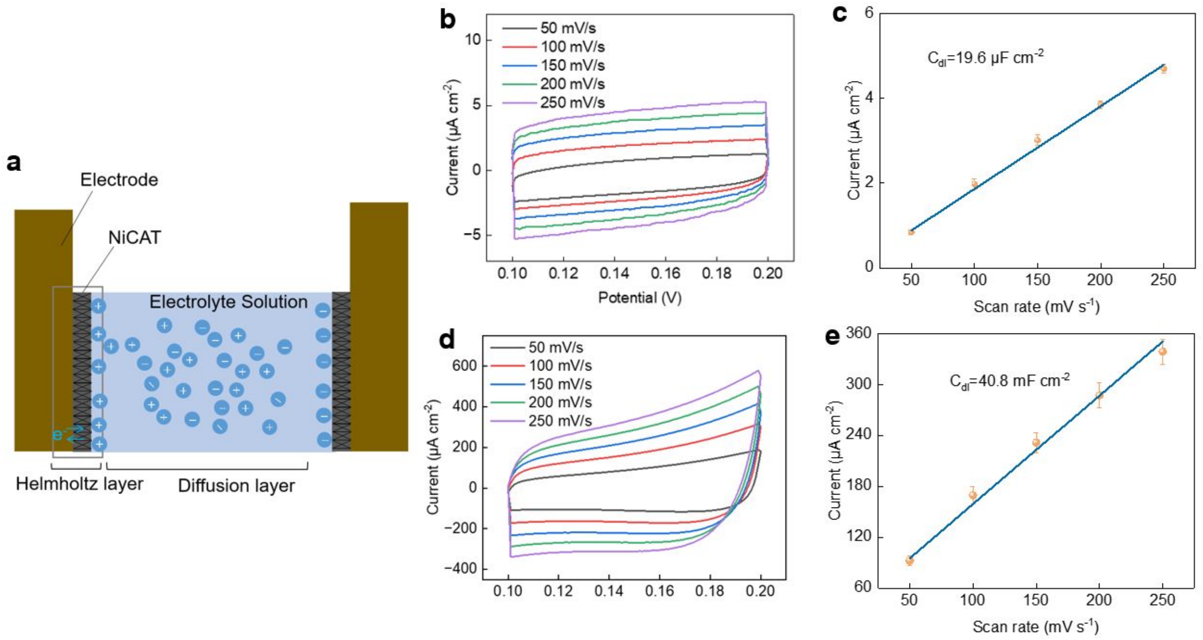

Figure S5. Characterization of double-layer capacitance of NiCAT-modified electrodes. **a** Schematic of the electric signal transmission between electrodes in the electrochemical system. **b** CV plots of Cr/Au electrodes at different scan rates. **c** Linear correlation between current density and scan rate of Cr/Au electrodes. **d** CV plots of Cr/Au@NiCAT electrodes at different scan rates. **e** Linear correlation between current density and scan rate of Cr/Au@NiCAT electrodes.

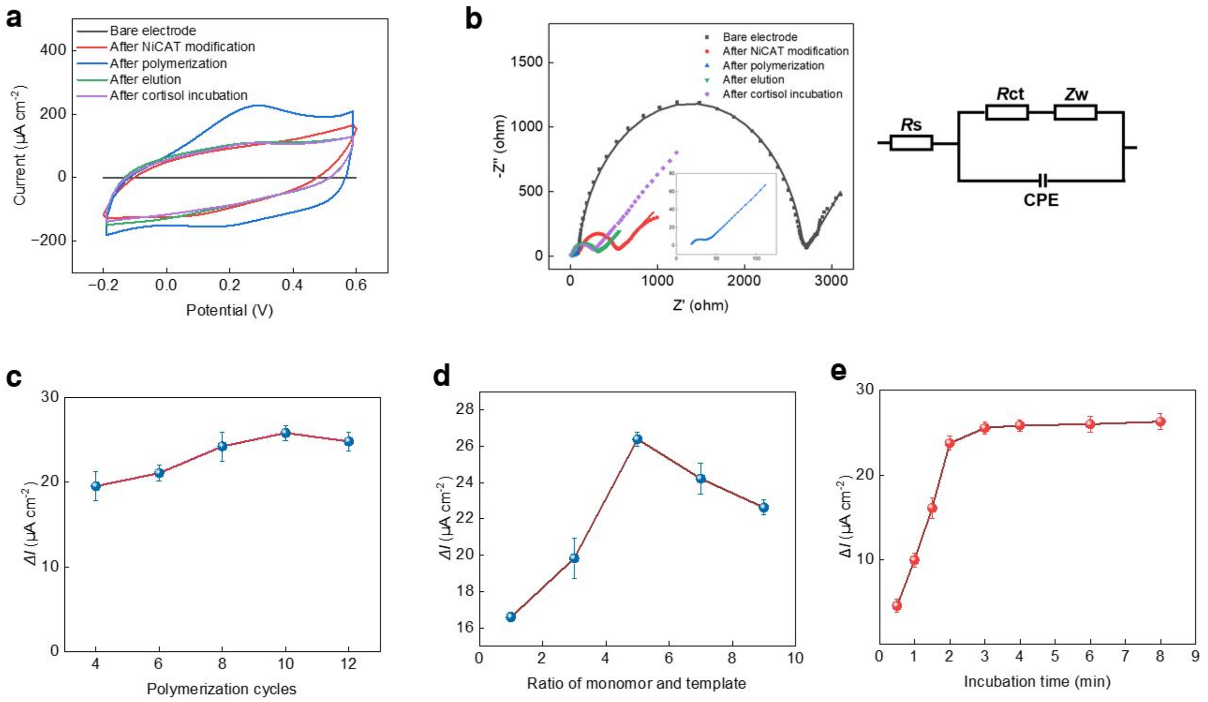

Figure S6. Fabrication of cortisol sensors. a CV characterization of the preparation process of cortisol sensors. **b** EIS characterization of the preparation process of cortisol sensors. The right panel shows the equivalent circuit. **(c** and **d)** The signal variances towards 500 nM cortisol of the cortisol sensors fabricated with different polymerization cycles (**c**) and ratio of monomer and template (**d**). **e** The signal variances of the cortisol sensor after being incubated with 500 nM cortisol for different times.

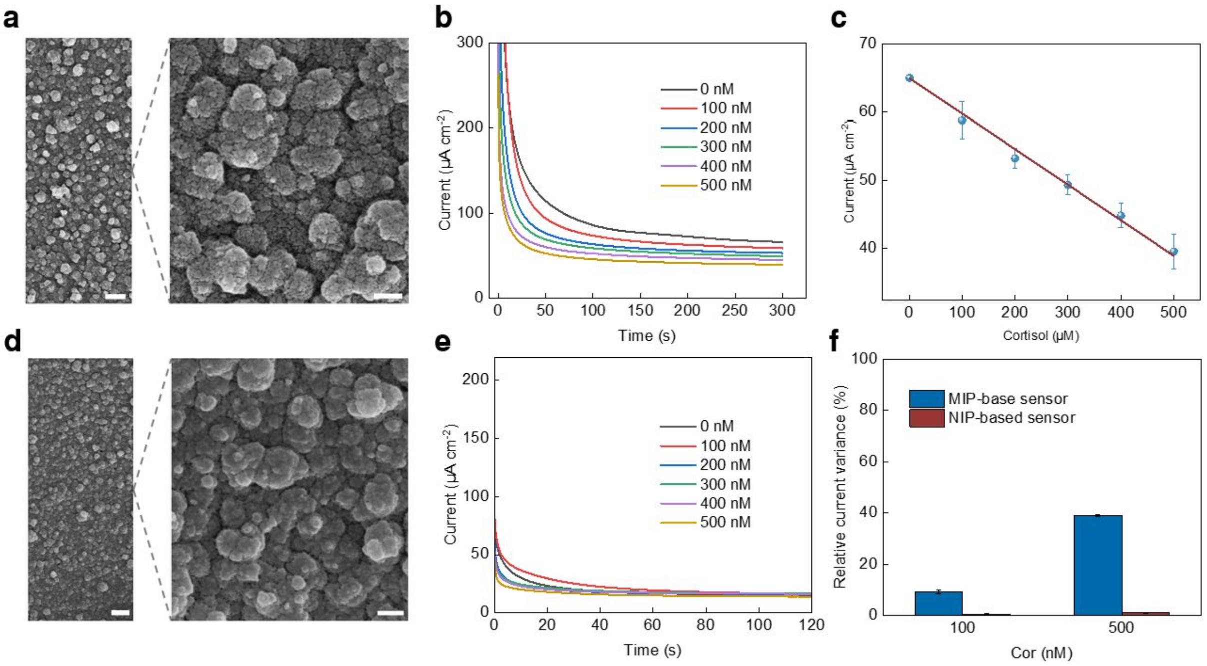

Figure S7. Sensing performance of MIP and NIP-based electrodes. **a** SEM images of MIP-based sensors. Scale bar: 5 μm. **b** Chronoamperometric response of the MIP-based sensors to different cortisol concentrations. **c** the corresponding calibration plot of the cortisol sensor responses. **d** SEM images of NIP-based sensors. Scale bar: 5 μm. **e** Chronoamperometric responses of the NIP-based sensors to different cortisol concentrations. **f** Relative current variances of the MIP and NIP-based sensor towards different cortisol concentrations in PBS (1×) solution.

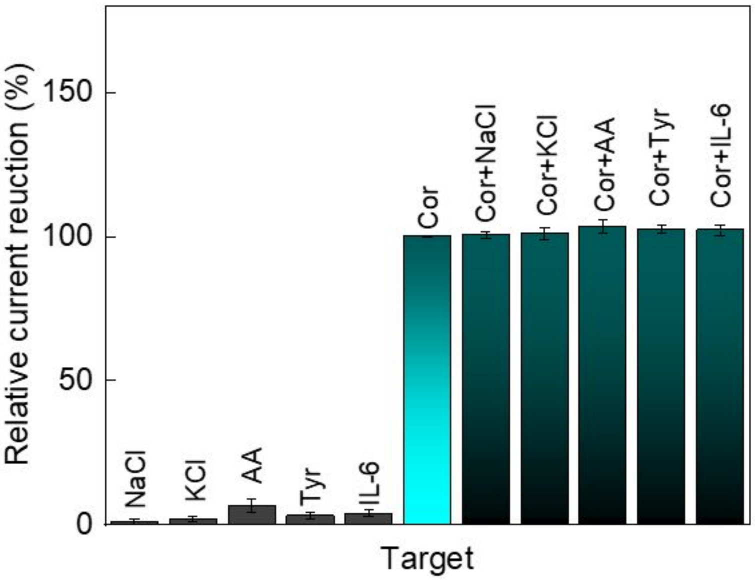

Figure S8. Selectivity and anti-interference capability characterization of the cortisol sensor. AA: ascorbic acid; Tyr: tyrosine; IL-6: interleukin-6; Cor: cortisol.

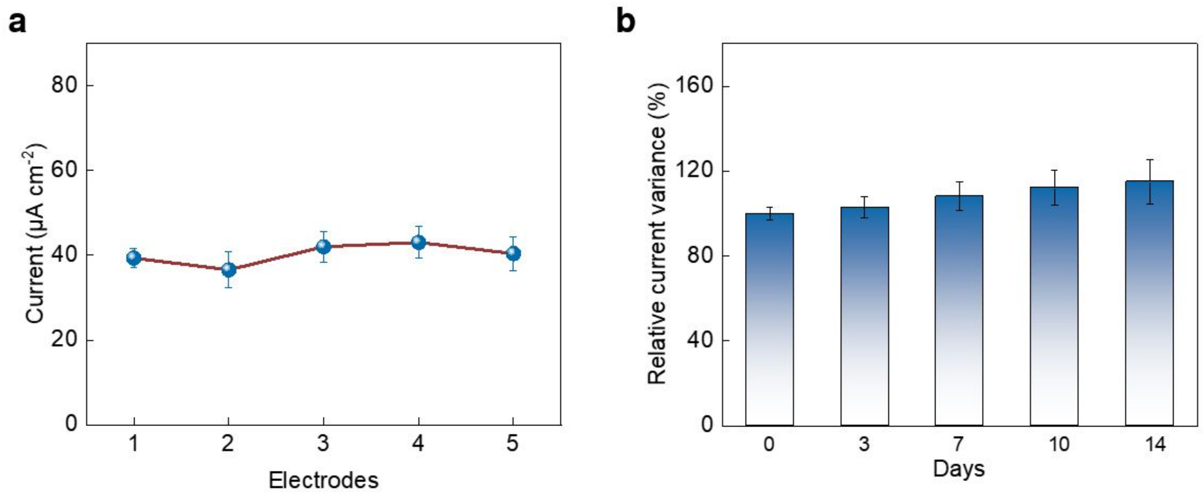

Figure S9. Reproducibility and stability of the cortisol electrode. a The signal responses of different cortisol sensors to 500 nM cortisol. **b** The relative signal variances of the cortisol sensors with different storage time periods.

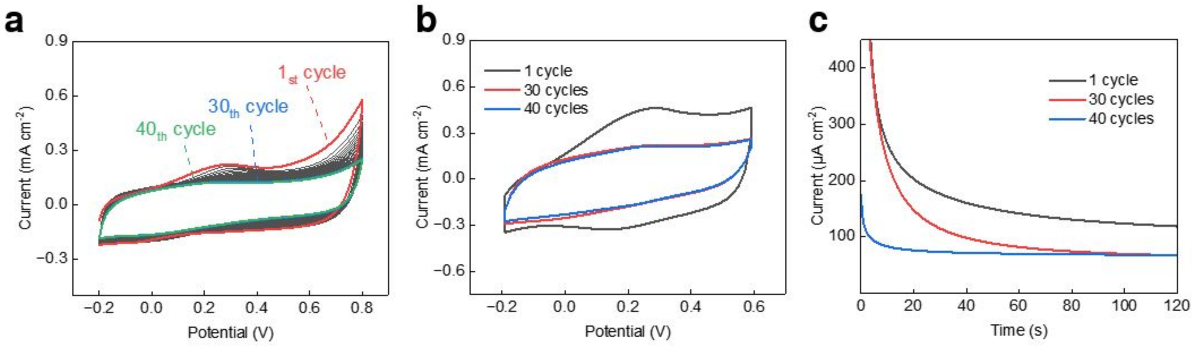

Figure S10. Characterization of the template removal process. **a** The CV plots of the electrical elution process with different scan cycles. **b** The CV plots of the cortisol sensor prepared with 1, 30 and 40 electrical elution cycles in PBS solution. **c** The i-t plots of the cortisol sensor prepared with 1, 30 and 40 electrical elution cycles in PBS solution.

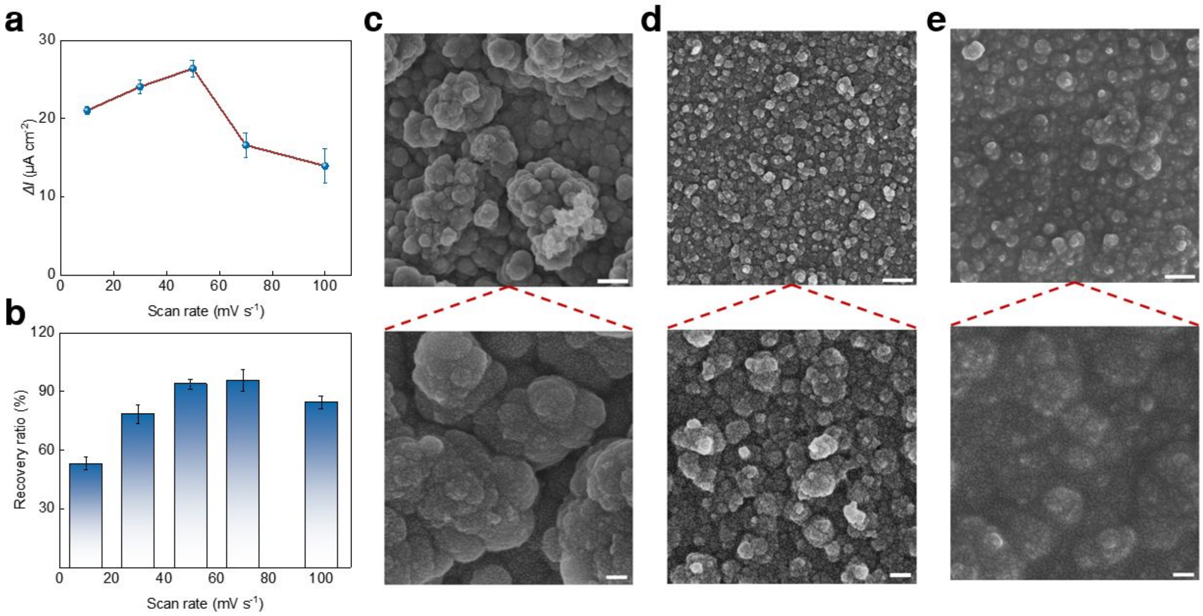

Figure S11. The influences of MIP fabrication on the regeneration effect. **a** The signal variances towards 500 nM cortisol of the cortisol sensors fabricated with different scan rates. **b** The signal recovery ratios of the cortisol sensors fabricated with different scan rates. **(c-e)** SEM images of cortisol sensors fabricated with the scan rate of 10 mV s^{-1} (**c**), 50 mV s^{-1} (**d**) and 100 mV s^{-1} (**e**). Scale bar: 1 μm (Upper) and 200 nm (Lower).

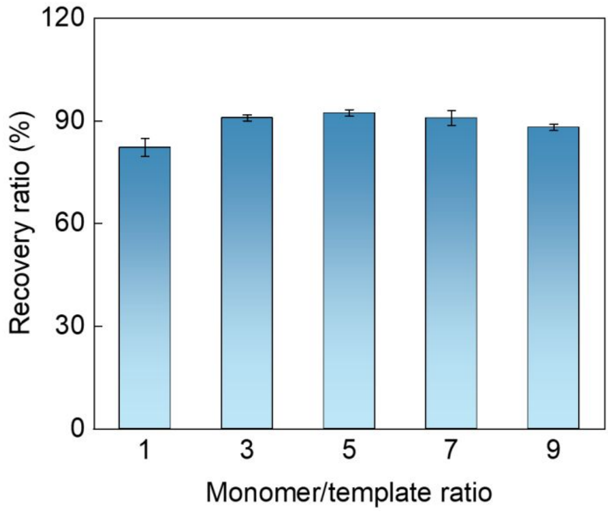

Figure S12. The influence of monomer/template ratio on the regeneration effect.

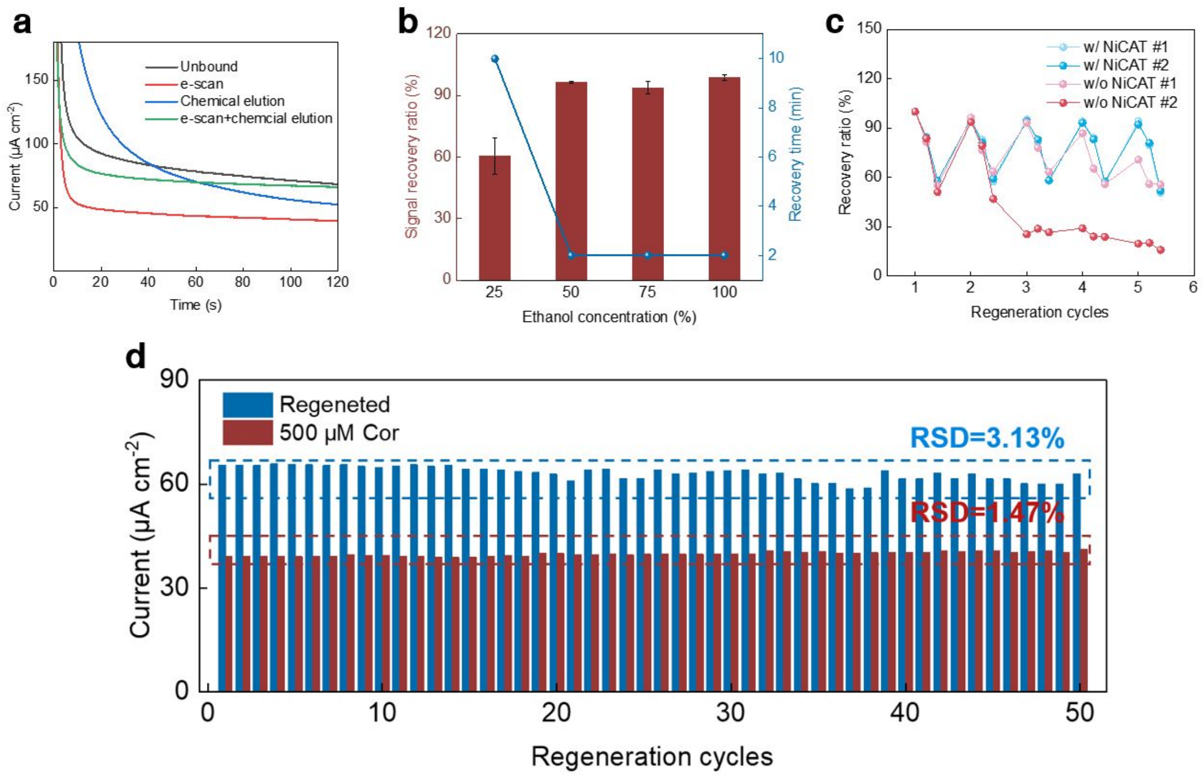

Figure S13. Characterization of the regeneration performance. a The regeneration signals with different approaches. **b** The regeneration performances using ethanol with different concentrations. **c** The regeneration performances of cortisol sensors with and without the NiCAT modification. **d** The signals of the cortisol sensor during 50 cycles of regeneration.

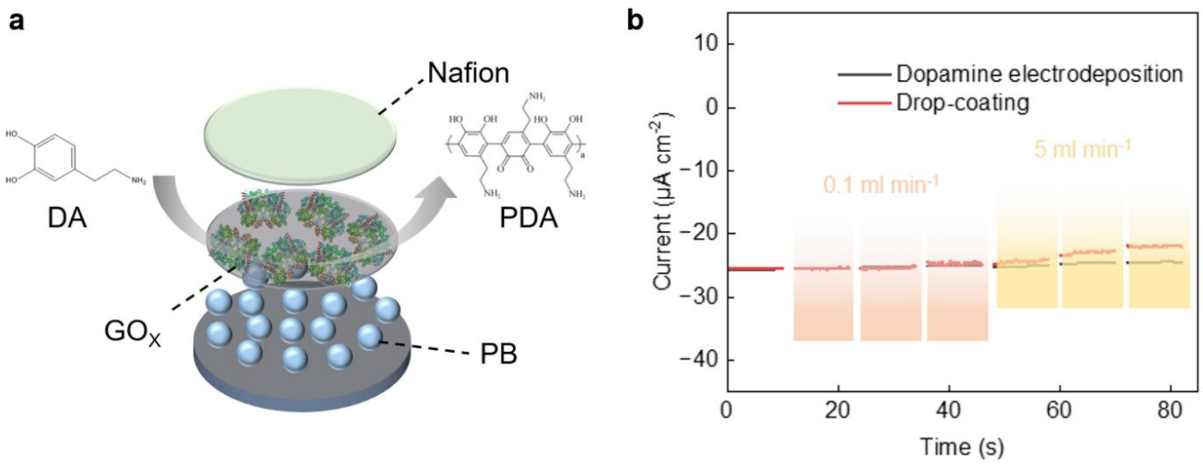

Figure S14. Interface stability of glucose electrodes prepared by different methods. a The schematic illustration of the glucose sensor's construction. **b** The detection signals of glucose sensors prepared with different methods during repeated rinse operations. Glucose concentration: 200 μM.

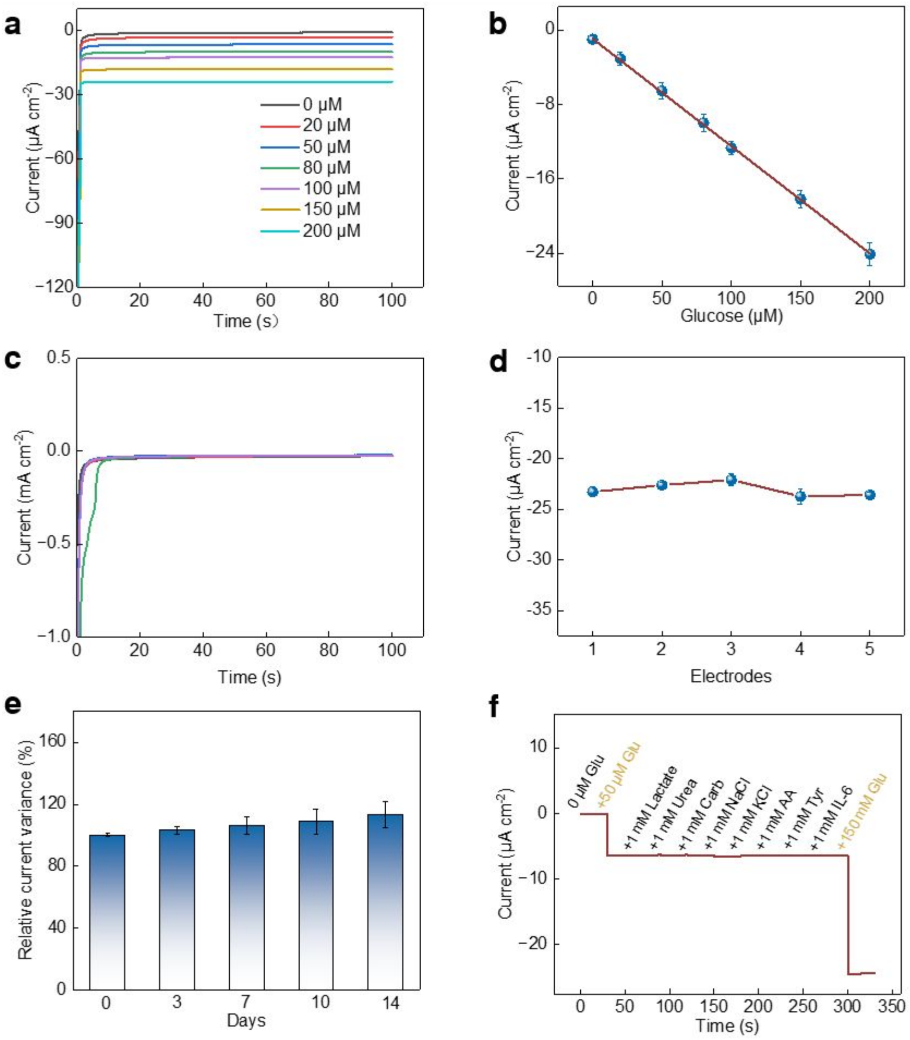

Figure S15. Methodological characterization of glucose electrodes. a The chronoamperometric responses of the glucose sensors to different glucose concentrations. **b** The corresponding calibration plot of the glucose sensor responses. **c** The chronoamperometric plots of five different glucose sensors towards 200 μM glucose. **d** The measured density current of five different glucose sensors towards 200 μM glucose. **e** Long-term stability of the glucose sensors. **f** Selectivity of the glucose sensors to potential interference in human sweat and the used sweat-induction drug. Glu: glucose; Carb: carbachol; AA: ascorbic acid; Tyr: tyrosine; IL-6: interleukin-6.

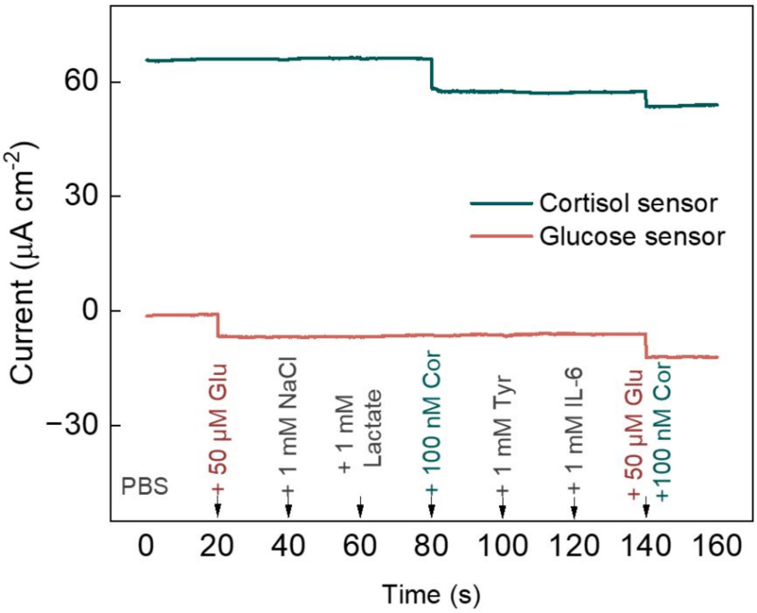

Figure S16. Co-sensing crosstalk characterization of the sweat sensors. Glu: glucose; Tyr: Tyrosine; Cor: Cortisol.

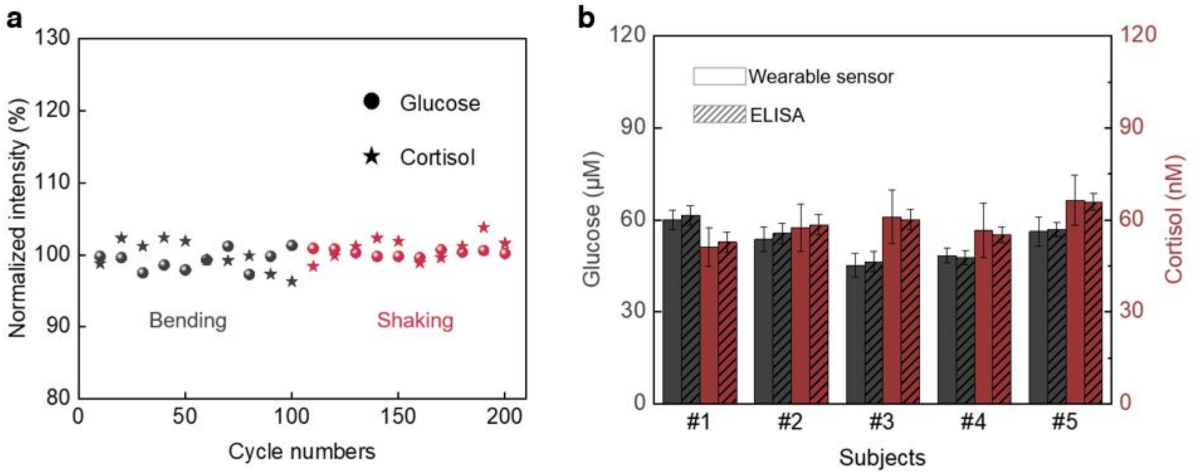

Figure S17. The on-body detection performances of sweat sensor array. **a** The normalized signal intensity of sweat sensors under different operations. The bending and shaking testing were conducted in a sequential manner. **b** Validation of the sweat sensor with commercial ELISA kits using sweat samples collected from five subjects.

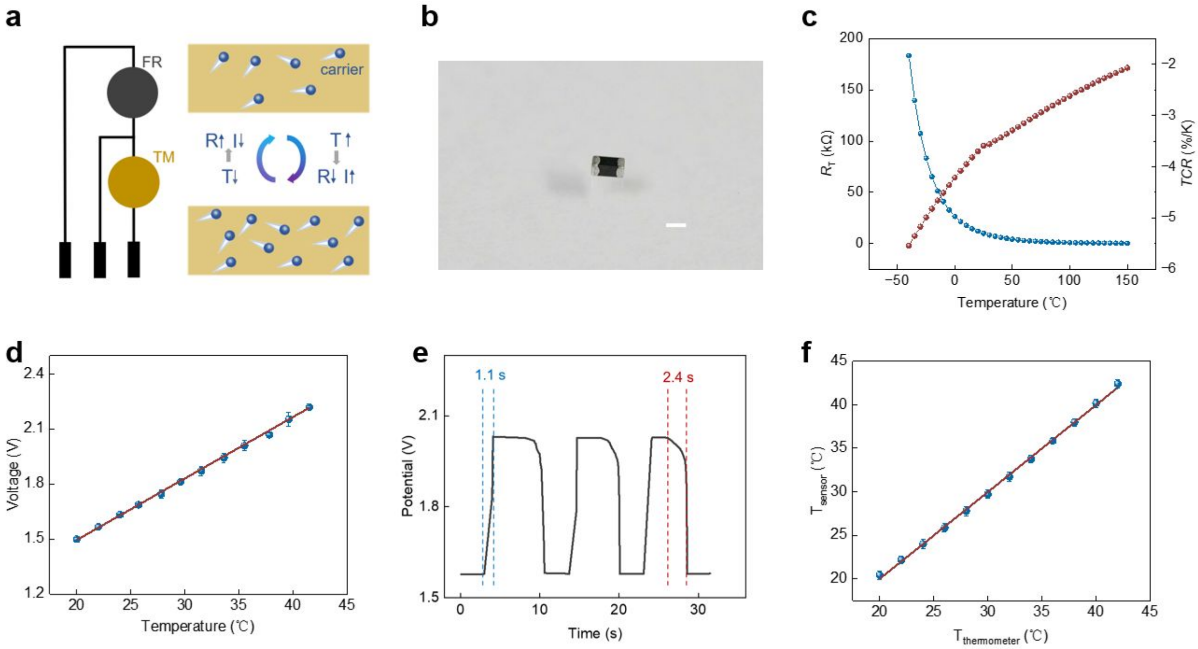

Figure S18. Characterization of the temperature sensor. **a** The detection principle of the thermistor. FR: fixed resistor; TM: thermistor. **b** The optical image of the thermistor. Scale bar: 1 mm. **c** The temperature-sensitive characteristics of the thermistor. TCR: Temperature Coefficient of Resistance. **d** The calibration plot of the temperature sensors. **e** The signals of the temperature sensors when temperature rises and falls. **f** Comparison of detection results between the temperature sensor and the mercury thermometer.

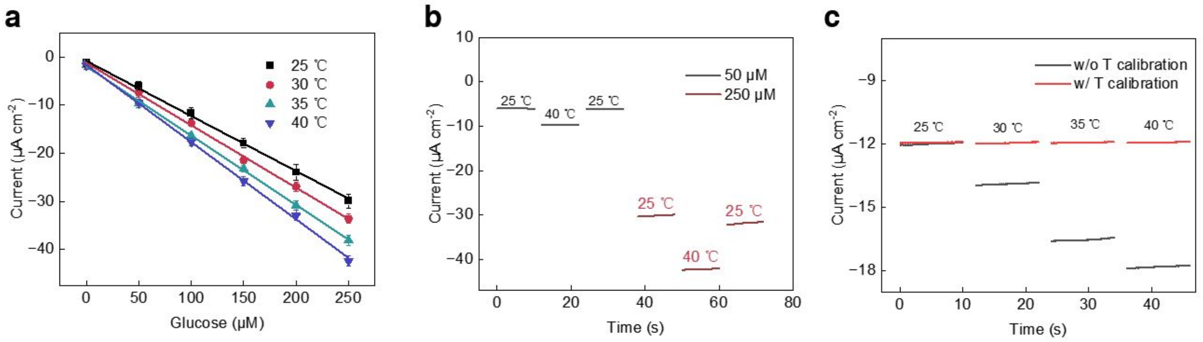

Figure S19. Compensation of glucose sensors. **a** The calibration curve of glucose sensors under different temperatures. **b** the influence of temperature on glucose detection signals. **c** Signal responses of glucose sensors towards 100 μM glucose with and without temperature calibration.

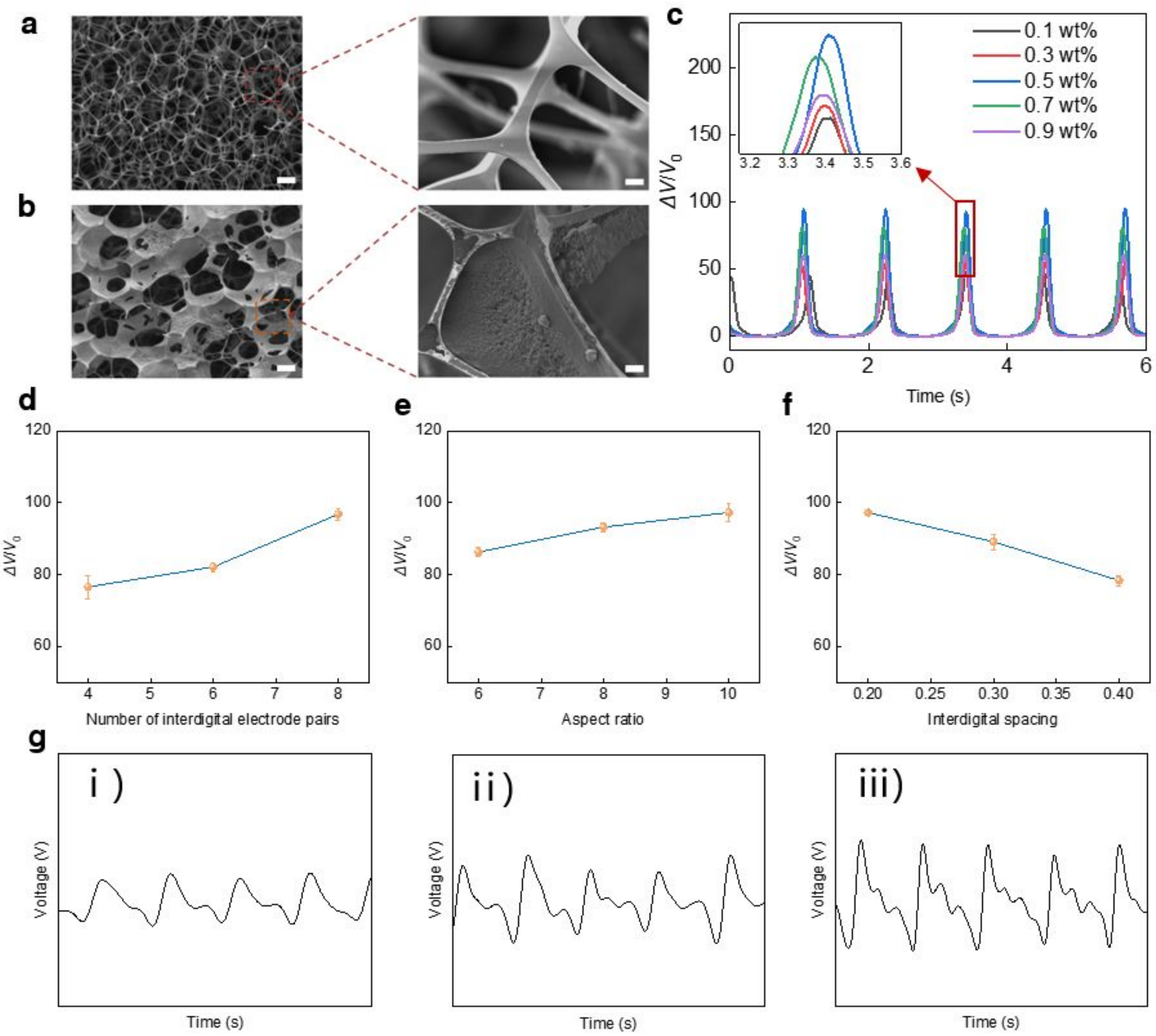

Figure S20. Pressure-sensitive characteristics of the sponge-based sensor. **(a** and **b)** SEM images of the sponge before (**a**) and after (**b**) mixing with CNT/CB. Scale bar: 500 μm (Left) and 10 μm (Right). **c** The signal responses of the HR sensors fabricated with different amounts of CNT/CB. **(d-f)** The influence of interdigital electrode pair numbers (**d**), aspect ratio (**e**), and interdigital spacing (**f**) of the interdigital electrode on the sensing sensitivity. **g** Pulse signal with different sensing precision.

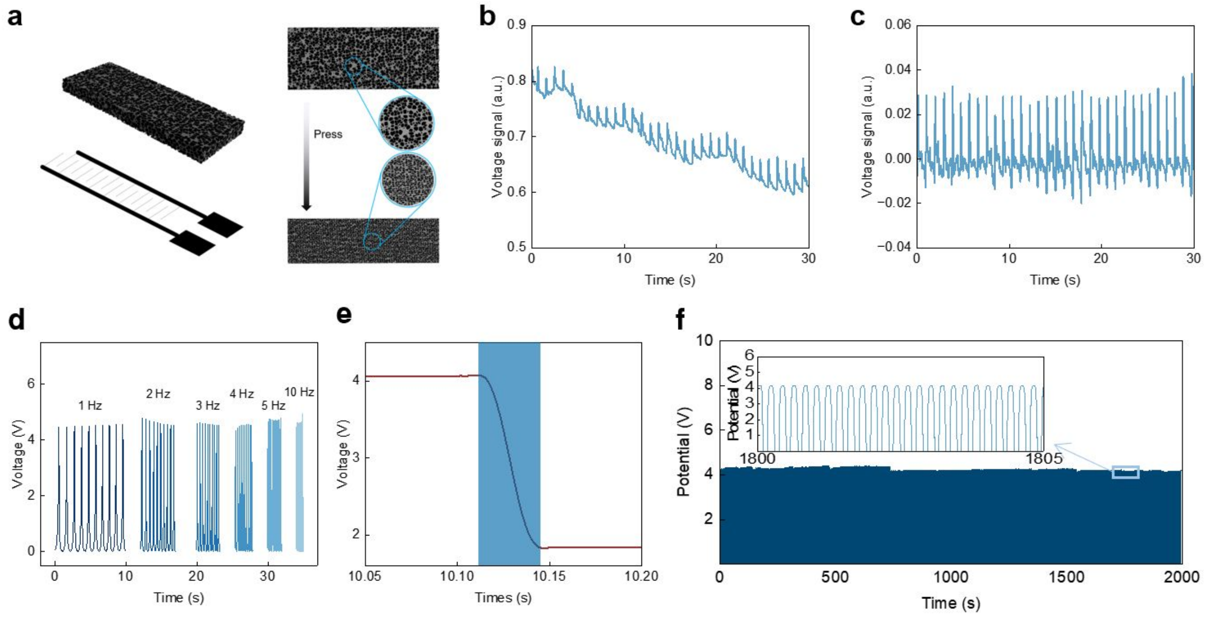

Figure S21. Characterization of the piezoresistive sensor for HR detection. **a** Construction and detection principle illustration of the HR sensor. **(b** and **c)** Pulse signal before (**b**) and after (**c**) band-pass filtering. **d** The signals of the HR sensors under different press frequencies. **e** The signal response when switching an applied pressure. The response time is about 34 ms. **f** The signal response of the sponge-based HR sensor within 10000 cycles press. Inset shows the signal within a short time period.

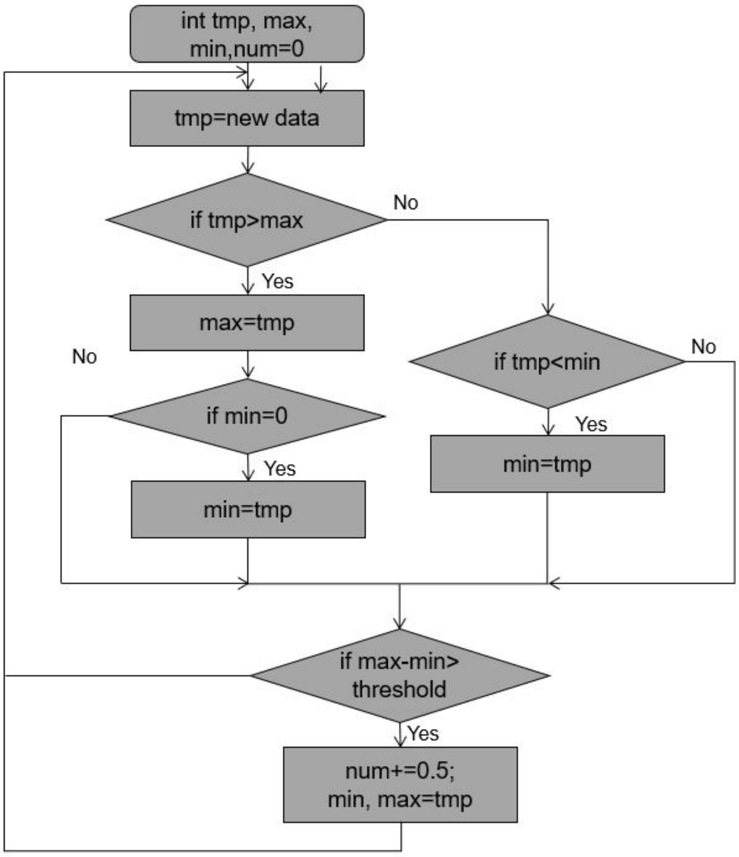

Figure S22. Workflow of HR value acquisition.

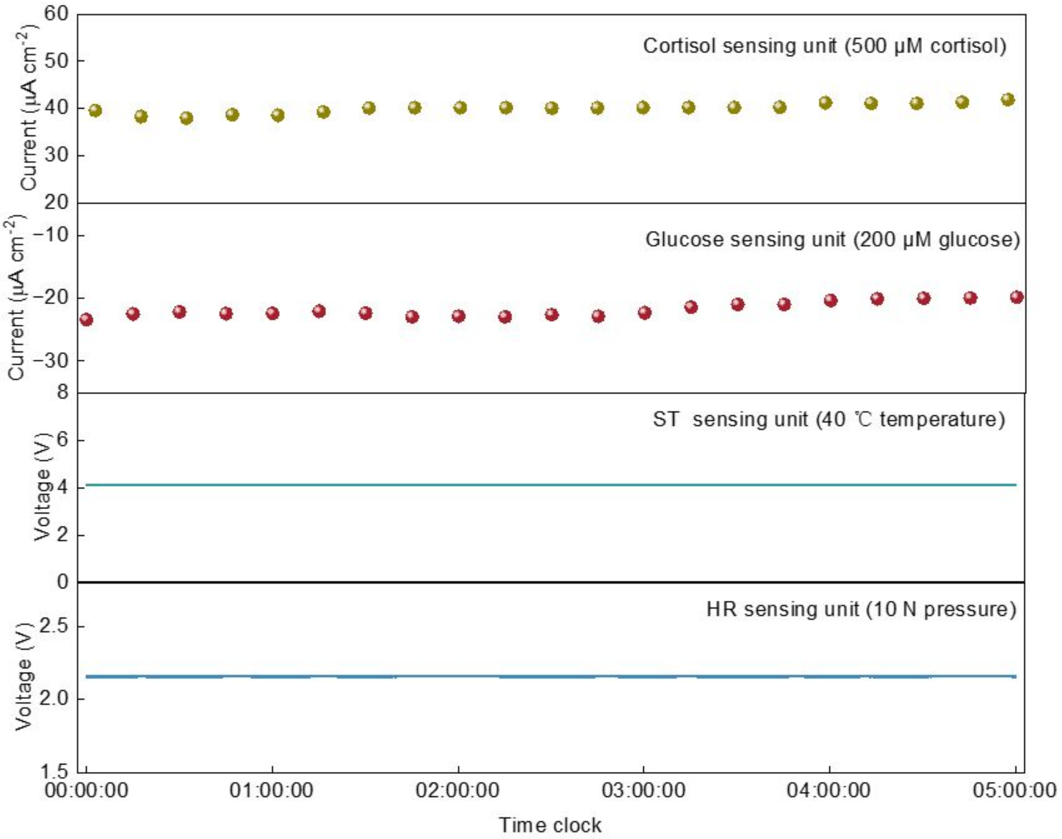

Figure S23. Sensing performance under repeated usage.

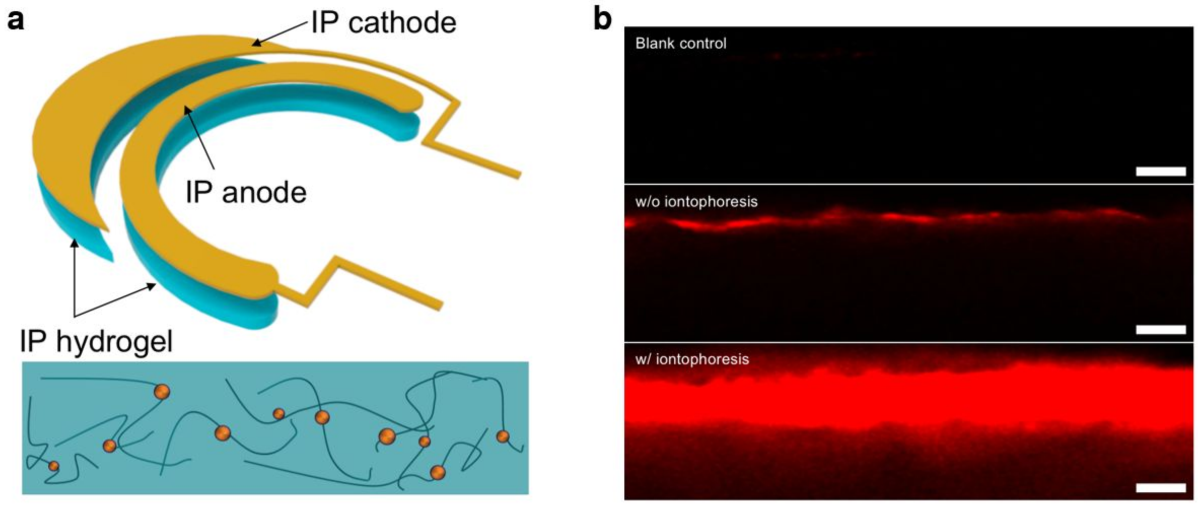

Figure S24. Characterization of drug release using the iontophoresis module. **a** The schematic of iontophoresis electrodes and drug-loaded hydrogel. **b** Fluorescence characterization of the iontophoresis effect on the pig skin using R6G as the drug.

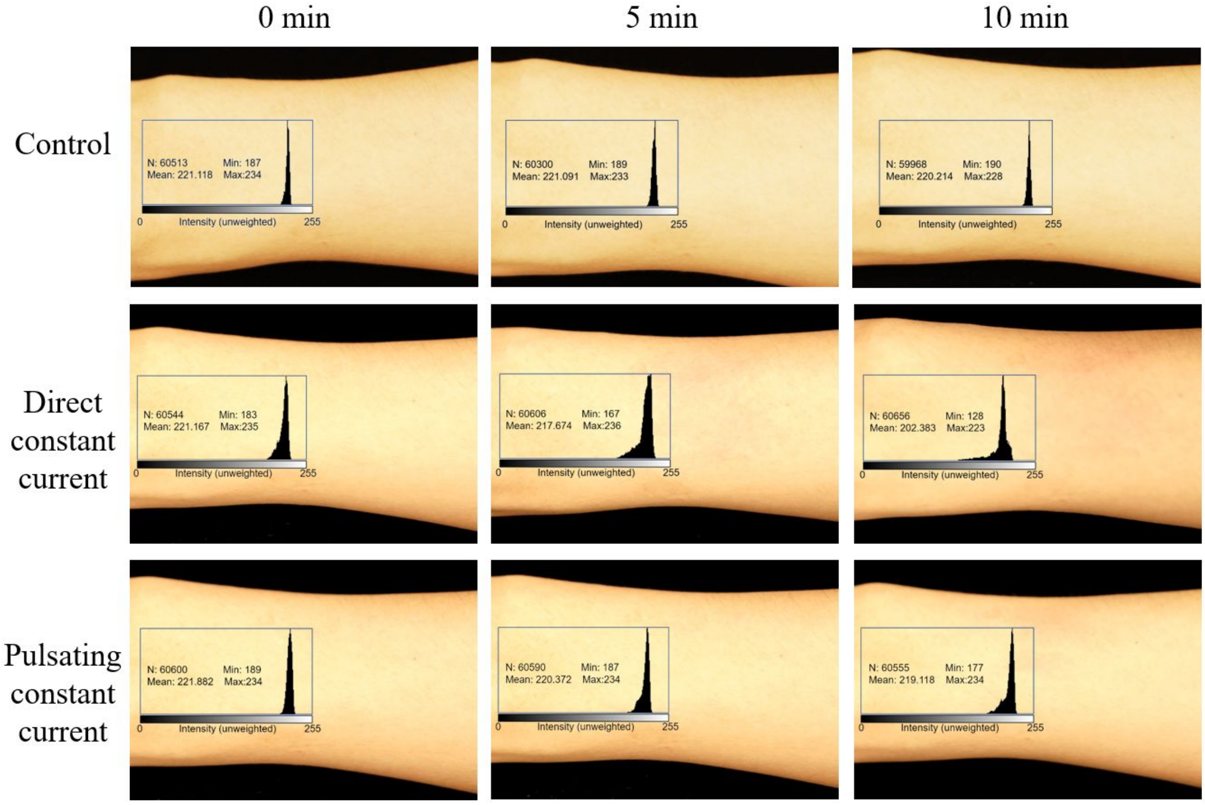

Figure S25. Skin dynamics during iontophoresis process under different stimulation modalities.

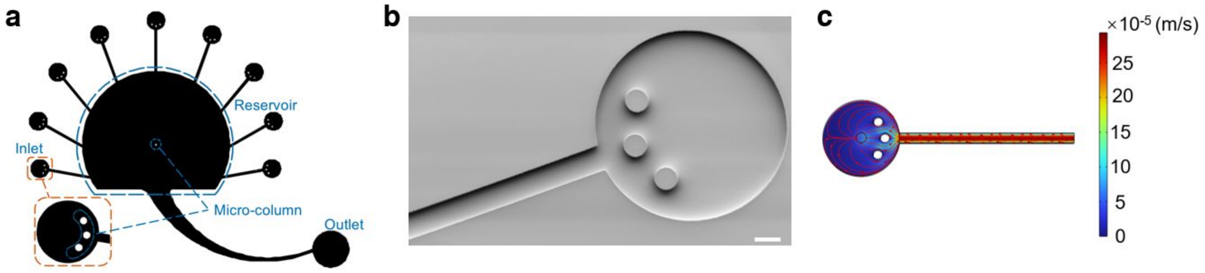

Figure S26. Characterization of the micro-columns in the inlet. **a** Schematic illustration of the microfluidic pattern. **b** SEM image of the micro-columns. Scale bar: 200 μm. **c** The finite element analysis simulation result of sweat flow with the presence of micro-columns.

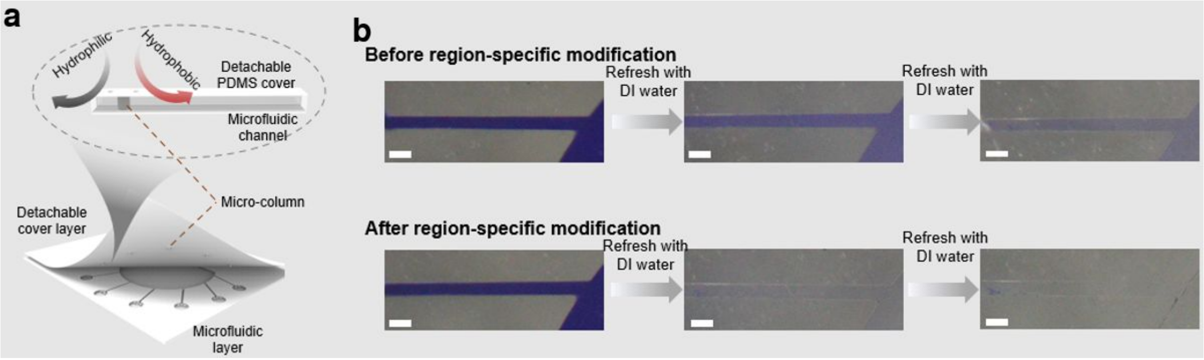

Figure S27. Optimization of the channel interface. a Schematic illustration of microfluidic channel interface optimization approach. **b** Optical images of the microfluidic channel which are filled in blue ink and refreshed by DI water without (Upper) and with (Lower) channel interface optimization.

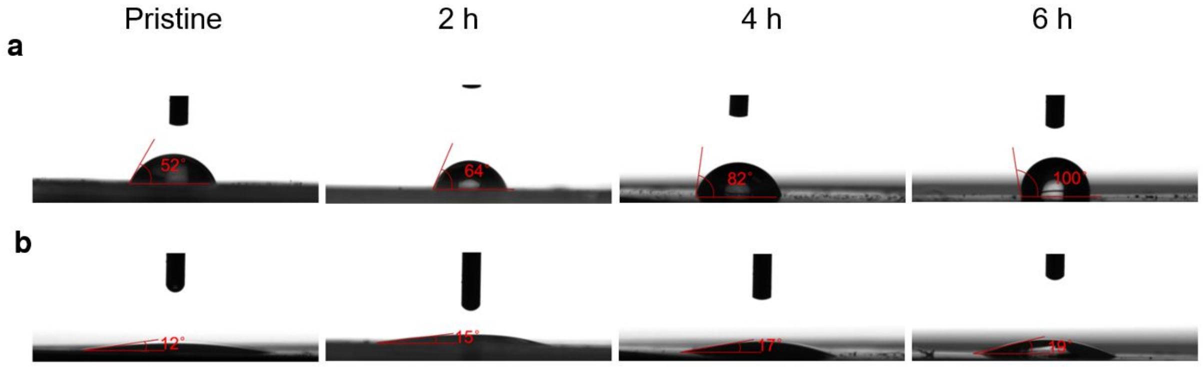

Figure S28. Long-term interface hydrophily of the microfluidic channel. **(a** and **b)** The contact angle of PDMS@plasma (**a**) and PDMS@plasma/SDS (**b**) at different time after hydrophilic treatment.

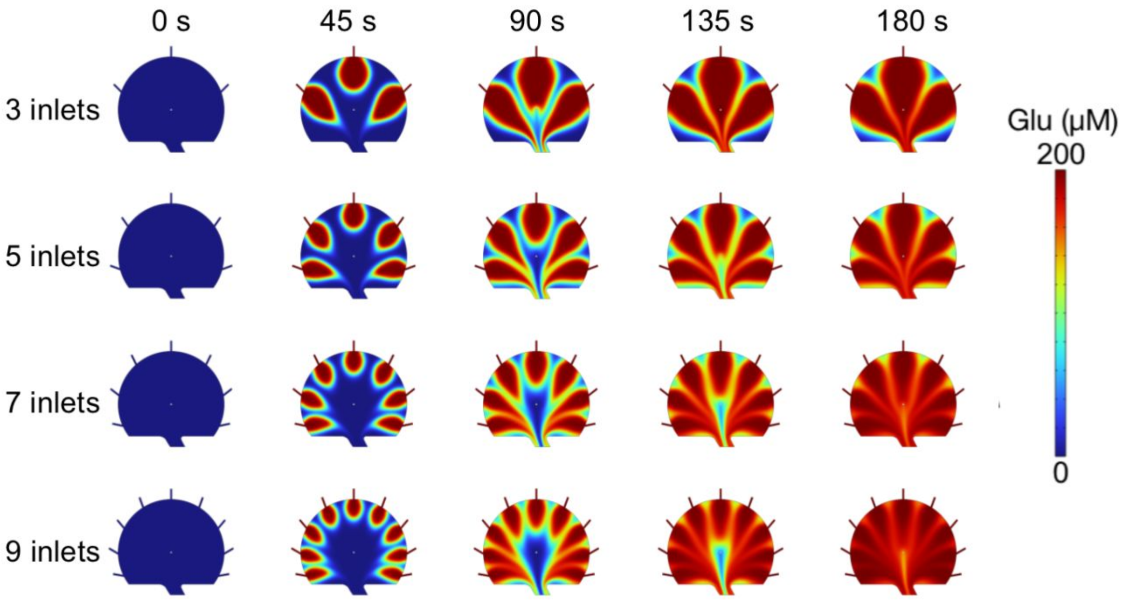

Figure S29. Effects of microfluidic inlet numbers on the sweat flow.

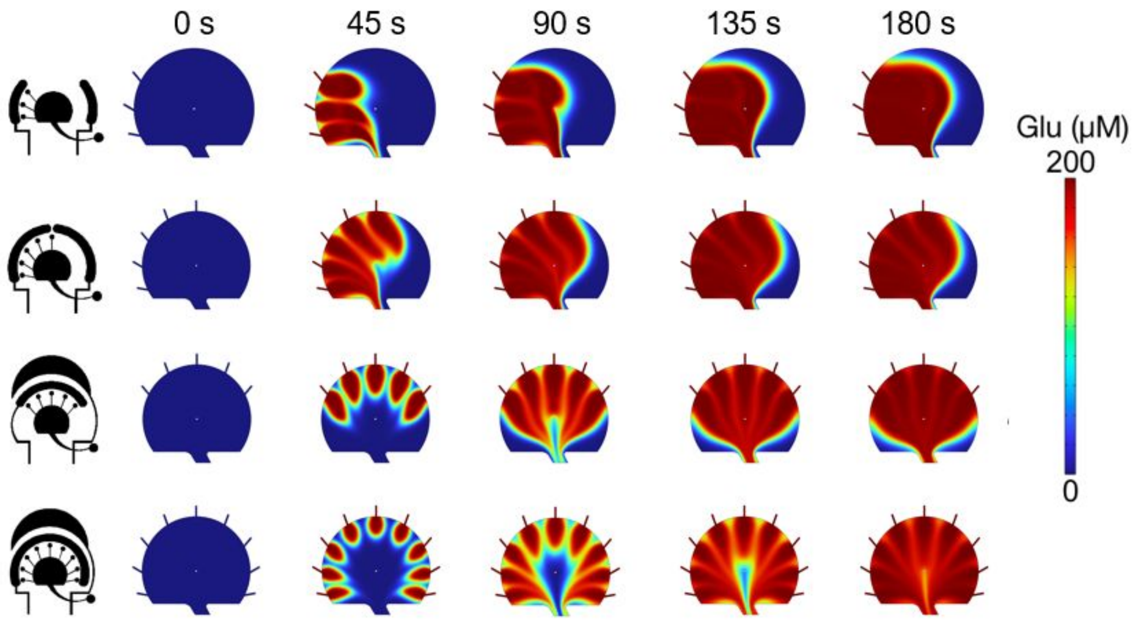

Figure S30. Effects of iontophoresis electrode and microfluidic patterns on the sweat flow.

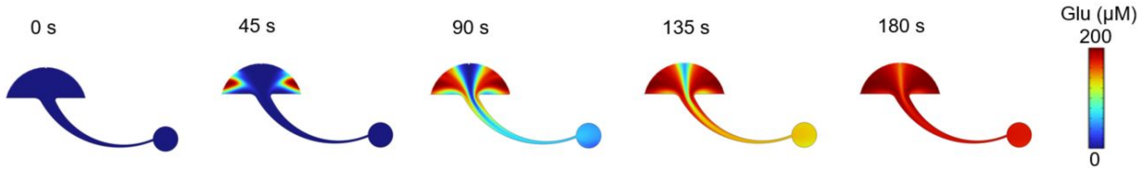

Figure S31. Simulation characterization of sweat outflow at microfluidic outlet.

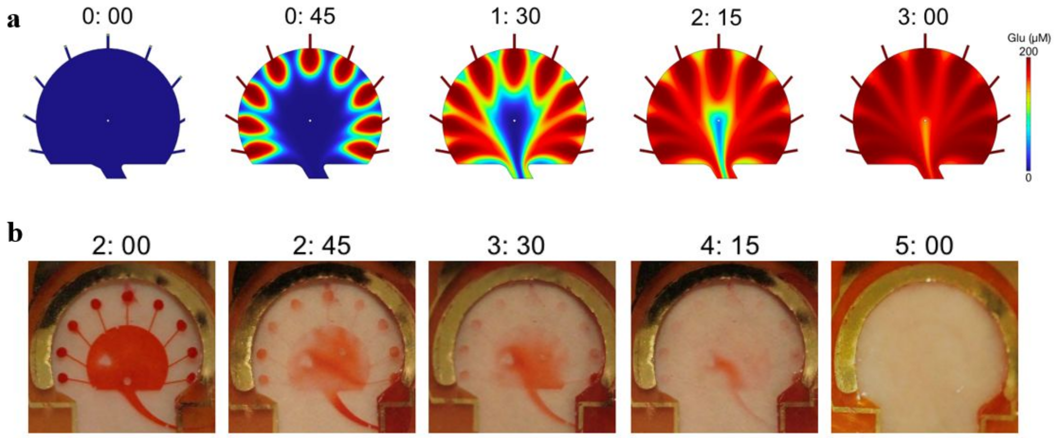

Figure S32. Characterization of on-body sweat induction process. **a** Numerically simulated glucose concentration distribution at different times after 200 μM glucose inflow. **b** Optical images of the microfluidics during the iontophoresis period. The microfluidic layer was filled with red ink in advance and 250 μA cm^{-2} current was applied for iontophoresis.

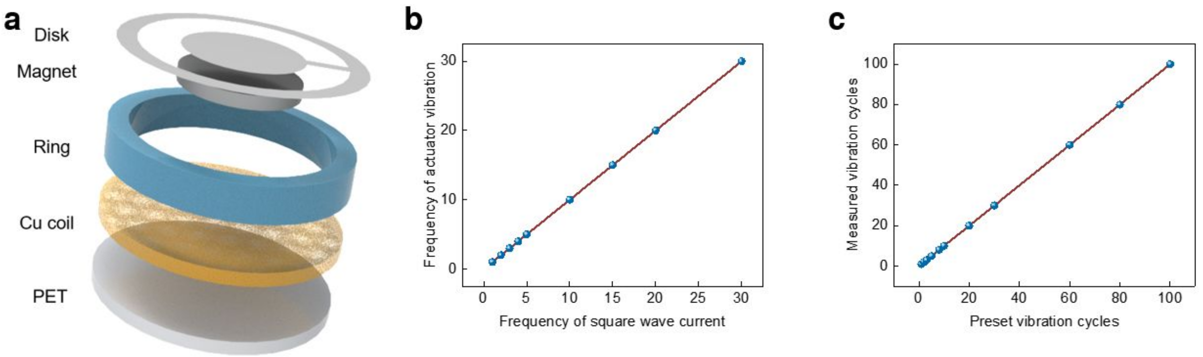

Figure S33. Characterization of the electromechanical actuator. **a** Assembly illustration of the actuator. **b** The correlation between the output vibration frequency of the actuator and the frequency of square wave current. **c** Correlation between the measured vibration cycles of the actuator and the preset vibration cycles.

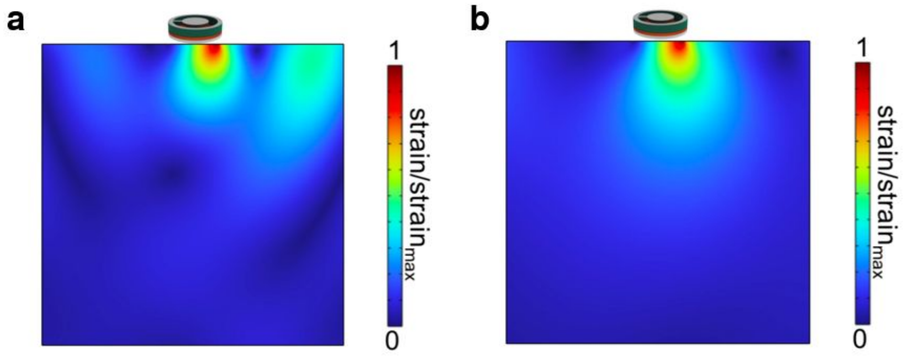

Figure S34. Numerical simulation of the on-body actuating effect. **(a** and **b)** Normalized finite element analysis results for distributions of strain throughout the tissue structure when the actuator is placed on the neck (with a module of about 10 kPa) (**a**) and arm (with a module of about 100 kPa) (**b**).

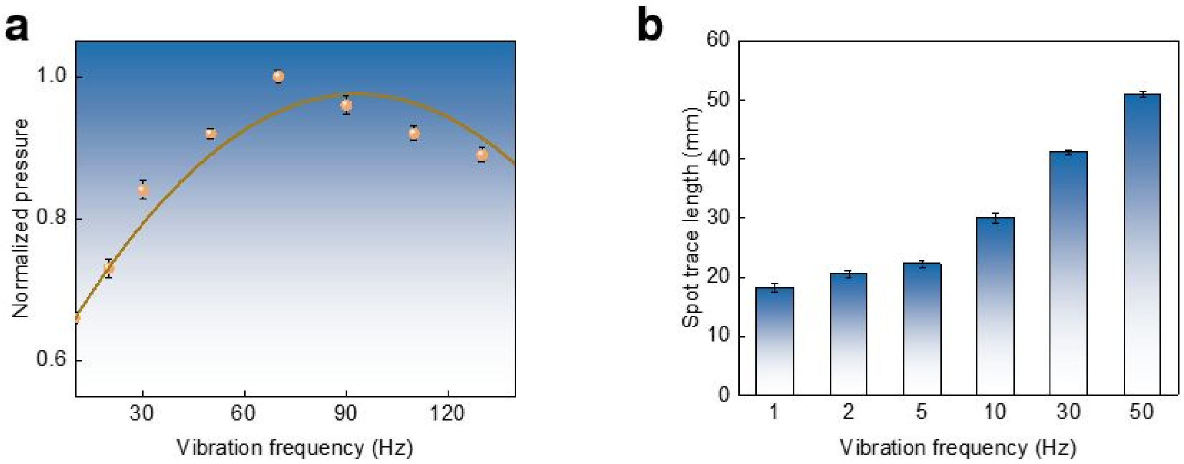

Figure S35. Influence of vibration frequency on the actuator output. **a** The correlation between the generated normalized pressure and the vibration frequency of the actuator. **b** The reflected spot trace length under different vibration frequency conditions.

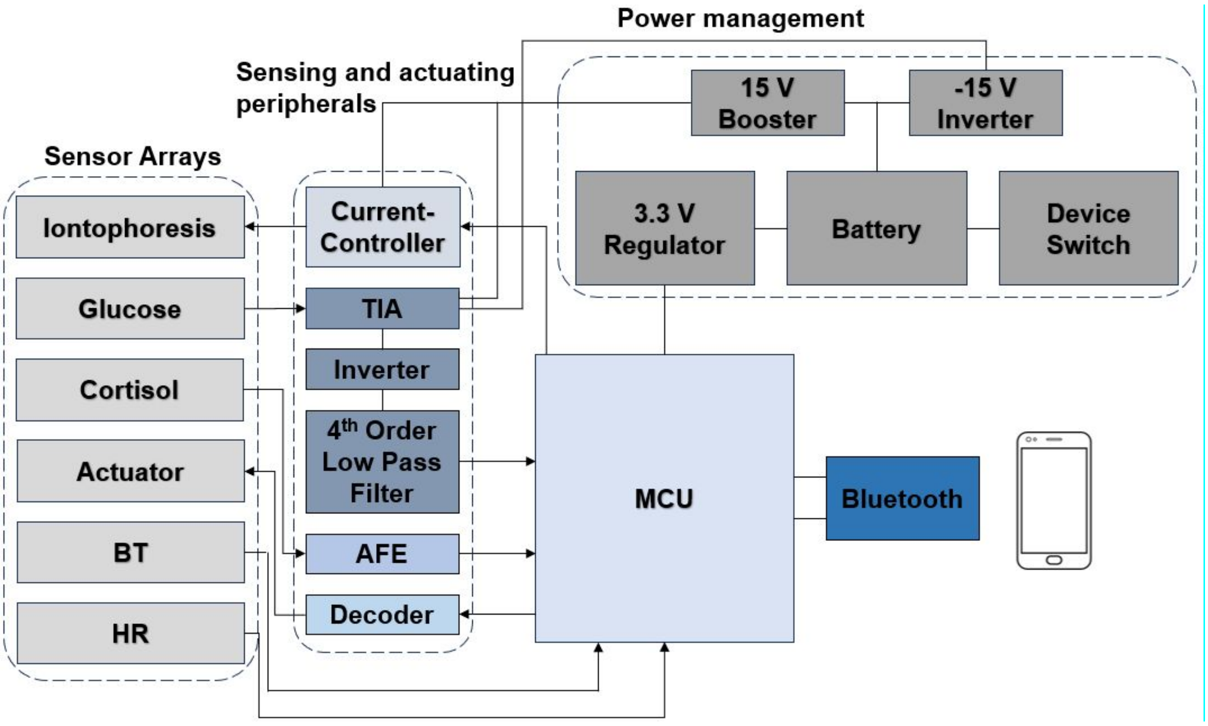

Figure S36. Block diagram of the FIRES for data collection and transmission.

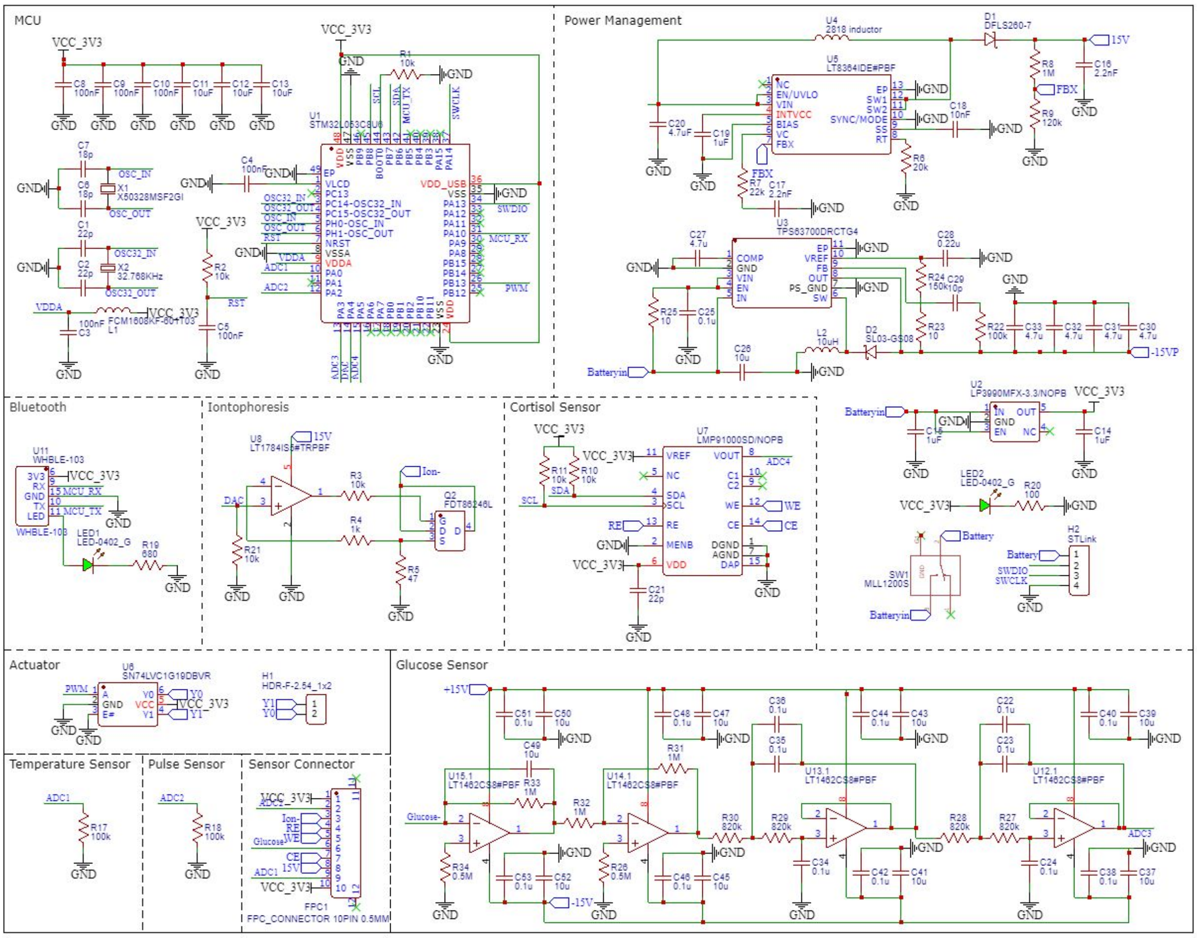

Figure S37. Detailed circuit schematics of the wearable system.

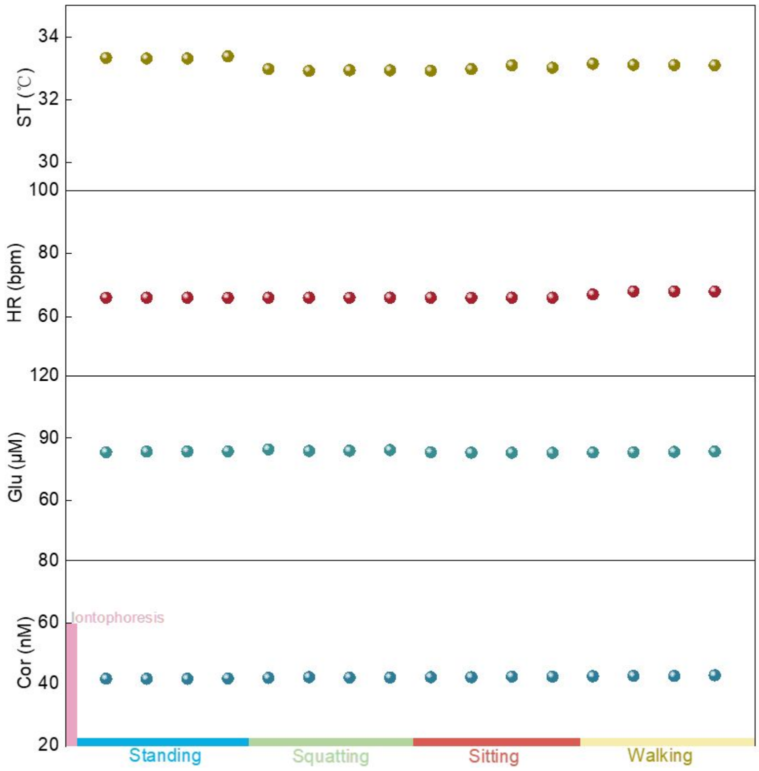

Figure S38. Biomarker monitoring during different states. The signal collection interval is 2 min.

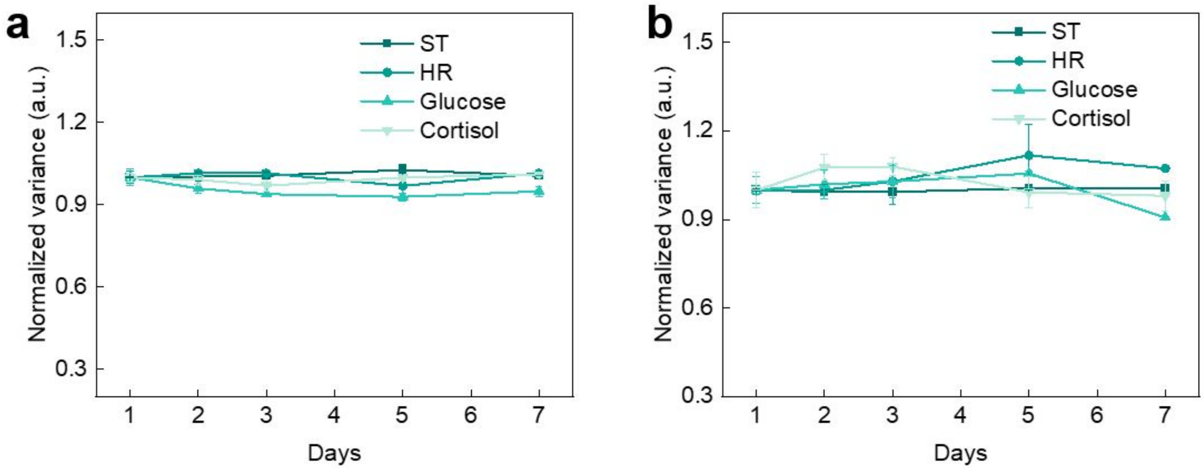

Figure S39. The variances of four biomarkers collected by the other two subjects within one week.

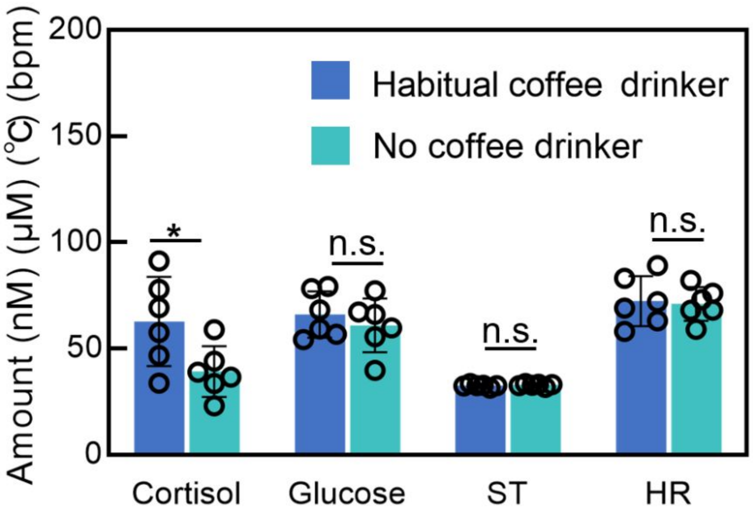

Figure S40. The biomarker levels within people with and without coffee diet.

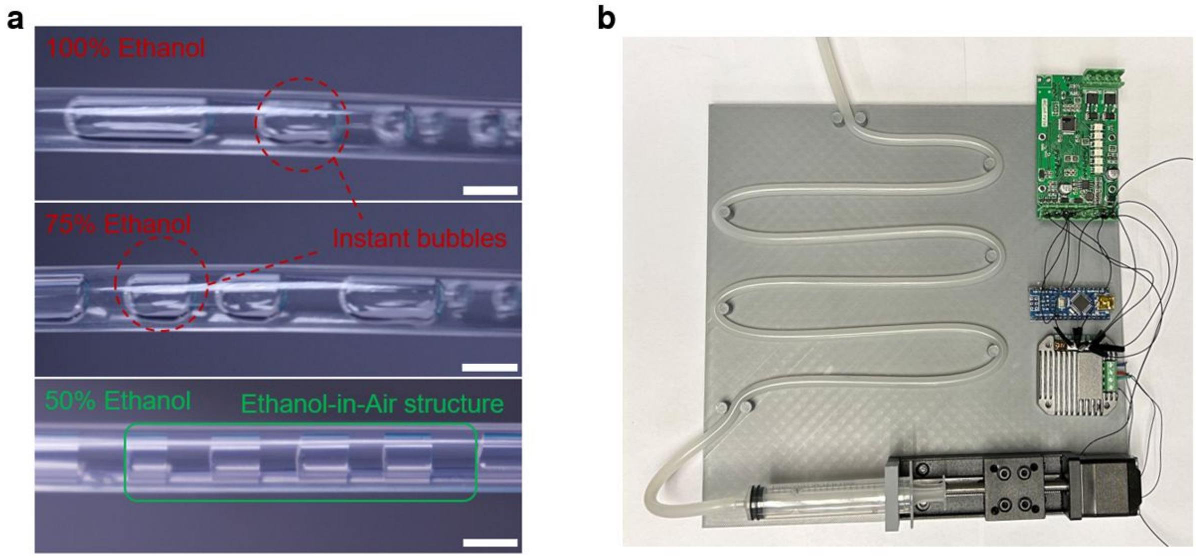

Figure S41. Chemical elution for cortisol regeneration. **a** The mixed structures using different concentrations of ethanol. **b** The optical images of the auto-refresh system.

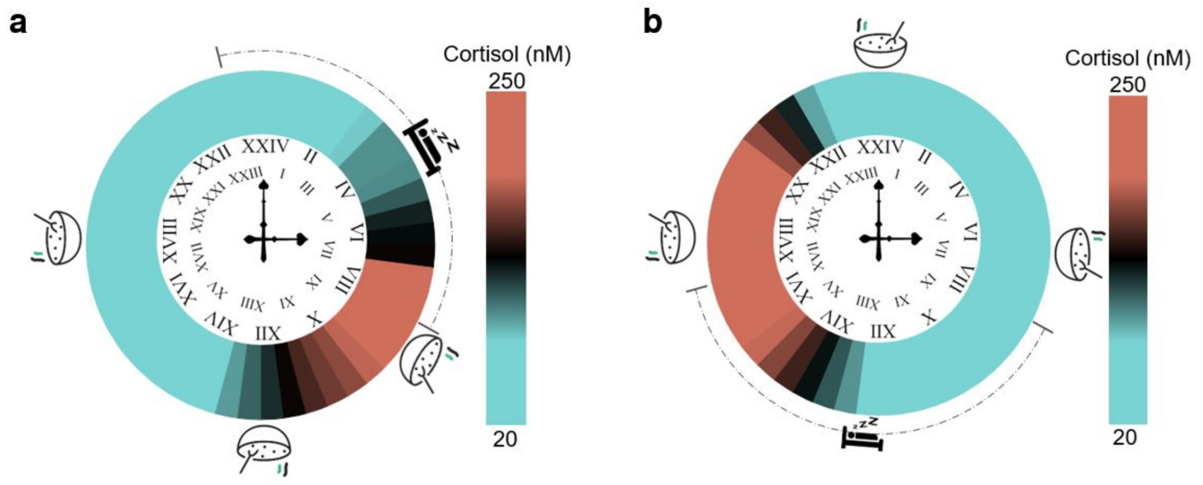

Figure S42. Influence of daily schedule on cortisol levels. **(a** and **b)** The 24 h cortisol levels in another two subjects, one with a regular daily schedule (**a**) and the other with an inverted daily schedule (**b**).

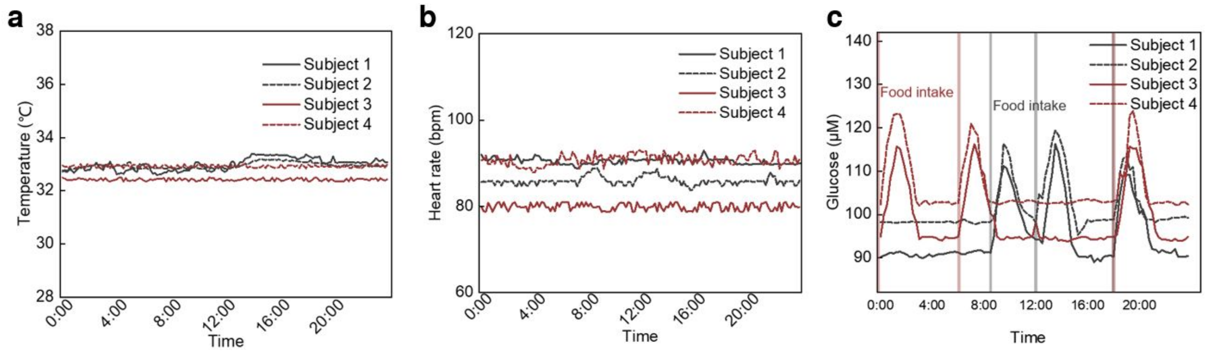

Figure S43. Influence of daily schedule on the levels of ST, HR and sweat glucose. (a-c) 24-h variances of ST (**a**), HR (**b**), and sweat glucose (**c**) levels of the subjects with regular daily schedule (black lines) and inverted daily schedule (red lines).

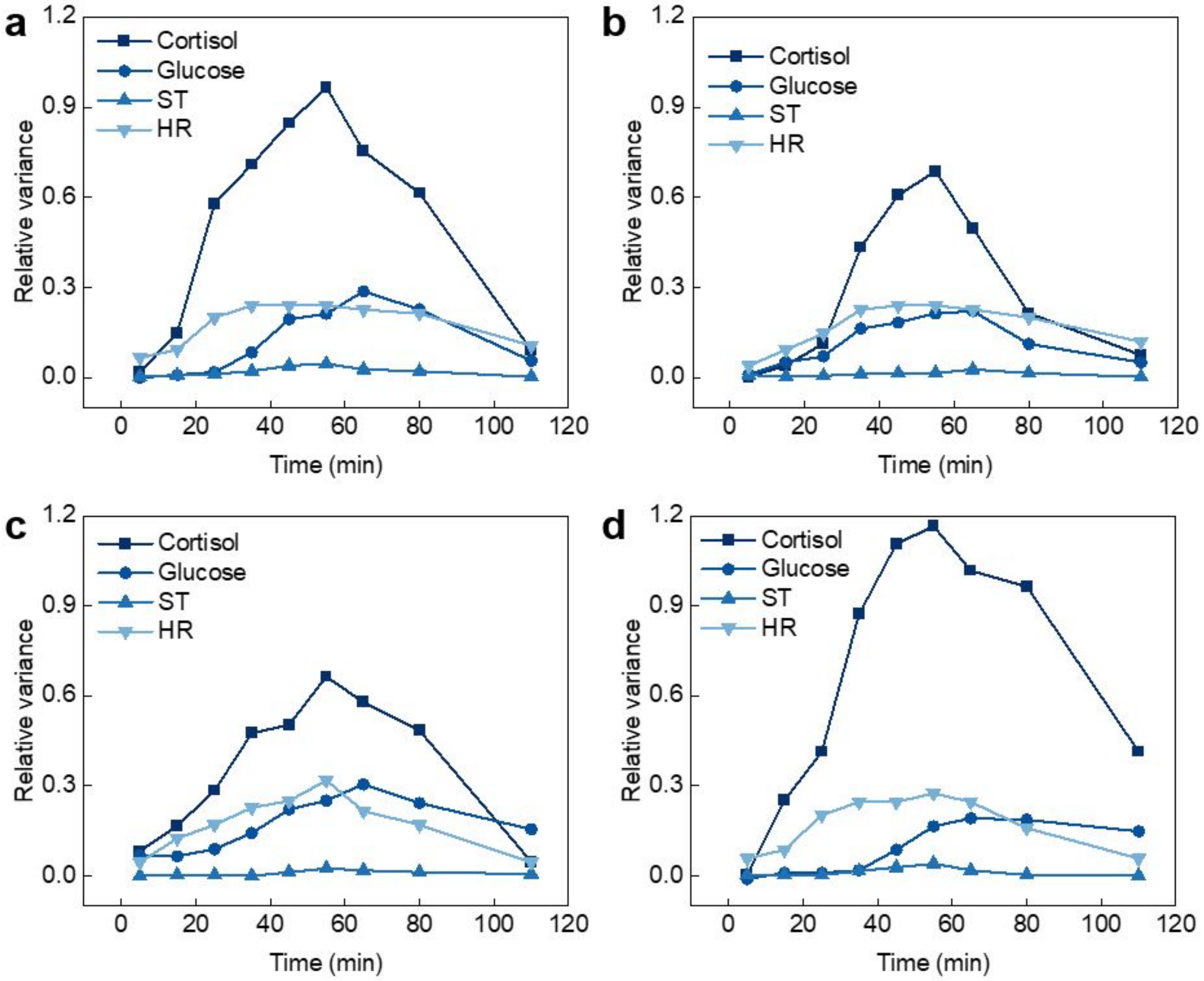

Figure S44. Dynamics of the biomarker panel during the TSST. **(a** and **b)** The dynamics of four biomarkers of a subject during the modified TSST for the first (**a**) and second (**b**) time. **(c** and **d)** the dynamics of four biomarkers of the other two subjects during the modified TSST period.

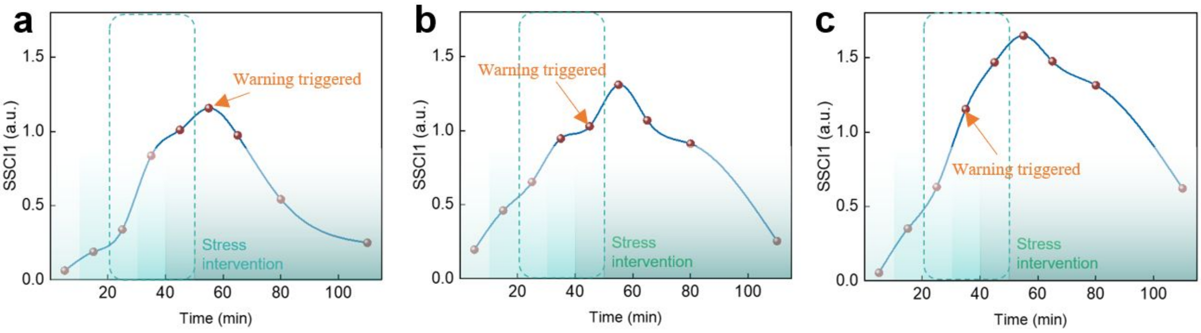

Figure S45. SSCI1 indexes during the TSST. **a** The SSCI1 dynamics of the subject in the modified TSST for the second time. **(b** and **c)** The SSCI1 dynamics of another two subjects in the modified TSST for the first time.

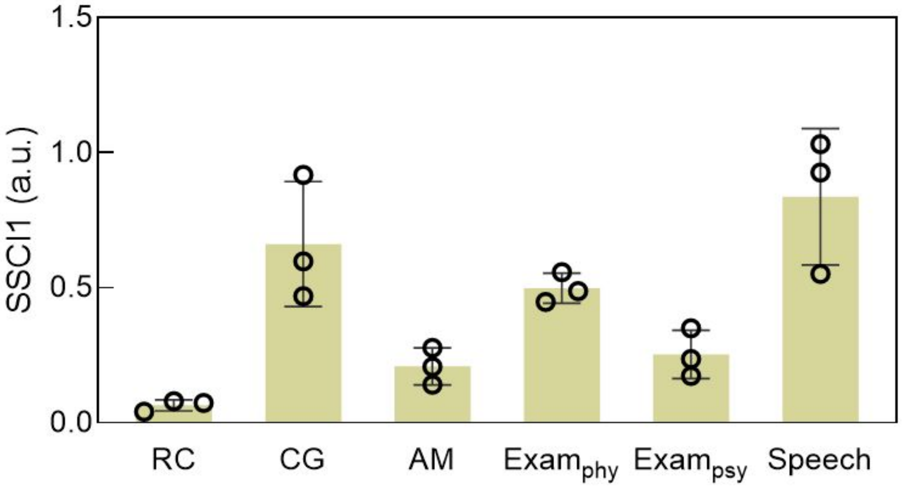

Figure S46. The influences of different actions on the SSCI1 value. RC: rest control; CG: competitive game; AM: action movie; Exam$_{phy}$: physical examination; Exam$_{psy}$: psychological examination.

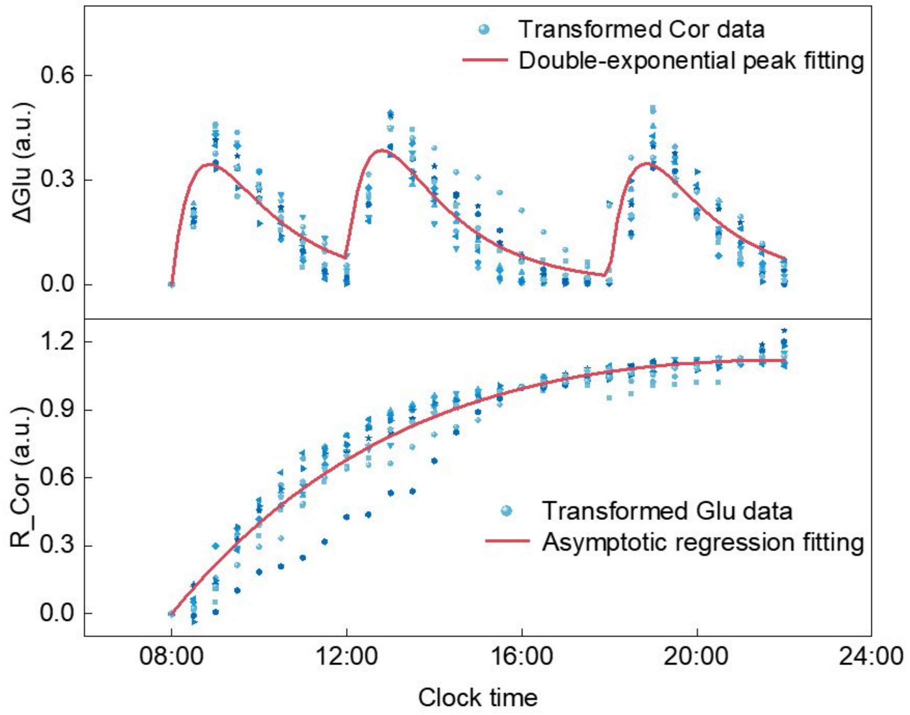

Figure S47. Function fitting of daytime test data for glucose and cortisol.

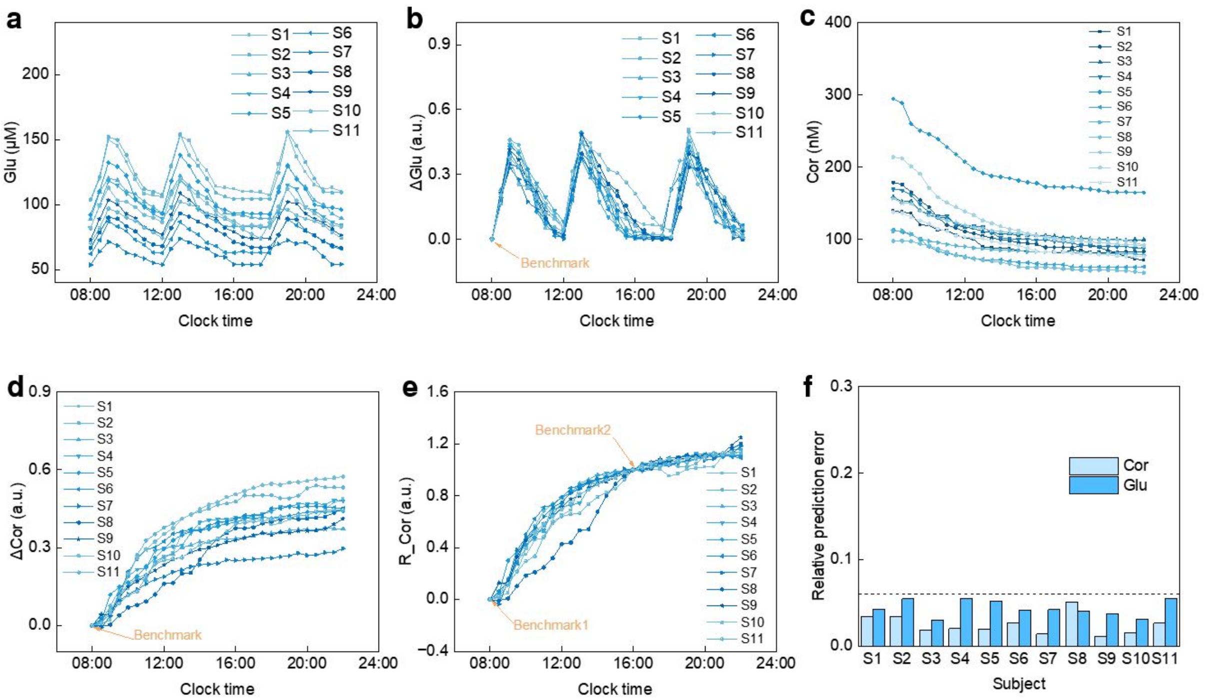

Figure S48. Data processing of cortisol and glucose for minimizing the influence of intra-individual variances. **a** The pristine glucose value of 11 subjects detected from 8:00 to 22:00. **b** Transformed glucose using one benchmark. **c** The pristine glucose value of 11 subjects detected from 8:00 to 22:00. **(d** and **e)** Transformed cortisol data using one (**d**) and two (**e**) benchmarks. **f** The average relative fitting error of cortisol and glucose.

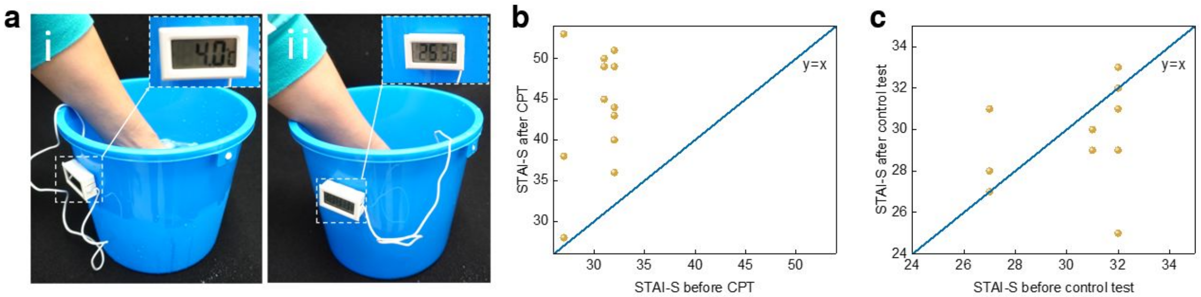

Figure S49. CPT for AS stimulation. **a** Schematic illustration of the CPT stimulation and control test. **(b** and **c)** The STAI- score before and after CPT (**b**) and control test (c). All data points are plotted above the diagonal for the CPT results, indicating increased STAI-S scores after the CPT. Comparatively, the data points are plotted near the diagonal for the control test results, representing no significant post-experimental elevation for the control test.

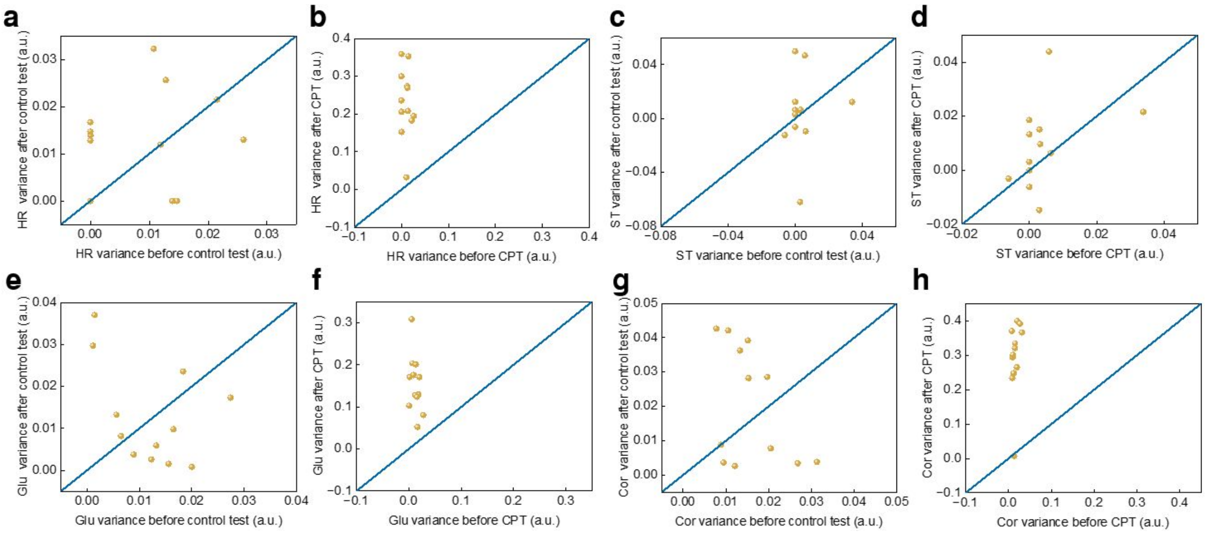

Figure S50. Influences of CPT and control test on the biomarker levels. **(a, c, e, g)** The variances of HR (**a**), ST (**c**), glucose (**e**) and cortisol (**g**) before and after the control test, respectively. **(b, d, f, h)** The variances of HR (**b**), body temperature (**d**), glucose (**f**) and cortisol (**h**) before and after the CPT test, respectively. The biomarker variance was calculated using the measured value and a control value collected 10 min before any tests.

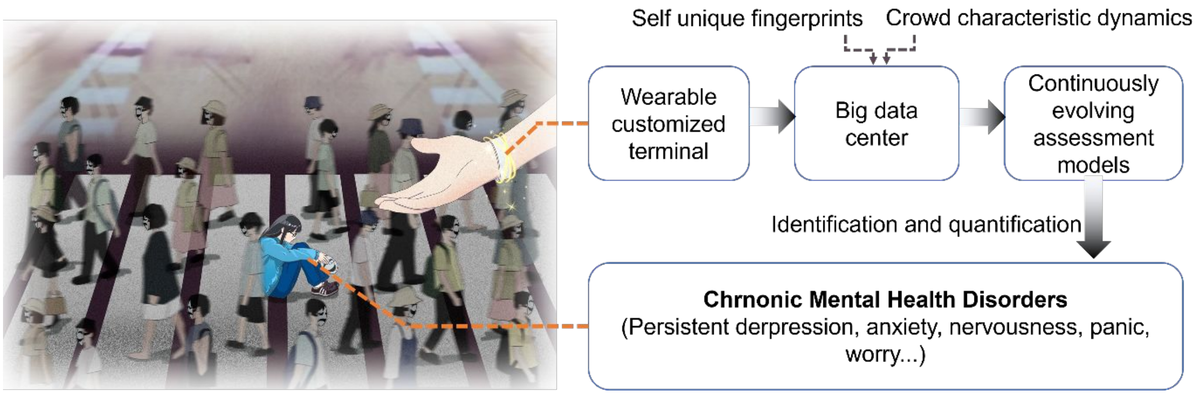

Figure S51. The concept of home care of chronic mental health disorders.

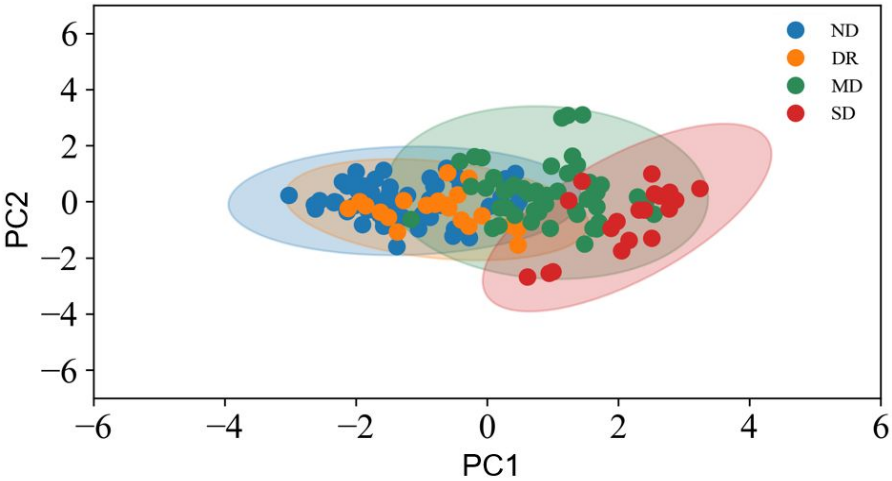

Figure S52. PCA results of the collected data. ND: non depression people; DR; people with depression risk; MD: mild depression patients; SD: severe depression patients.

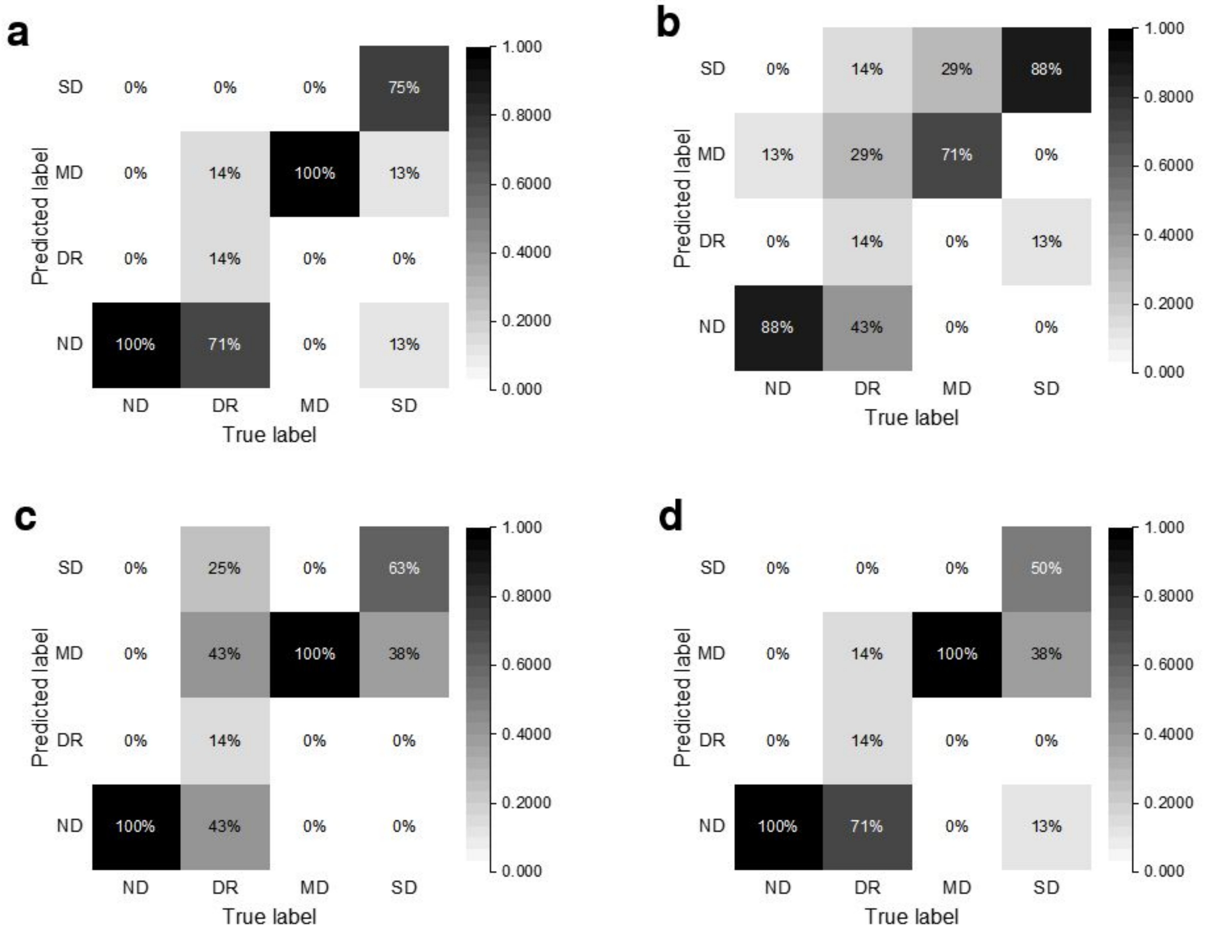

Figure S53. Performance of models for patient identification. **(a-d)** Confusion matrix for depression determination of SVM (**a**), Bayes (**b**), XGBoost (**c**), ANN (**d**) models, with the prediction accuracy of 73.3%, 66.7%, 66.7%, 66.7%, respectively.

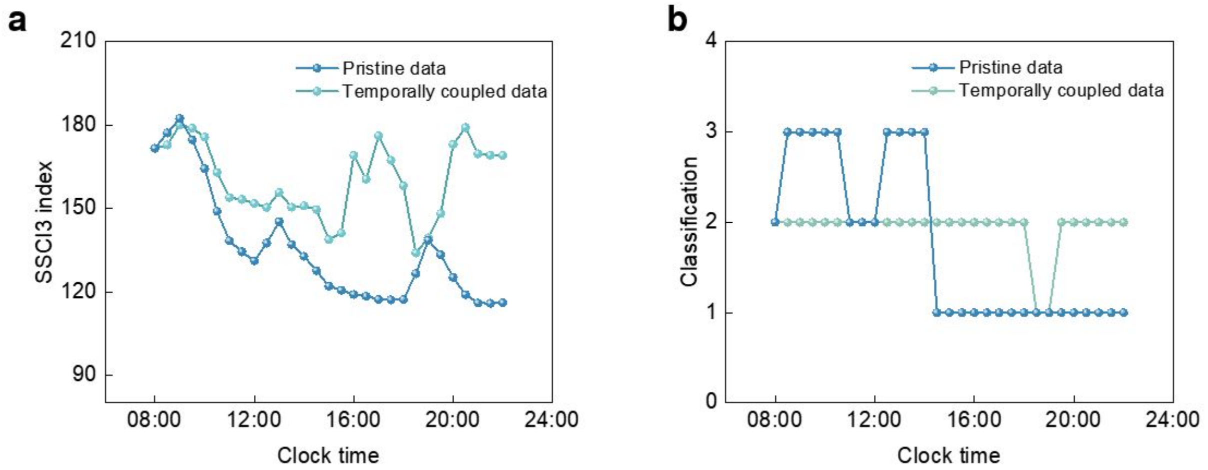

Figure S54. Continuous SSCI index and classification analysis over the daytime with and without data temporal processing.

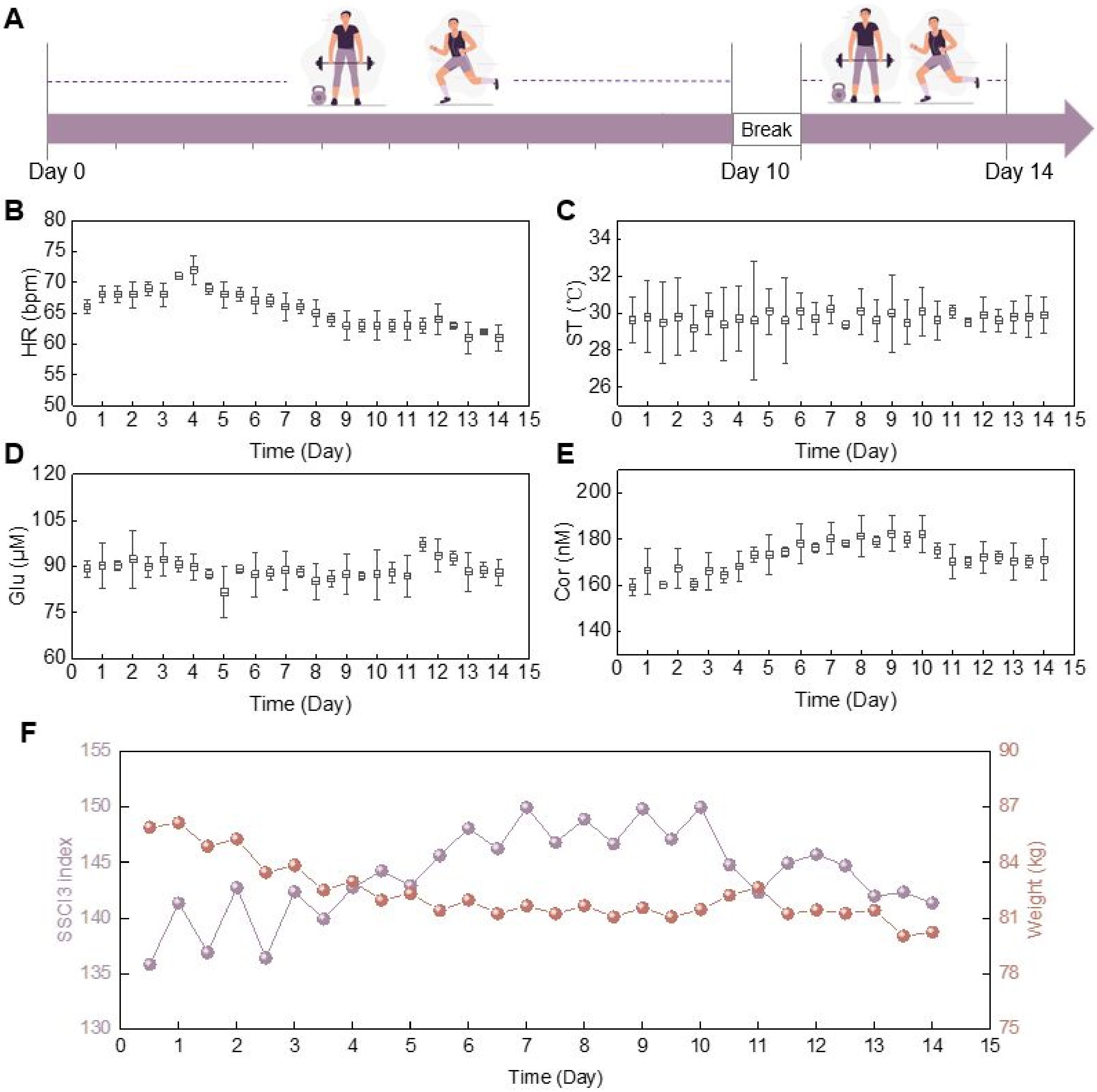

Figure S55. Stress level evaluation during the fat-loss intervention phase. a Schematic illustration of the fat-loss intervention process, which lasts for two weeks. One hour of strength training and half an hour of aerobic training are conducted daily except on day 10. The sport activity illustration images were designed by tartila/Freepik. **(b-e)** Continuous monitoring of (**b**) HR, (**c**) ST, (**d**) glucose and (**e**) cortisol during the process. Data were collected at 8:00 and 20:00 every day. The data were collected at 8:00 and 20:00 everyday. The glucose and cortisol data were converted to corresponding value at 8:00. **f** The evaluated SSCI3 index and weight dynamics during the fat-loss intervention process.

Table S1: Main biomarkers and their response characteristics with stress

Biomarkers	Circulation	Changes	Response mechanism correlated with stress	Reference
Cortisol	Blood, saliva, sweat, urine, hair and interstitial fluid	Increased	End product of the HPA axis; Regulate stress responses through interactions with MRs and GRs.	6, 15, 60
CRH	Blood	Increased	Modulated by the limbic system; Promoted secretion from the hypothalamus during stress; Stimulates the pituitary gland	6, 15
AVP	Blood	Increased	Promoted secretion from the hypothalamus during stress; As a neurotransmitter or neuromodulator involved in the regulation of cognition, emotion, etc	6, 15
ACTH	Blood	Increased	Promoted secretion from the pituitary during stress; Stimulates the adrenal cortex to release cortisol	6, 15
Adrenaline	Blood	Increased	Promoted secretion from the adrenal medulla during stress; Main end-product of LSAM axis	6, 15
Norepinephrine	Blood	Increased	Promoted secretion from the adrenal medulla during stress; Main end-product of LSAM axis	6, 15
Alpha-amylase	Saliva	Increased	Regulated by sympathetic nerve excitation; Changes in pancreatic islet function affect the secretion and activity of amylase	26, 61
S-IgA	Saliva, tears, nasal and bronchial secretions, gastrointestinal fluid, urine, and sweat	Increased (AS); decreased (CS)	Locally released when sympathetic nerve excited; Inhibited by excessive glucocorticoids	27
Dopamine	Blood, sweat	Increased	Dopaminergic neurons regulate stress resistance; End-product of LSAM axis	10, 28
β-endorphin	Blood, cerebrospinal fluid	Increased	Stimulated by CRH; Feedback regulated by glucocorticoids	62
Insulin	Blood	Increased	Inhibited by LSAM axis; Reduce glucose utilization during stress	63-65
Glucagon	Blood	Increased	Promoted by LSAM axis; Increase glycogenolysis during stress	63, 64
Glucose	Blood, sweat	Increased	Promoted by both HPA and LSAM; Gluconeogenesis is promoted; Glucose utilization is	

			reduced and glycogen breakdown is accelerated during stress	
Magnesium ion	Blood, sweat	Increased	Participate in maintaining neuronal health and vitality; Regulated by HPA axis	30, 66
Urine	NA	Increased	Promote the clearance of substances from the bladder; Strengthen urge to urinate	67
ST	NA	Increased	Promoted by activation of the DP/DTT→DMH pathway	16
BP	NA	Increased	Stimulated by high cardiac output and vessel constriction during stress	9
HR	NA	Increased	Regulated by sympathetic nerve excitation;	68-70
HRV	NA	Decreased in many cases	Affected by decreased parasympathetic nerve activity	32
Electrodermal activity	NA	Increased	Changes in blood vessels affect the activation of sweat glands, promoting sweat secretion and increasing skin electrical activity	69

Table S2: Review of portable stress detection devices

Device	Biomarker	Detection Method	Application Form	Reference
NOWATCH	HR, ST, SpO2, EDA		Watch	NOWATCH
Embrace Plus	EDA, ACC, ST, PR	PPG, accelerometer etc.	Watch	Empatica
E4	HR, ST, SpO2, EDA, PR	PPG, accelerometer etc.	Watch	Empatica
Sense 2	HR, HRV, ST, SpO2, RR		Watch	Fitbit
Ring 3	HR, ST, SpO2, EDA		Finger ring	Oura
Watch serial 9	HR, SpO2,		Watch	Apple
Moodmetric	EDA		Finger ring	Moodmetric
DTI-2	EDA, ST		Watch	Philips
Neocomimi	EEG		Headwear	Neurowear
Polar H7	HRV	ECG	chest strap	[71]
Sweat Sensing Patch	Cor, Mg^{2+}, pH	Electrochemical (MIP etc.)	E-patch	[29]
GO-based biosensor	Cor	Electrochemical (antibody)	POC in vitro sensor	[72]
Skin-conformal bioelectronic	EDA, ST	Digital potentiometer, digital thermistor	E-patch	[49]
Integrated commercial sensor array	ST, Breath, ECG, EDA		Cloth	[73]
Enzymatic modified screen printed electrode	Alpha-amylase	Electrochemical (Enzyme)	Disposable electrode	[26]
Self assembling immune biosensors	Cor	Electrochemical (Antibody)	POC in vitro sensor	[74]
Colorimetry Sensor Platform	Alpha-amylase	Colorimetry	Hand-held device	[61]
CortiWatch	Cor	Electrochemical (antibody)	Watch	[75]
Antibody functionalized MoS2 nanosheets-based biosensor	Cor	Electrochemical (antibody)	Watch	[76]
Three-dimensional Au nanostructure-based biosensor	Cor	Electrochemical (antibody)	lab-on-a-patch system	[77]
Laser induced graphene based biosensor	Cor	Electrochemical (antibody)	E-patch	[34]
Molecularly selective nanoporous membrane-based biosensor	Cor	Organic electrochemical transistors	E-patch	[78]

Aptamer-field-effect transistor	Cor	Field-effect transistor	Watch	36
RABio w8-based system	EEG, ECG, EMG, EDA	Electrodes and signal collectors	Wired connection device	79
Graphene field-effect transistor-based immunosensor	Cor	Field effect transistor	Contact lenses	80
Touch based MIP electrochemical sensor	Cor	Electrochemical (MIP)	Touch-based platform	81
A stretchable microfluidic immunobiosensor	NPY	Electrochemical (antibody)	Patch	82
SKINTRONICS	EDA, ST	Electrode pair	Patch	83
A resonance Circuit based platform	Cor, pH, ST	Electrochemical, PEG capacitor	Patch	84
A sandwiched electrode system	Cor	Electrochemical (aptamer)	Patch	85
A multisensor ring-shaped probe	HR, HRV, SpO2, EDA	PPG; electrode pair	Finger-cot	86
Ni–Co MOF nanosheet-decorated CNTs/PU film	Cor	Electrochemical (aptamer)	Patch	87
EmoSense	Microgestures	Wept frequency capacitive sensing technology	Wristband	88
Ag triangle nanoplates assembled on PVC/SEBS membrane	Cor	Surface-enhanced Raman scattering	Patch	89
Photonic hydrogel (PH)-based sensors	Cor	Electrochemical (MIP and antibody)	Patch	90
An Ag/rGO-modified carbon paste microneedle electrode	5-HT	Electrochemical	Microneedle patch	91
An extended-gate AlGaN/GaN high electron mobility transistor	Cor	Field-effect transistor	Patch	92
An MXene modified stranded carbon yarn	Dopamine	Electrochemical	Bandaid	93
A piezoelectric nanocomposite material-based platform	Pulse; ST	PZT; Resistance temperature detector	Patch	94
A laser-induced graphene electrode array	NPY	Photoelectrochemical sensor	Patch	95
An MOF-based microneedle	Cor	MOF	Microneedle patch	96

An LC resonator-based sensor	Cor	MIP-aided radio-frequency technique	Patch	97
A sprinted carbon electrode	Cor	Electrochemical (MIP)	Patch	98
CARES	Lactate, Glu, Uric acid, Na^+, K^+, NH_4^+, ST, EDA, pulse rate	Physicochemical	Electronic skin	48
An optical sensor	HR	PPG	Necklace	99
An integrated smartwatch	Cor, HR	Electrochemical (MIP); PPG	Watch	100
One channel system	EEG, EDA	Electrode pair	Headband	101

Cor: cortisol; Glu: glucose; NPY: neuropeptide-Y; 5-HT: 5-hydroxytryptamine; HR: heart rate; HRV: heart rate variability; PR: pulse rate; ST: skin temperature; EDA: electrodermal activity; SpO_2: Oxygen saturation; ACC: acceleration; ECG: electrocardiogram; EEG: electroencephalogram; PPG: Photoplethysmography; PEG: polyethylene glycol

Table S3: Spike-and-recovery assays of cortisol and glucose

	Spiked	Determined	Recovery (%)	RSD (%)
Glucose	50 μM	50.97±3.12 μM	102%	6.12
	150 μM	156.38±5.68 μM	104%	3.63
	200 μM	203.18±6.25 μM	102%	3.08
Cortisol	100 nM	99.12±9.23 nM	99.1%	9.31
	300 nM	311.33±12.49 nM	104%	4.01
	500 nM	516.23±19.26 nM	103.2%	3.73

Table S4: Selected function for glucose data fitting

Function Name	Formula	Description	Final Loss
Sine	$f(t) = F_0 + Asin(\omega t + b)$	A trigonometric function	2.20
PsdVoigt	$f(t) = F_0 + A[\mu e^{-\frac{(t-t_0)^2}{-2\sigma^2}} + (1 - \mu)\frac{(\tau/2)^2}{(t-t_0)^2 + (\tau/2)^2}]$	An approximation for the Voigt function, which is a convolution of Gaussian and Lorentzian function, commonly used to fit signals with complex inflection points.	2.57
Poly	$f(t) = a_0 + a_1 t + \dots + a_{n-1} t^{n-1}$	A smooth function composed of the superposition of power functions	3.15
InvsPoly	$f(t) = F_0 + \frac{A}{1 + A_1(2\frac{t-t_c}{\omega})^2 + A_2(2\frac{t-t_c}{\omega})^4 + A_3(2\frac{t-t_c}{\omega})^6}$	An inverse polynomial peak function with center, involving complicated combinations of roots of various degrees.	3.12
Gauss	$f(t) = \sum_{i=1}^{3} h_i \left(-\frac{(t-\mu_i)^2}{2\sigma_i{}^2}\right) H(t-t_i)$	A bell shaped function with characteristic symmetry	2.05
DEP	$f(t) = \sum_{i=1}^{3} A_i (e^{k_{1i}(t-t_i)} - e^{k_{2i}(t-t_i)}) H(t-t_i)$	The superposition of two exponential functions, the difference in exponential parameters can characterize different rates of increase or decrease	1.57

Table S5: Selected function for cortisol data fitting

Function Name	Formula	Description	Final Loss
Power function	$f(t) = [\frac{(t-8)}{8}]^b$	A function that rises rapidly and then slowly within the domain of t>0	2.66
Logarithmic function	$f(t) = a\log(t-7) + b(t-8)$	A rising logarithmic function with a linear offset superimposed simultaneously	1.88
Asymptotic regression	$f(t) = A(1-e^{-B(t-8)}) + C(t-8)$	A function with a gradually decreasing growth rate, superimposed with a linear offset	1.43
Rational	$f(t) = \frac{b+ct}{1+at}$	Rational function with 1st order of numerator and 1st order of denominator	2.04
ExpAssoc	$f(t) = a_1(1-e^{-k_1 t}) + a_2 e^{-k_2 t}$+c	Two-phase exponential association function	3.08
ExpDecay	$f(t) = a_1 e^{-\frac{t-t_0}{b}}$	One-phase exponential decay function with time offset (fixed t_0)	2.63

Table S6: Comprehensive comparison with related stress management works

Cortisol included?	Flexible and wireless?	Sensor robustness	Reusable?	Clinical validation	Reference
No	No	\	Yes	No	[99]
No	Yes	Average	Yes	No	[83]
Yes	Not exactly	Average	No	No	[98]
Yes	Not exactly	Average	No	No	[100]
Yes	Not exactly	Average	No	No	[96]
No	Yes	Average	Yes	No	[48]
Yes	Yes	High	Yes	Yes	This work

Reference

1. Selye, H., A Syndrome Produced by Diverse Nocuous Agents. *Nature* **1936,** *138*, 32.
2. Lazarus, R. S.;Folkman, S., *Stress, Appraisal, and Coping*. Springer: New York, 1984.
3. Taylor, S. E.; Klein, L. C.; Lewis, B. P.; Gruenewald, T. L.; Gurung, R. A. R.;Updegraff, J. A., Biobehavioral Responses to Stress in Females Tend-and-Befriend, Not Fight-or-Flight. *Psychol. Rev.* **2000,** *107*, 411–429.
4. Hobfoll, S. E., Conservation of Resources a New Attempt at Conceptualizing Stress. *American Psychologist* **1989,** *44*, 513-524.
5. Biltz, R. G.; Sawicki, C. M.; Sheridan, J. F.;Godbout, J. P., The Neuroimmunology of Social-Stress-Induced Sensitization. *Nat. Immunol.* **2022,** *23*, 1527-1535.
6. Russell, G.;Lightman, S., The Human Stress Response. *Nat. Rev. Endocrinol.* **2019,** *15*, 525-534.
7. *Fundamental Facts About Mental Hralth*. Mental Health Foundation: London, 2016.
8. Bienertova-Vasku, J.; Lenart, P.;Scheringer, M., Eustress and Distress: Neither Good nor Bad, but Rather the Same? *BioEssays* **2020,** *42*, 1900238.
9. Schaare, H. L.; Blochl, M.; Kumral, D.; Uhlig, M.; Lemcke, L.; Valk, S. L.;Villringer, A., Associations between Mental Health, Blood Pressure and the Development of Hypertension. *Nat. Commun.* **2023,** *14*, 1953.
10. Ma, Y.;Kroemer, G., The Cancer-Immune Dialogue in the Context of Stress. *Nat. Rev. Immunol.* **2023**, 264–281.
11. Hankin, B. L.; Mermelstein, R.;Roesch, L., Sex Differences in Adolescent Depression: Stress Exposure and Reactivity Models. *Child Development* **2007,** *78*, 279-295.
12. Cole, D. A.; Nolen-Hoeksema, S.; Girgus, J.;Paul, G., Stress Exposure and Stress Generation in Child and Adolescent Depression: A Latent Trait-State-Error Approach to Longitudinal Analyses. *Journal of Abnormal Psychology* **2006,** *115*, 40-51.
13. Graber, J. A.;Sontag, L. M., Internalizing Problems During Adolescence. In *Handbook of Adolescent Psychology*, Lerner, I. R. M.; Steinberg, L., Eds. Wiley: New Jersey, 2009.
14. Laryea, G.; Muglia, L.; Arnett, M.;Muglia, L. J., Dissection of Glucocorticoid Receptor-Mediated Inhibition of the Hypothalamic-Pituitary-Adrenal Axis by Gene Targeting in Mice. *Front. Neuroendocrinol.* **2015,** *36*, 150-164.
15. de Kloet, E. R.; Joels, M.;Holsboer, F., Stress and the Brain: From Adaptation to Disease. *Nat. Rev. Neurosci.* **2005,** *6*, 463-475.
16. Kataoka, N.; Shima, Y.; Nakajima, K.;Nakamura, K., A Central Master Driver of Psychosocial Stress Responses in the Rat. *Science* **2020,** *367*, 1105-1112.
17. Holsboer, F.; Lauer, C. J.; Schreiber, W.;Krieg, J.-C., Altered Hypothalamic-Pituitary-Adrenocortical Regulation in Healthy Subjects at High Familial Risk for Affective Disorders. *Neuroendocrinology* **1995,** *62*, 340-347.
18. Arias, D.; Saxena, S.;Verguet, S., Quantifying the Global Burden of Mental Disorders and Their Economic Value. *eClinicalMedicine* **2022,** *54*, 101675.
19. Global, Regional, and National Burden of 12 Mental Disorders in 204 Countries and Territories, 1990–2019: A Systematic Analysis for the Global Burden of Disease Study 2019. *Lancet Psychiat.* **2022,** *9*, 137-150.
20. Weng, H.; Deng, L.; Wang, T.; Xu, H.; Wu, J.; Zhou, Q.; Yu, L.; Chen, B.; Huang, L. a.; Qu, Y.; Zhou, L.;Chen, X., Humid Heat Environment Causes Anxiety-Like Disorder Via Impairing Gut Microbiota and Bile Acid Metabolism in Mice. *Nat. Commun.* **2024,** *15*, 5697.
21. Agorastos, A.;Chrousos, G. P., The Neuroendocrinology of Stress: The Stress-Related Continuum of Chronic Disease Development. *Mol. Psychiatry* **2021,** *27*, 502-513.
22. McEwen, B. S.; Nasca, C.;Gray, J. D., Stress Effects on Neuronal Structure: Hippocampus, Amygdala, and Prefrontal Cortex. *Neuropsychopharmacology* **2015,** *41*, 3-23.

23. Won, E.;Kim, Y.-K., Stress, the Autonomic Nervous System, and the Immune-Kynurenine Pathway in the Etiology of Depression. *Current Neuropharmacology* **2016,** *14*, 665-673.
24. Gibbison, B.; Spiga, F.; Walker, J. J.; Russell, G. M.; Stevenson, K.; Kershaw, Y.; Zhao, Z.; Henley, D.; Angelini, G. D.;Lightman, S. L., Dynamic Pituitary-Adrenal Interactions in Response to Cardiac Surgery*. *Critical Care Medicine* **2015,** *43*, 791-800.
25. Kuo, T.; McQueen, A.; Chen, T.-C.;Wang, J.-C., Regulation of Glucose Homeostasis by Glucocorticoids. In *Glucocorticoid Signaling. Advances in Experimental Medicine and Biology*, JC, W.; C, H., Eds. Springer, New York, NY: 2015; Vol. 872, pp 99-126.
26. Mahosenaho, M.; Caprio, F.; Micheli, L.; Sesay, A. M.; Palleschi, G.;Virtanen, V., A Disposable Biosensor for the Determination of Alpha-Amylase in Human Saliva. *Microchim. Acta* **2010,** *170*, 243-249.
27. Viena, T. D.; Banks, J. B.; Barbu, I. M.; Schulman, A. H.;Tartar, J. L., Differential Effects of Mild Chronic Stress on Cortisol and S-Iga Responses to an Acute Stressor. *Biol. Psychol.* **2012,** *91*, 307-311.
28. Willmore, L.; Cameron, C.; Yang, J.; Witten, I. B.;Falkner, A. L., Behavioural and Dopaminergic Signatures of Resilience. *Nature* **2022,** *611*, 124-132.
29. Zhao, H.; Zhang, X.; Qin, Y.; Xia, Y.; Xu, X.; Sun, X.; Yu, D.; Mugo, S. M.; Wang, D.;Zhang, Q., An Integrated Wearable Sweat Sensing Patch for Passive Continuous Analysis of Stress Biomarkers at Rest. *Adv. Func. Mater.* **2022,** *33*, 2212083.
30. Botturi, A.; Ciappolino, V.; Delvecchio, G.; Boscutti, A.; Viscardi, B.;Brambilla, P., The Role and the Effect of Magnesium in Mental Disorders: A Systematic Review. *Nutrients* **2020,** *12*, 1661.
31. Kivimäki, M.; Bartolomucci, A.;Kawachi, I., The Multiple Roles of Life Stress in Metabolic Disorders. *Nat. Rev. Endocrinol.* **2022,** *19*, 10-27.
32. Kim, H. G.; Cheon, E. J.; Bai, D. S.; Lee, Y. H.;Koo, B. H., Stress and Heart Rate Variability: A Meta-Analysis and Review of the Literature. *Psychiatry Invest.* **2018,** *15*, 235-245.
33. Parlak, O., Portable and Wearable Real-Time Stress Monitoring: A Critical Review. *Sensor Actuator. Rep.* **2021,** *3*, 100036.
34. Torrente-Rodríguez, R. M.; Tu, J.; Yang, Y.; Min, J.; Wang, M.; Song, Y.; Yu, Y.; Xu, C.; Ye, C.; IsHak, W. W.;Gao, W., Investigation of Cortisol Dynamics in Human Sweat Using a Graphene-Based Wireless Mhealth System. *Matter* **2020,** *2*, 921-937.
35. Xu, Y.;Suleiman, A. A., Reusable Amperometric Immunosensor for the Determination of Cortisol. *Analytical Letters* **1997,** *30*, 2675-2689.
36. Wang, B.; Zhao, C.; Wang, Z.; Yang, K.-A.; Cheng, X.; Liu, W.; Yu, W.; Lin, S.; Zhao, Y.; Cheung, K. M.; Lin, H.; Hojaiji, H.; Weiss, P. S.; Stojanović, M. N.; Tomiyama, A. J.; Andrews, A. M.;Emaminejad, S., Wearable Aptamer Field-Effect Transistor Sensing System for Noninvasive Cortisol Monitoring. *Sci. Adv.* **2022,** *8*, eabk0967.
37. Shirin, H.; Pomeranz, M.; Liberman, E.; Kedem, P.; Bartoov, M.; Arber, N.; Baruch, Y.; Arber, L.; Hirschfeld, Z.; Pinkhas, J.; Aronson, M.;Berliner, A. S., Differentiation between Major and Milder Acute Mental Stress by Means of the Leukocyte Adhesiveness/Aggregation Test. *Behav. Med.* **2010,** *19*, 175-180.
38. Chandola, T.; Britton, A.; Brunner, E.; Hemingway, H.; Malik, M.; Kumari, M.; Badrick, E.; Kivimaki, M.;Marmot, M., Work Stress and Coronary Heart Disease: What Are the Mechanisms? *European Heart Journal* **2008,** *29*, 640-648.
39. Vrijkotte, T. G. M.; van Doornen, L. J. P.;de Geus, E. J. C., Effects of Work Stress on Ambulatory Blood Pressure, Heart Rate, and Heart Rate Variability. *Hypertension* **2000,** *35*, 880-886.

40. Moyer, J.; Wilson, D.; Finkelshtein, I.; Wong, B.;Potts, R., Correlation between Sweat Glucose and Blood Glucose in Subjects with Diabetes. *Diabetes Technology & Therapeutics* **2012,** *14*, 398-402.
41. La Count, T. D.; Jajack, A.; Heikenfeld, J.;Kasting, G. B., Modeling Glucose Transport from Systemic Circulation to Sweat. *Journal of Pharmaceutical Sciences* **2019,** *108*, 364-371.
42. Nyein, H. Y. Y.; Bariya, M.; Kivimäki, L.; Uusitalo, S.; Liaw, T. S.; Jansson, E.; Ahn, C. H.; Hangasky, J. A.; Zhao, J.; Lin, Y.; Happonen, T.; Chao, M.; Liedert, C.; Zhao, Y.; Tai, L.-C.; Hiltunen, J.;Javey, A., Regional and Correlative Sweat Analysis Using High-Throughput Microfluidic Sensing Patches toward Decoding Sweat. *Science Advances 5*, eaaw9906.
43. Heikenfeld, J.; Jajack, A.; Feldman, B.; Granger, S. W.; Gaitonde, S.; Begtrup, G.;Katchman, B. A., Accessing Analytes in Biofluids for Peripheral Biochemical Monitoring. *Nature Biotechnology* **2019,** *37*, 407-419.
44. Min, J.; Tu, J.; Xu, C.; Lukas, H.; Shin, S.; Yang, Y.; Solomon, S. A.; Mukasa, D.;Gao, W., Skin-Interfaced Wearable Sweat Sensors for Precision Medicine. *Chemical Reviews* **2023,** *123*, 5049-5138.
45. Kim, S.-R.; Zhan, Y.; Davis, N.; Bellamkonda, S.; Gillan, L.; Hakola, E.; Hiltunen, J.;Javey, A., Electrodermal Activity as a Proxy for Sweat Rate Monitoring During Physical and Mental Activities. *Nature Electronics* **2025,** *8*, 353-361.
46. Niven, D. J.; Gaudet, J. E.; Laupland, K. B.; Mrklas, K. J.; Roberts, D. J.;Stelfox, H. T., Accuracy of Peripheral Thermometers for Estimating Temperature. *Annals of Internal Medicine* **2015,** *163*, 768-777.
47. Geneva, I. I.; Cuzzo, B.; Fazili, T.;Javaid, W., Normal Body Temperature: A Systematic Review. *Open Forum Infectious Diseases* **2019,** *6*, ofz032.
48. Xu, C.; Song, Y.; Sempionatto, J. R.; Solomon, S. A.; Yu, Y.; Nyein, H. Y. Y.; Tay, R. Y.; Li, J.; Heng, W.; Min, J.; Lao, A.; Hsiai, T. K.; Sumner, J. A.;Gao, W., A Physicochemical-Sensing Electronic Skin for Stress Response Monitoring. *Nat. Electron.* **2024,** *7*, 168-179.
49. Kim, H.; Kim, Y. S.; Mahmood, M.; Kwon, S.; Zavanelli, N.; Kim, H. S.; Rim, Y. S.; Epps, F.;Yeo, W. H., Fully Integrated, Stretchable, Wireless Skin-Conformal Bioelectronics for Continuous Stress Monitoring in Daily Life. *Adv. Sci.* **2020,** *7*, 2000810.
50. Jia, Y.; Chen, S.; Wang, Q.;Li, J., Recent Progress in Biosensor Regeneration Techniques. *Nanoscale* **2024,** *16*, 2834-2846.
51. Zhang, X.; Song, C.; Yang, K.; Hong, W.; Lu, Y.; Yu, P.;Mao, L., Photoinduced Regeneration of an Aptamer-Based Electrochemical Sensor for Sensitively Detecting Adenosine Triphosphate. *Anal. Chem.* **2018,** *90*, 4968-4971.
52. Chen, S.; Liu, T.-L.; Dong, Y.;Li, J., A Wireless, Regeneratable Cocaine Sensing Scheme Enabled by Allosteric Regulation of Ph Sensitive Aptamers. *ACS Nano* **2022,** *16*, 20922-20936.
53. Wang, M.; Yang, Y.; Min, J.; Song, Y.; Tu, J.; Mukasa, D.; Ye, C.; Xu, C.; Heflin, N.; McCune, J. S.; Hsiai, T. K.; Li, Z.;Gao, W., A Wearable Electrochemical Biosensor for the Monitoring of Metabolites and Nutrients. *Nat. Biomed. Eng.* **2022,** *6*, 1225-1235.
54. Zhu, D.; Liu, J.; Tang, Y.;Xing, D., A Reusable DNA Biosensor for the Detection of Genetically Modified Organism Using Magnetic Bead-Based Electrochemiluminescence. *Sens. Actuators B Chem.* **2010,** *149*, 221-225.
55. Wang, R.; Zhou, X.;Shi, H., Triple Functional DNA–Protein Conjugates: Signal Probes for Pb2+ Using Evanescent Wave-Induced Emission. *Biosens. Bioelectron.* **2015,** *74*, 78-84.
56. Wang, R.; Zhou, X.; Shi, H.;Luo, Y., T–T Mismatch-Driven Biosensor Using Triple Functional DNA-Protein Conjugates for Facile Detection of Hg2+. *Biosens. Bioelectron.* **2016,** *78*, 418-422.
57. Katsu, Y.;Iguchi, T., Cortisol. *Handbook of Hormones* **2021,** *2*, 947-949.

58. Li, J.; Liu, Y.; Ren, J.; Tay, B. Z.; Luo, T.; Fan, L.; Sun, D.; Luo, G.; Lau, D.; Marcos;Lam, R. H. W., Antibody-Coated Microstructures for Selective Isolation of Immune Cells in Blood. *Lab Chip* **2020,** *20*, 1072-1082.
59. Zhang, B.; Li, J.; Zhou, J.; Chow, L.; Zhao, G.; Huang, Y.; Ma, Z.; Zhang, Q.; Yang, Y.; Yiu, C. K.; Li, J.; Chun, F.; Huang, X.; Gao, Y.; Wu, P.; Jia, S.; Li, H.; Li, D.; Liu, Y.; Yao, K., et al., A Three-Dimensional Liquid Diode for Soft, Integrated Permeable Electronics. *Nature* **2024,** *628*, 84-92.
60. Young, E. A.; Abelson, J.;Lightman, S. L., Cortisol Pulsatility and Its Role in Stress Regulation and Health. *Front. Neuroendocrinol.* **2004,** *25*, 69-76.
61. Hsiao, H. Y.; Chen, R. L. C.; Chou, C. C.;Cheng, T. J., Hand-Held Colorimetry Sensor Platform for Determining Salivary Alpha-Amylase Activity and Its Applications for Stress Assessment. *Sensors* **2019,** *19*, 1571.
62. Pilozzi, A.; Carro, C.;Huang, X., Roles of Beta-Endorphin in Stress, Behavior, Neuroinflammation, and Brain Energy Metabolism. *Int. J. Mol. Sci.* **2021,** *22*, 338.
63. Yusni, Y.;Yusuf, H., the Acute Effects of Coffee Consumption on Blood Glucose and It's Relationship with Serum Cortisol and Insulin in Females. *Pharmacia* **2022,** *69*, 903-910.
64. Sevil, M.; Rashid, M.; Hajizadeh, I.; Park, M.; Quinn, L.;Cinar, A., Physical Activity and Psychological Stress Detection and Assessment of Their Effects on Glucose Concentration Predictions in Diabetes Management. *IEEE Trans. Biomed. Eng.* **2021,** *68*, 2251-2260.
65. Picard, M.; Juster, R. P.;McEwen, B. S., Mitochondrial Allostatic Load Puts the 'Gluc' Back in Glucocorticoids. *Nat. Rev. Endocrinol.* **2014,** *10*, 303-310.
66. Lopresti, A. L., The Effects of Psychological and Environmental Stress on Micronutrient Concentrations in the Body: A Review of the Evidence. *Adv. Nutr.* **2020,** *11*, 103-112.
67. Handbook of Psychocardiology. Springer Singapore: 2016.
68. Korhonen, I., Blood Pressure and Heart Rate Responses in Men Exposed to Arm and Leg Cold Pressor Tests and Whole-Body Cold Exposure. *Int. J. Circumpolar Health.* **2006,** *65*, 178-184.
69. Koban, L.; Gianaros, P. J.; Kober, H.;Wager, T. D., The Self in Context: Brain Systems Linking Mental and Physical Health. *Nat. Rev. Neurosci.* **2021,** *22*, 309-322.
70. Trotman, G. P.; Veldhuijzen van Zanten, J.; Davies, J.; Moller, C.; Ginty, A. T.;Williams, S. E., Associations between Heart Rate, Perceived Heart Rate, and Anxiety During Acute Psychological Stress. *Anxiety Stress Coping* **2019,** *32*, 711-727.
71. Pluntke, U.; Gerke, S.; Sridhar, A.; Weiss, J.;Michel, B., Evaluation and Classification of Physical and Psychological Stress in Firefighters Using Heart Rate Variability. In *019 41st Annual International Conference of the IEEE Engineering in Medicine and Biology Society (EMBC)*, Berlin, Germany, 2019; pp 2207-2212.
72. Santiago, E.; Poudyal, S. S.; Shin, S. Y.;Yoon, H. J., Graphene Oxide Functionalized Biosensor for Detection of Stress-Related Biomarkers. *Sensors* **2022,** *22*, 558.
73. Seoane, F.; Mohino-Herranz, I.; Ferreira, J.; Alvarez, L.; Buendia, R.; Ayllón, D.; Llerena, C.;Gil-Pita, R., Wearable Biomedical Measurement Systems for Assessment of Mental Stress of Combatants in Real Time. *Sensors* **2014,** *14*, 7120-7141.
74. Liu, J.; Xu, N.; Men, H.; Li, S.; Lu, Y.; Low, S. S.; Li, X.; Zhu, L.; Cheng, C.; Xu, G.;Liu, Q., Salivary Cortisol Determination on Smartphone-Based Differential Pulse Voltammetry System. *Sensors* **2020,** *20*, 1422.
75. Rice, P.; Upasham, S.; Jagannath, B.; Manuel, R.; Pali, M.;Prasad, S., Cortiwatch_Watch-Based Cortisol Tracker. *Future Sci. OA* **2019,** *5*, FSO416.
76. Kinnamon, D.; Ghanta, R.; Lin, K.-C.; Muthukumar, S.;Prasad, S., Portable Biosensor for Monitoring Cortisol in Low-Volume Perspired Human Sweat. *Sci. Rep.* **2017,** *7*, 13312.

77. Lee, H.-B.; Meeseepong, M.; Trung, T. Q.; Kim, B.-Y.;Lee, N.-E., A Wearable Lab-on-a-Patch Platform with Stretchable Nanostructured Biosensor for Non-Invasive Immunodetection of Biomarker in Sweat. *Biosens. Bioelectron.* **2020,** *156*, 112133.
78. Parlak, O.; Keene, S. T.; Marais, A.; Curto, V. F.;Salleo, A., Molecularly Selective Nanoporous Membrane-Based Wearable Organic Electrochemical Device for Noninvasive Cortisol Sensing. *Sci. Adv.* **2018,** *4*, eaar2904.
79. Minguillon, J.; Perez, E.; Lopez-Gordo, M. A.; Pelayo, F.;Sanchez-Carrion, M. J., Portable System for Real-Time Detection of Stress Level. *Sensors* **2018,** *18*, 2504.
80. Ku, M.; Kim, J.; Won, J.-E.; Kang, W.; Park, Y.-G.; Park, J.; Lee, J.-H.; Cheon, J.; Lee, H. H.;Park, J.-U., Smart, Soft Contact Lens for Wireless Immunosensing of Cortisol. *Sci. Adv.* **2020,** *6*, eabb2891.
81. Tang, W.; Yin, L.; Sempionatto, J. R.; Moon, J. M.; Teymourian, H.;Wang, J., Touch-Based Stressless Cortisol Sensing. *Adv. Mater.* **2021,** *33*, 2008465.
82. Huynh, V. L.; Trung, T. Q.; Meeseepong, M.; Lee, H. B.; Nguyen, T. D.;Lee, N. E., Hollow Microfibers of Elastomeric Nanocomposites for Fully Stretchable and Highly Sensitive Microfluidic Immunobiosensor Patch. *Adv. Func. Mater.* **2020,** *30*, 2004684.
83. Kim, H.; Kim, Y.-S.; Mahmood, M.; Kwon, S.; Epps, F.; Rim, Y. S.;Yeo, W.-H., Wireless, Continuous Monitoring of Daily Stress and Management Practice Via Soft Bioelectronics. *Biosens. Bioelectron.* **2021,** *173*, 112764.
84. Dong, Y.; Liu, T. L.; Chen, S.; Nithianandam, P.; Matar, K.;Li, J., A “Two-Part” Resonance Circuit Based Detachable Sweat Patch for Noninvasive Biochemical and Biophysical Sensing. *Adv. Func. Mater.* **2022,** *33*, 2210136.
85. Singh, N. K.; Chung, S.; Chang, A.-Y.; Wang, J.;Hall, D. A., A Non-Invasive Wearable Stress Patch for Real-Time Cortisol Monitoring Using a Pseudoknot-Assisted Aptamer. *Biosens. Bioelectron.* **2023,** *227*, 115097.
86. Valenti, S.; Volpes, G.; Parisi, A.; Peri, D.; Lee, J.; Faes, L.; Busacca, A.;Pernice, R., Wearable Multisensor Ring-Shaped Probe for Assessing Stress and Blood Oxygenation: Design and Preliminary Measurements. *Biosensors* **2023,** *13*, 460.
87. Su, T.; Mi, Z.; Xia, Y.; Jin, D.; Xu, Q.; Hu, X.;Shu, Y., A Wearable Sweat Electrochemical Aptasensor Based on the Ni–Co Mof Nanosheet-Decorated Cnts/Pu Film for Monitoring of Stress Biomarker. *Talanta* **2023,** *260*, 124620.
88. Fang, L.; Xing, S. P.; Long, Y.; Lee, K.-P.;Wang, S. J., Emosense: Revealing True Emotions through Microgestures. *Advanced Intelligent Systems* **2023,** *5*, 2300050.
89. Weng, G.; Yang, J.; Li, J.; Zhu, J.;Zhao, J., Ag Triangle Nanoplates Assembled on Pvc/Sebs Membrane as Flexible Sers Substrates for Skin Cortisol Sensing. *Spectrochimica Acta Part A: Molecular and Biomolecular Spectroscopy* **2023,** *303*, 123154.
90. Qin, J.; Wang, W.;Cao, L., Photonic Hydrogel Sensing System for Wearable and Noninvasive Cortisol Monitoring. *ACS Applied Polymer Materials* **2023,** *5*, 7079-7089.
91. Panicker, L. R.; Shamsheera, F.; Narayan, R.;Kotagiri, Y. G., Wearable Electrochemical Microneedle Sensors Based on the Graphene-Silver-Chitosan Nanocomposite for Real-Time Continuous Monitoring of the Depression Biomarker Serotonin. *ACS Applied Nano Materials* **2023,** *6*, 20601-20611.
92. Xu, B.; Chang, H.; Yang, G.; Xu, Z.; Li, J.; Gu, Z.;Li, J., An Integrated Wearable Sticker Based on Extended-Gate Algan/Gan High Electron Mobility Transistors for Real-Time Cortisol Detection in Human Sweat. *The Analyst* **2024,** *149*, 958-967.
93. Ankitha, M.; Shamsheera, F.;Rasheed, P. A., Mxene-Integrated Single-Stranded Carbon Yarn-Based Wearable Sensor Patch for on-Site Monitoring of Dopamine. *ACS Applied Electronic Materials* **2024,** *6*, 599-610.

94. Kim, M.; Joe, D. J.; Doh, I.;Cho, Y. H., Piezoelectric Nanocomposite-Based Multifunctional Wearable Bioelectronics for Mental Stress Analysis Utilizing Physiological Signals. *Adv. Mater. Technol.* **2024,** *9*, 2301610.
95. Wang, H.; Li, C.; Zhang, J.; Yang, Z.; Li, J.; Cao, Y.; Wu, K.; Liu, Z.; Hao, J.;Ye, X., Nir-Excitable Pom-Encapsulated Yb-Bi2s3 Decorated Graphene for Wearable Photoelectrochemical Sensing. *Adv. Func. Mater.* **2024,** *34*, 2315917.
96. Liu, K.; Wang, H.; Zhu, F.; Chang, Z.; Du, R.; Deng, Y.;Qi, X., Lab on the Microneedles: A Wearable Metal–Organic Frameworks-Based Sensor for Visual Monitoring of Stress Hormone. *ACS Nano* **2024,** *18*, 14207-14217.
97. Chakoma, S.; Pei, X.; Qin, H.; Ghandehari, A.; Najafikhoshnoo, S.; Rajendran, J.;Esfandyarpour, R., A Passive, Reusable, and Resonating Wearable Sensing System for on-Demand, Non-Invasive, and Wireless Molecular Stress Biomarker Detection. *Nano Res.* **2024,** *17*, 7542-7556.
98. Wang, C.; Wang, Z.; Wei, W.; Zhang, Z.; Li, A. A.; Huang, G.; Li, X.; Ge, S. S.; Zhou, L.;Kong, H., High-Precision Flexible Sweat Self-Collection Sensor for Mental Stress Evaluation. *npj Flex. Electron.* **2024,** *8*, 47.
99. Lo Grasso, A.; Zontone, P.; Rinaldo, R.;Affanni, A., Advanced Necklace for Real-Time Ppg Monitoring in Drivers. *Sensors* **2024,** *24*, 5908.
100. Ding, Y.; Tan, K.; Sheng, L.; Ren, H.; Su, Z.; Yang, H.; Zhang, X.; Li, J.;Hu, P., Integrated Mental Stress Smartwatch Based on Sweat Cortisol and Hrv Sensors. *Biosens. Bioelectron.* **2024,** *265*, 116691.
101. Abdul Kader, L.; Al-Shargie, F.; Tariq, U.;Al-Nashash, H., One-Channel Wearable Mental Stress State Monitoring System. *Sensors* **2024,** *24*, 5373.
